# Competitive interactions shape mammalian brain network dynamics and computation

**DOI:** 10.1038/s41593-026-02205-3

**Published:** 2026-03-11

**Authors:** Andrea I. Luppi, Yonatan Sanz Perl, Jakub Vohryzek, Hana Ali, Pedro A. M. Mediano, Fernando E. Rosas, Filip Milisav, Laura E. Suárez, Silvia Gini, Daniel Gutierrez-Barragan, Yohan Yee, Seán Froudist-Walsh, Alessandro Gozzi, Bratislav Misic, Gustavo Deco, Morten L. Kringelbach

**Affiliations:** 1 Department of Psychiatry and Centre for Eudaimonia and Human Flourishing, Linacre College, University of Oxford, Oxford, UK; 2 International Centre for Flourishing, Universities of Oxford, Oxford, UK; 3 St. John’s College, University of Cambridge, Cambridge, UK; 4 Division of Information Engineering, University of Cambridge, Cambridge, UK; 5 Montreal Neurological Institute, McGill University, Montreal, Quebec Canada; 6 International Centre for Flourishing, Universities of Aarhus, Aarhus, Denmark; 7 International Centre for Flourishing, Universities Pompeu Fabra, Barcelona, Spain; 8 Centre for Brain and Cognition, Pompeu Fabra University, Barcelona, Spain; 9 Department of Computing, Imperial College London, London, UK; 10 Department of Informatics, Sussex AI, and Sussex Centre for Consciousness Science, University of Sussex, Brighton, UK; 11 Principles of Intelligent Behavior in Biological and Social Systems, Prague, Czech Republic; 12 Centre for Neuroscience and Cognitive Systems, Italian Institute of Technology, Rovereto, Italy; 13 Centre for Mind/Brain Sciences, University of Trento, Trento, Italy; 14 Department of Radiology, University of Calgary, Calgary, Alberta Canada; 15 Bristol Computational Neuroscience Unit, Bristol University, Bristol, UK; 16 Catalan Institution for Research and Advanced Studies (ICREA), Barcelona, Spain; 17 Centre for Music in the Brain, Aarhus University, Aarhus, Denmark

**Keywords:** Biophysical models, Dynamical systems, Network models

## Abstract

How does brain network architecture balance cooperation and competition between distributed circuits? Here we use computational whole-brain modeling to examine the dynamical and computational relevance of cooperative and competitive interactions in the mammalian connectome. Across human, macaque and mouse, we show that to faithfully reproduce brain activity, model architecture consistently combines modular cooperative interactions with diffuse, long-range competitive interactions. Across species, competitive interactions preferentially link regions characterized by opposite profiles of cytoarchitecture, gene expression and receptor expression. The model with competitive interactions provides superior subject specificity, consistently outperforming the cooperative-only model and exhibiting excellent fit to the spatiotemporal properties of the living brain. These properties were not explicitly optimized, instead emerging spontaneously. Competitive interactions in the generative connectivity produce more synergistic and hierarchical dynamics, leading to enhanced performance for neuromorphic computing. Altogether, this work provides a generative link among network architecture, dynamical properties and computational performance in the mammalian brain.

## Abstract (web summary)

Brain network architecture may balance cooperation and competition across circuits. Here the authors use computational whole-brain modeling across three species to show that models with competition are more realistic, more personalized and perform better.

## Main

A central goal of neuroscience is to understand how the architecture of the brain governs information processing. To support cognition, the brain must orchestrate the constant competition between specialized functional circuits, each arising from the cooperation of anatomically distributed regions^1–4^. At the macroscale, spontaneous hemodynamics and electrodynamics provide evidence for both cooperative and antagonistic processes in the mammalian brain, exhibiting systematic and recurrent patterns of coordinated and anticoordinated activity^1,5–7^. Although the behavioral and physiological relevance of functional anticorrelations is well established, their mechanistic origin remains unclear^5^. How does the brain orchestrate its cooperative and antagonistic tendencies?

Interactions between brain regions unfold dynamically over a complex network of anatomical connections: the structural connectome^8–11^. To obtain mechanistic insight about how brain structure gives rise to function, connectome-based computational models of brain activity integrate neurobiological theory and data across scales and across imaging modalities^12–22^. Such generative models have provided growing insights about the role of regionally heterogeneous cytoarchitecture, myeloarchitecture and chemoarchitecture in shaping brain connectivity and dynamics, as well as the role of local and global network organization of the connectome and its variations related to evolution, development and disease^23–42^.

The vast majority of connectome-based generative models of brain activity—ranging from simple percolation models^36^ to Kuramoto and Hopf oscillators^38,43,44^ and more detailed Wilson–Cowan, Jansen–Rit or mean-field models^37,45–49^—assume that the long-range connectivity between brain regions represents cooperative interactions: that is, if region A is connected to region B, and A’s activity increases, then B’s activity will also increase. This longstanding assumption arguably arises because the main methods of reconstructing anatomical connectivity between regions (tract tracing and diffusion tractography) produce positively signed connectivity (but see the recent work of Tanner et al.^50^).

However, competitive interactions (whereby greater activity in unit A has a suppressive or ‘net-negative’ effect on unit B, leading to a decrease in B’s activity) are a ubiquitous principle of organization in dynamical systems, whether biological, social or artificial^51–64^. They serve fundamental purposes such as stabilization, feedback control and segregating processes to avoid interference. Indeed, it is well established that the brain makes extensive use of competitive interactions at the microscale—for example, in the form of inhibitory synapses governing the computational and dynamical properties of neuronal circuits^64–67^ (but please note that inhibition and competitive interactions are not synonymous: inhibition may be one way to implement competitive dynamics, but here we do not claim that every competitive interaction requires inhibition, nor that all inhibitory circuits are implementing competitive dynamics).

Here, we ask whether competitive interactions could also be present in the mammalian brain at the macroscale—and, if so, how do they shape brain dynamics? We investigate this question by developing a species-specific generative model of brain dynamics that allows both cooperative and competitive interactions in its generative connectivity. This enables us to quantitatively compare models with versus without competitive interactions in terms of their faithfulness to empirical recordings of functional magnetic resonance imaging (fMRI). Does the best-fitting account of brain activity involve competitive interactions? Subsequently, we directly compare the dynamical and computational properties of cooperative-only and cooperative–competitive models.

Because human in vivo diffusion tractography has known limitations that may, in turn, limit the validity of connectome-based models^68^, we follow a recent call for action and ‘assess the cross-species validity [of our modeling framework] in animal models for which structural connectivity has been obtained via invasive methods’^68^. Namely, we integrate species-specific fMRI with gold-standard tract-tracing data to generalize our findings about the human brain to macaque and mouse: two fundamental model organisms in translational neuroscience.

To foreshadow our results, we find that across human, macaque and mouse, the architecture of our best-fitting models consistently combines modular cooperative interactions with long-range competitive interactions, achieving up to a two-fold improvement in the fit between simulated and empirical functional connectivity. Across species, the resulting dynamics are also more synergistic and hierarchical, in line with empirical observations, and exhibit more realistic values of metastability, alternating between periods of integration and segregation. Although such properties are not explicitly optimized for, they emerge spontaneously as a consequence of including competitive interactions. In each of the three species, our framework demonstrates high reliability at both individual and group levels and also results in models that are significantly more subject specific. We conclude that our best model of how brain structure gives rise to brain function should include competitive interactions.

## Results

**Fig. 1:**
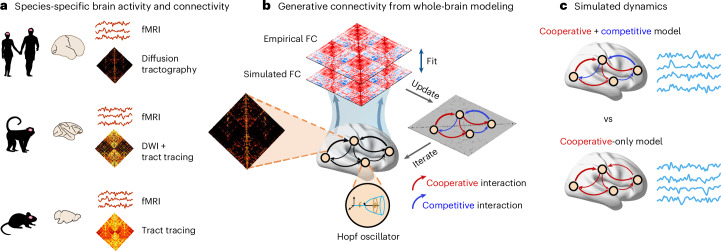
Whole-brain models generate brain activity from species-specific structural connectivity. **a**, We developed dedicated computational models of the human, macaque and mouse brains, based on species-specific structural connectivity: from each individual’s diffusion tractography (human); from diffusion tractography augmented with tract tracing from the CoCoMac database (macaque); and from full tract tracing (mouse). To facilitate comparison of results between human and other species, the structural connectomes of macaque and mouse were symmetrized, to avoid imposing structural asymmetries. Each model is fitted to species-specific fMRI recordings at the single-subject level (human: *n* = 100; macaque: *n* = 19; mouse: *n* = 10). **b**, Overview of modeling procedure. Each brain region is modeled as a Hopf oscillator poised near (but just below) the critical bifurcation, and regions are interconnected according to the wiring diagram specified by the species-specific SC. Connection weights are then iteratively and individually updated to improve the fit between empirical and simulated FC of each individual (both with and without lag). For the model that allows competitive interactions, the sign of connections is also allowed to vary. This means that competitive (that is, negative-signed) generative connectivity is allowed but not imposed. After convergence, the recovered weights indicate the level of coupling between regions that most faithfully reproduces the empirical FC, thereby representing the best estimate of how SC generates FC (hence the term ‘generative’ connectivity). **c**, After convergence, the generative connectivity obtained from the cooperative-only and cooperative–competitive models of each individual is used to generate brain activity, whose dynamical and computational properties are compared between the two models. DWI, diffusion-weighted imaging; FC, functional connectivity; SC, structural connectivity.

We set out to investigate the presence and dynamical role of competitive interactions between regions of the mammalian brain. To obtain insight about the potential generative mechanisms, we use computational whole-brain models of brain activity. At their core, such generative models comprise two key ingredients: (i) a mathematical representation of local dynamics and (ii) a wiring diagram of interregional coupling^16,22^. Specifically, to achieve a balanced tradeoff between simplicity and computational tractability on one hand, and realism and richness of the generated activity on the other hand, our model of local dynamics consists of nonlinear Stuart–Landau oscillators poised near (but just below) the critical Hopf bifurcation^69^ (Fig. 1).

Hopf models are widely used in neuroscience because when these models are expressly set on the edge of the bifurcation point and coupled according to the anatomical network wiring of the connectome, they have demonstrated excellent ability to reproduce key features of macroscale brain dynamics observed in electrophysiology^70,71^, magnetoencephalography^72^ and fMRI^43,44,73,74^. In particular, the Hopf model poised on the edge of bifurcation is the simplest model capable of exhibiting both asynchronous and synchronous (oscillatory) dynamics. In this regime, the Hopf model generates neither the single sustained oscillation of Wilson–Cowan and Kuramoto models nor the fully asynchronous activity of spiking and standard mean-field models, which lack oscillatory couplings, but, rather, a fluctuating stochastically structured signal with oscillatory components that matches the infra-slow fluctuations typically observed in fMRI signals^74–76^. Indeed, both direct comparisons with more biologically detailed biophysical models of excitatory and inhibitory neurons as well as data-driven inference of model parameters converge to indicate that the Hopf model at the edge of the critical bifurcation provides a suitable representation of resting-state fMRI signals^74–76^ (Supplementary Note 1).

To obtain species-specific models, we couple these oscillators according to distinct sources of data: subject-specific in vivo diffusion tractography (human), axonal tract tracing (mouse; ref. ^77^) and diffusion tractography augmented with axonal tract tracing from the CoCoMac database (macaque; ref. ^78^) (Supplementary Methods). Each model is fitted to species-specific fMRI recordings (Supplementary Methods) at the single-subject level (human: *n* = 100; macaque: *n* = 19 (ref. ^79^); mouse: *n* = 10 (ref. ^80^)) (Methods). In whole-brain modeling studies, it is common to optimize the model by tuning global or regional free parameters (for example, a global scaling factor for the entire structural connectivity). However, here we adopt a more recent approach introduced by refs. ^43,44,81^, which allows the connection weights themselves to vary individually (see Methods for full details). This method is more powerful because, rather than simply identifying the best-fitting value of a single global free parameter, it identifies the effective weighting of the existing anatomical connections that most faithfully reproduces the observed functional connectivity between them. In this way, this approach turns the initial structural connectivity into a generative coupling matrix (referred to as a ‘generative effective connectivity’ (GEC) in the original publications^43,81^, although it should be distinguished from the ‘effective connectivity’ produced by dynamic causal modeling^68,82,83^).

Crucially, we emphasize that the algorithm does not tune all possible connections between regions. Rather, as is common in the generative whole-brain modeling literature^68^, the model is based on empirical anatomical connectivity for each species. Anatomical connectivity is widely used to inform and constrain computational brain models, because ‘it is not unreasonable to assume that neural elements will tend to interact via prominent nerve tracts detectable at the macroscale’^68^. Concretely, the model is initialized from a structural connectivity matrix, and it is only allowed to update connections that are non-zero in the initial structural connectivity. Therefore, the anatomical connectivity imposes a biological constraint on the sparsity of the model-inferred generative connectivity: the model has to explain the spatiotemporal functional connectivity structure using the existing connections only^43,81^. Indeed, evidence suggests that integrating structural connectivity as a feature selector by allowing only certain connections to be included (such that non-zero functional connectivity between two regions is contingent on a corresponding non-zero structural connection) substantially improves connectivity models^43,44,68,81,84–86^. Note also that we do not use the structural connectivity just as a binary mask (as done, for example, in ref. ^50^). Rather, to preserve the rich biological information provided by the anatomical connectivity, the model is initialized from the original structural connectivity weights, such that the structural connectivity also provides a biological ‘prior’ on the weight of inferred generative connections^43,81,86^.

### Competitive interactions in the generative connectivity lead to more realistic structure–function relationships

Equipped with species-specific fMRI recordings and species-specific structural connectomes, we begin by asking: Does the most faithful account of brain activity involve competitive interactions? To allow competitive interactions in the model, we allow connections in the generative connectivity matrix to take both positive and negative values, whereas the cooperative-only model is obtained by allowing only positive values of generative connectivity.

We refer to positive-valued interactions in the generative connectivity as ‘cooperative’, because their net effect is that the more region A is active, the more its downstream neighbor B will have its activity increased, proportionally to the strength of the connection between A and B. Consequently, we refer to negative-valued interactions in the generative connectivity as ‘competitive’ (or suppressive), because greater activity in region A leads to lower activity in B, proportionally to the strength of the negative connection between them and vice versa, meaning that activity in one region acts to suppress the other. We emphasize that we do not equate competitive (that is, net-negative) interactions with neuronal inhibition, and our model is not meant to recover the excitatory or inhibitory synaptic polarity of neuronal projections^87^: the sign of the generative connections assigned by our model is functional in nature, and it reflects overall cooperative or competitive (suppressive) macroscopic interaction between two regions but not how such interaction is biologically implemented at the microscopic level. For example, antagonistic influence between two regions could conceivably be implemented by long-range inhibitory projections or by long-range excitation of a local inhibitory circuit, or more complex circuitry still^5,88–90^. However, competitive interactions could also arise through phenomena that need not invoke the concept of synaptic inhibition at all, including conduction delays or the relative contributions of cerebral blood volume and flow to the hemodynamic signal, among others^5,91,92^, or a combination of different implementation mechanisms in different regions. Our model is agnostic to how competitive interaction is implemented biologically and only reflects its ‘net-negative’ outcome of one region’s activity over another.

Crucially, we do not specify a priori which connections (if any) should be competitive rather than cooperative. Rather, the sign and weight are determined in a fully data-driven manner by the model. Therefore, we start by assessing whether, given the opportunity to use competitive interactions, the model will rely on them to improve its performance. Performance in this context refers to the degree to which the spatial organization of empirical functional connectivity is reproduced. Thus, the generative connectivity produced by the model represents our best inference of how structure generates function. If any competitive interactions are observed in the generative connectivity, it means that they contribute to accounting for the relationship between structure and function. The null hypothesis is, therefore, that the proportion of competitive interactions will not differ significantly from zero.

**Extended Data Fig. 1:**
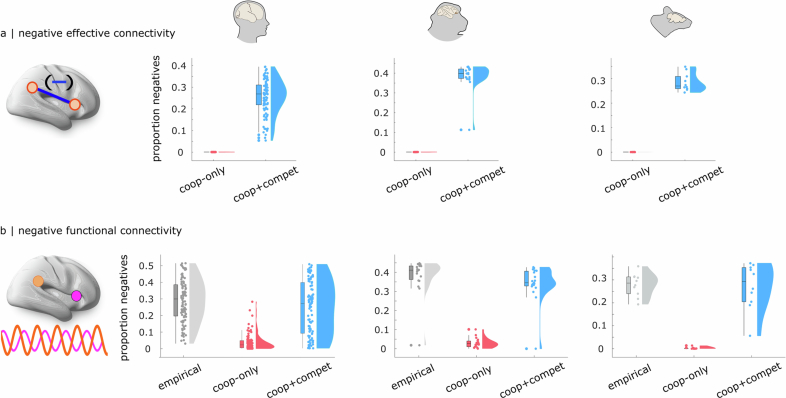
Prevalence of competitive interactions in the generative connectivity and negative functional connectivity. Prevalence of competitive interactions in the generative connectivity and negative functional connectivity | (**a**) As expected, the model that only allows cooperative interactions in the generative connectivity does not have any competitive interactions. In contrast, when the model is allowed to have both cooperative and competitive generative connectivity, we observe a non-zero proportion of competitive generative connections, in each individual of each species. Human: *n* = 100 individuals; macaque: *n* = 19 data-points from 10 animals; mouse: n = 10 animals. Box plots: the central lines indicate median values, the bounds of the boxes indicate the 25th and 75th percentiles, the whiskers indicate 1.5× the interquartile range. (**b**) Individual brains vary in the prevalence of anti-correlations in the empirical FC (though always non-zero). Even the model that only allows cooperative generative connectivity can produce some negative functional connectivity. However, the proportion of negative edges in the simulated FC is significantly higher, and closer to the empirical, when competitive generative connectivity is also allowed in the model. Human: *n* = 100 individuals; macaque: *n* = 19 data-points from 10 animals; mouse: *n* = 10 animals. See Supplementary Tables 1-3 for full statistical reporting. Box plots: the central lines indicate median values, the bounds of the boxes indicate the 25th and 75th percentiles, the whiskers indicate 1.5× the interquartile range. Source data are provided as a Source Data file. Source data

Our results show that we can conclusively reject this null hypothesis. When generative connectivity is allowed to take negative values (that is, the model is allowed to include competitive interactions), the model consistently takes advantage of this possibility (Extended Data Fig. 1 and Supplementary Tables 1–3). Across species, approximately 25–40% of edges in the generative connectivity are negative (human: 25 ± 8%; macaque: 38 ± 7%; mouse: 28 ± 4%) (Extended Data Fig. 1a). Notably, we find negative-valued connections in the inferred generative connectivity of every single individual (Extended Data Fig. 1a). We emphasize that this is not trivial: the model is allowed to have negative generative connectivity but it is not obligated to do so, if doing so would decrease the fit. It is entirely plausible that the models with and without competitive interactions could have ended up producing identical-looking networks of inferred generative connectivity, if it had been the case that competitive interactions serve no useful role for fitting the zero-lag and lagged functional connectivity. However, this is not what we observed empirically. Instead, the model consistently settles on having negatively valued generative connectivity, in its effort to best capture the empirical functional connectivity.

This model inversion procedure suggests that within the present modeling framework, our best-fitting account of how function arises from structure should incorporate competitive interactions. Crucially, note that the model is optimized by updating each (existing) structural connection individually, to reduce the discrepancy between the value of the corresponding entry in the simulated functional connectivity versus the empirical functional connectivity. However, each generative connection also plays a more global role, because A’s inputs to B spread to B’s neighbors in turn. Conceivably, updating the generative connection between A and B could have detrimental effects on the functional connectivity between A and a third region C that is connected to B, such that optimization of individual structural connections could lead to a globally suboptimal solution.

To dispel this possibility, we directly compare the outputs of two models: one that allows both cooperative and competitive interactions, and one that allows only cooperative interactions (corresponding to the traditional Hopf whole-brain model). Both models (with or without competitive interactions) are optimized to reproduce empirical functional connectivity and lagged functional connectivity and are allowed to run until no further improvement is observed. Note that allowing competitive interactions is not an additional free parameter but, rather, an expansion of the range of an existing parameter of the model.

**Fig. 2:**
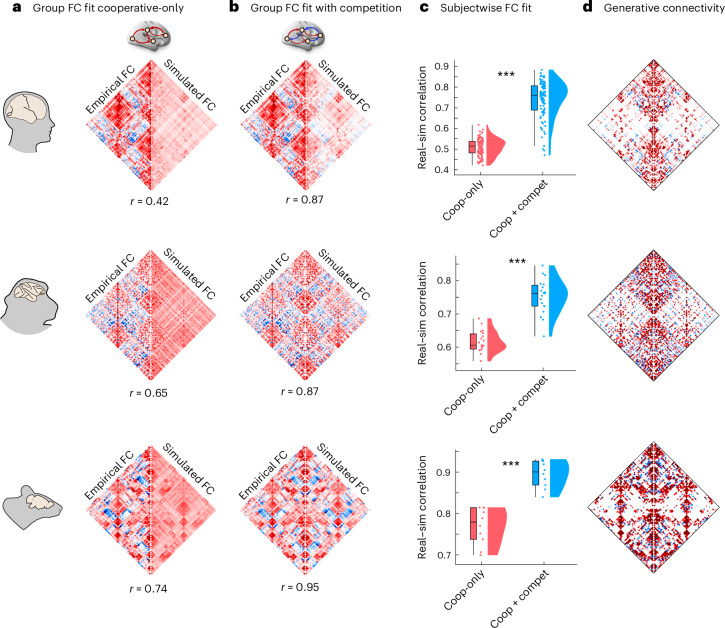
Generative competitive interactions lead to superior model fit across mammalian brains. **a**, Species-specific matrix showing the group-average empirical (left half) and simulated (right half) FC, for the model with only positive interactions. **b**, Species-specific matrix showing the group-average empirical (left half) and simulated (right half) FC, for the model allowing both cooperative and competitive interactions. The correlation coefficient between empirical and simulated groupwise FC is shown underneath each matrix. **c**, Fitting quality (correlation between empirical and simulated FC) at the level of individual subjects is significantly higher when competitive interactions are allowed. ***P < 0.001 from paired-sample *t*-tests (two-sided). Box plots: the central lines indicate median values; the bounds of the boxes indicate the 25th and 75th percentiles; and the whiskers indicate 1.5× the interquartile range. Each data point represents one individual scan. Human: *n* = 100 individuals; macaque: *n* = 19 data points from 10 animals; mouse: *n* = 10 animals. **d**, Species-specific matrix of GC averaged across subjects, for the model allowing negative interactions. Source data are provided as a Source Data file. FC, functional connectivity; GC, generative connectivity. Source data

Across all three species, we find that the improvement in model fit from competitive interactions is not just local but also global. Specifically, we observe a significant increase in the model’s ability to reproduce empirical functional connectivity (quantified as the correlation coefficient between simulated and empirical functional connectivity, which is commonly used in whole-brain modeling studies): both at the group level (Fig. 2a,b) and even at the level of individual subjects (Fig. 2c; human: mean (s.d.) = 0.51 ± 0.041 for cooperative-only; 0.74 ± 0.08 for cooperative–competitive; *t*_99_ = −26.40; *P*< 0.001; Hedge’s *g* = −3.33; macaque: mean (s.d.) = 0.61 ± 0.032 for cooperative-only; 0.75 ± 0.05 for cooperative–competitive; *t*_18_ = −9.06; *P* < 0.001; Hedge’s *g* = −3.04; mouse: mean (s.d.) = 0.70 ± 0.044 for cooperative-only; 0.89 ± 0.03 for cooperative–competitive; *t*_9_ = −5.92; *P* < 0.001; Hedge’s *g* = −3.06).

This improvement in global fit is so remarkable as to be visible with the naked eye: for both the mouse and macaque, the groupwise functional connectivity generated by the model with both cooperative and competitive interactions in the generative connectivity is visually indistinguishable from the empirical functional connectivity (Fig. 2b). This is confirmed numerically, with empirical and simulated groupwise functional connectivity exhibiting correlations of 0.87 (macaque) and 0.95 (mouse). By contrast, clear differences between real and simulated functional connectivity are noticeable for the model with positive-only generative connectivity (Fig. 2a). Although less visually striking, the human results are remarkable because they show the largest improvement: the model with competitive interactions more than doubles the group-level correlation between empirical and simulated functional connectivity, going from 0.42 (traditional Hopf model with cooperative-only interactions) to 0.87 (cooperative–competitive). This high correlation between empirical and predicted functional connectivity compares favorably to other generative, statistical and communication models for linking structure and function^11^.

In turn, the presence of competitive interactions in the generative connectivity (Fig. 2d) translates to a substantially higher prevalence of negative edges (anticorrelations) in the functional connectivity, much closer to the level observed in empirical functional connectivity across species (Extended Data Fig. 1a,b; see Supplementary Tables 1–3 for full statistical reporting). To be clear, the model with exclusively positive generative connectivity can also produce negative functional connectivity values (Extended Data Fig. 1b), up to 30% in our experience, although it remains unclear whether a theoretical upper bound exists. However, the prevalence of negative functional connectivity is substantially higher (and close to empirically observed levels) in the model that also allows competitive interactions (Extended Data Fig. 1 and Supplementary Tables 1–3).

Notably, the model with competitive interactions is also better able to capture interregional coordination in terms of the mutual information between pairs of regions (Supplementary Fig. 1). Unlike functional connectivity, mutual information is strictly non-negative. Thus, it is not the case that allowing negative weights in the generative connectivity improves the fit simply because the fitting target includes negative values. Rather, adding competitive interactions improves quantification of interregional interactions.

### Consistent organization of competitive interactions in the mammalian brain

**Fig. 3:**
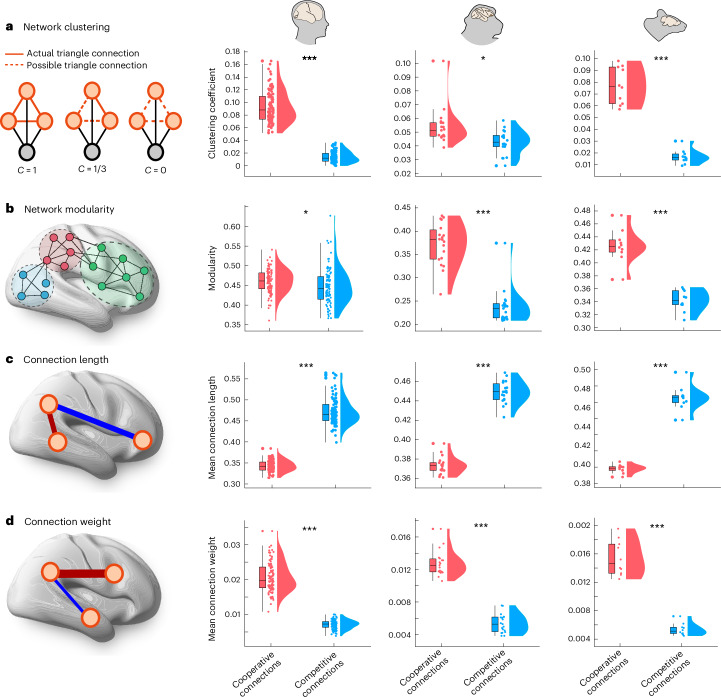
Network properties of the cooperative and competitive interactions in the generative connectivity. Competitive connections are significantly less clustered (**a**) and modular (**b**) than cooperative connections. Competitive connections are also longer, connecting regions that are further apart from each other in space (**c**) but tend to be weaker in weight (**d**). For **c**, length is the Euclidean distance between region centroids; for ease of comparison across species, length is normalized by the maximum distance within each species. See Supplementary Tables 1–3 for full statistical reporting. In **a**–**d**, box plots: the central lines indicate median values; the bounds of the boxes indicate the 25th and 75th percentiles; and the whiskers indicate 1.5× the interquartile range. Each data point represents one individual scan. Human: *n* = 100 individuals; macaque: *n* = 19 data points from 10 animals; mouse: *n* = 10 animals. All statistics come from paired-sample *t*-tests (two-sided). *P < 0.05, ***P < 0.001 from independent-sample *t*-tests (two-sided). Source data are provided as a Source Data file. Source data

Do positive and negative edges in the generative connectivity differ only in terms of sign, or are there systematic differences in their organization? We find high consistency across all three species, despite making use of different methods for reconstructing anatomical connectivity (subject-specific in vivo diffusion MRI tractography in humans; ex vivo tract tracing in mouse; and a combination of the two in macaque). Across species, competitive (negative) connections are weaker in magnitude and less prevalent than cooperative connections, but their placement is consistent across individuals, because they can be observed even in the group-average generative connectivity (Fig. 2d). Furthermore, the topological arrangement of competitive connections exhibits consistent features across all three species: (1) competitive connections are, on average, longer than cooperative ones (that is, they connect regions that are farther apart than the regions connected by positive weights) (Fig. 3c,d and Supplementary Tables 1–3); (2) negative generative connections are less modular than positive connections (Fig. 3b and Supplementary Tables 1–3); and (3) competitive connections exhibit a lower clustering coefficient (Fig. 3a and Supplementary Tables 1–3)—in other words, it is less likely that competitively interacting neighbors of a node will themselves be competitively interacting. This finding of lower clustering for the negative as compared to the positive connections echoes recent results by Tanner et al.^50^, who used a multiple-regression framework to assign sign and weight to anatomical connections and reported that positive connections tend to form dense triangles and cliques around nodes at a greater rate than negative connections.

Overall, the picture emerges of a modular network of strong positive ties alongside long-range, diffuse negative ties. However, the lower magnitude of negative generative weights should not be mistaken for low importance. To demonstrate this, we start from the fitted generative connectivity and selectively remove an increasing proportion of negative weights, to assess their role on the model fit. We find that negative weights, although weaker in magnitude, are key for boosting the model’s ability to reproduce biological fMRI signals, and their removal quickly deteriorates model fit (Supplementary Fig. 2).

The cross-species consistency of the competitive generative connections’ arrangement raises the possibility that they may coincide with biological principles of large-scale cortical organization. Specifically, cortical regions are heterogeneous and can exhibit similar or different patterns of many biological properties^93–95^. Among them, we include (1) gene expression (from human microarray, macaque Stereo-seq and mouse in situ hybridization); (2) cytoarchitecture (data about cell type composition available in all three species); (3) receptor expression (from in vivo positron emission tomography in humans^96^ and from in vitro receptor autoradiography in macaques^97^); and (4) laminar structure (also known as ‘microstructure profile covariance’), obtained from ex vivo histology of a human brain^98^. See Supplementary Methods for details of the original studies. Additionally, for each species we consider (5) the anatomical hierarchy from in vivo myeloarchitecture (T1-weighted/T2-weighted (T1w/T2w) MRI ratio^99,100^) and (6) the transcriptomically derived gradient of parvalbumin (PV) to somatostatin (SST) distribution, which delineates a gradient from sensory motor areas dominated by output-modulating PV-positive interneurons to association areas dominated by input-modulating SST-positive interneurons^101^.

For each of these modalities of biological cortical organization, we can obtain a matrix of similarity (covariance) between pairs of regions, indicating whether their biological annotations are similar (positive covariance) or opposite (negative covariance). We can then ask whether negative entries in the generative connectivity co-localize with negative entries in each dimension of cortical covariance more often than we should expect if the sign assigned to generative connectivity edges were random. That is, the null hypothesis is that there is no systematic pattern concerning the location of negative generative connectivity values in the matrix, with respect to that of biological annotations.

**Fig. 4:**
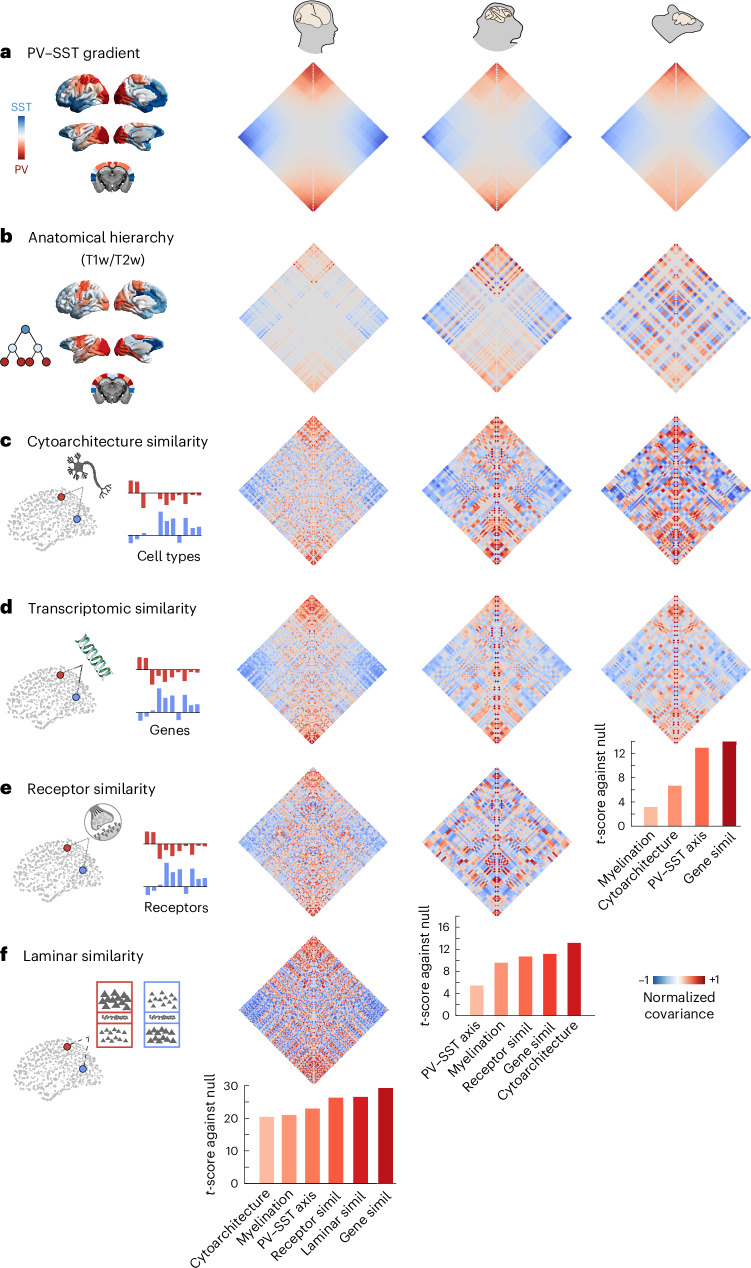
Competitive interactions link regions with opposite biological annotations. **a**, Brain plots depict species-specific axis from output-modulating PV-positive interneurons to input-modulating SST-positive interneurons, quantified as the difference in rank between *PVALB/Pvalb* and *SST/Sst* gene expression in each species (‘PV–SST axis)’. Matrix displays the similarity between regions’ locations along the PV–SST axis, obtained as the outer product of the *z*-scored PV–SST vectors, such that regions at opposite ends of the axis will have a negative link between them. **b**, Brain plots depict species-specific anatomical hierarchy quantified by intracortical myelination from T1w/T2w MRI. Matrix displays the similarity between regions’ locations along the anatomical hierarchy, obtained as the outer product of the *z*-scored T1w/T2w vectors, such that regions at opposite ends of the hierarchy will have a negative link between them. **c**, Cytoarchitectonic covariance. See Supplementary Methods for details of transcriptomically derived maps of regional cell type composition. Normalized patterns of cell composition were correlated across regions to obtain a covariance matrix. **d**, Covariance of regional gene expression from the Allen Institute for Brain Science microarray (human); Stereo-seq gene expression (macaque) from the Brain Science Data Center, Chinese Academy of Sciences (macaque); and in situ hybridization from the Allen Institute for Brain Science (mouse). To ensure consistency, we included 81 genes that are available in all three species (Supplementary Methods). Normalized patterns of gene expression were correlated across regions to obtain a covariance matrix. **e**, Covariance of regional receptor density: across 19 receptors and transporters from in vivo positron emission tomography (human) and across 13 receptors from ex vivo autoradiography (macaque). Note that not all regions of the macaque cortex have available data. Normalized patterns of receptor density were correlated across regions to obtain a covariance matrix. **f**, Covariance of regional laminar profiles (also known as microstructure profile covariance). Ex vivo laminar structure is estimated from the BigBrain, a high-resolution (20-µm) histological reconstruction of a postmortem human brain. The region-by-region laminar covariance matrix was calculated as the partial correlation of cell intensities between pairs of cortical regions, after correcting for the mean intensity across cortical regions. Adapted from ref. ^98^. For visualization purposes, all matrices are sorted according to the PV–SST axis to highlight consistency. See Supplementary Methods for details of each data modality in each species. Bar plots indicate the *t*-score obtained from comparing the prevalence of overlaps between negative connections in the inferred connectivity matrix, and in each of the biological networks, against the overlap observed if the connection sign were random. See Supplementary Fig. 3 for individual violin plots and Supplementary Tables 1–3 for full statistical reporting. Source data are provided as a Source Data file. Source data

We find that we can consistently reject this null hypothesis, implemented by counting how often negative edges co-occur in the generative connectivity matrix and each biological annotation matrix, against a null model of randomly swapped edge signs. Across species, competitive connections preferentially occur between regions that belong to opposite ends of the anatomical cortical hierarchy and to opposite ends of the PV–SST (that is, output-modulating to input-modulating) hierarchy (Fig. 4). Likewise, for each modality of biological annotation considered here, we systematically find that negative generative connections preferentially link regions that have opposite (anticorrelated) cytoarchitectonic, transcriptomic, laminar and receptor profiles (Fig. 4). This cross-species, cross-modal consistency provides robust evidence that the placement of competitive interactions in the generative connectivity inferred by our model is both systematic and biologically meaningful.

### Competitive interactions lead to models with superior generalizability and superior subject specificity

**Fig. 5:**
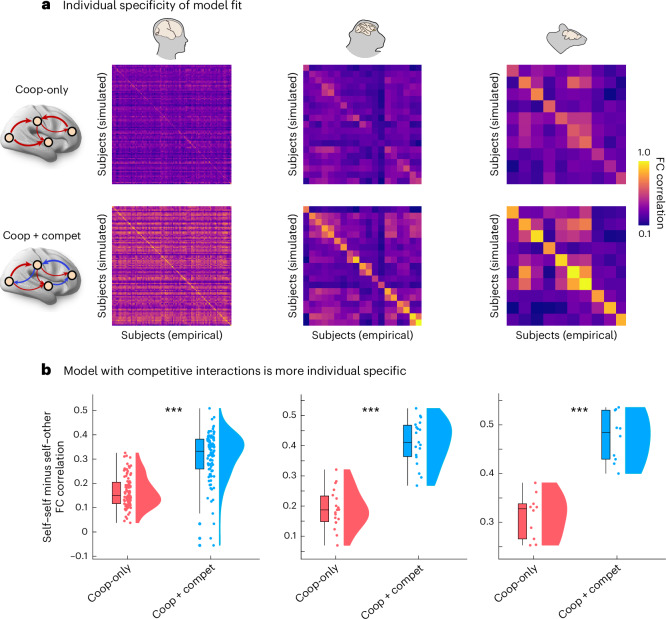
Competitive interactions produce models with greater subject specificity. **a**, Matrix of FC similarity (correlation) between each empirical subject (columns) and each simulated subject (rows). Brighter diagonal therefore reflects greater success of the commonly used model-fitting criterion of similarity between a subject’s own empirical and simulated FC (as reported in Fig. 2). **b**, Differential identifiability is defined as the difference between self–self similarity of FC (diagonal elements in **a**) and mean self–other similarity of FC (off-diagonal elements), such that greater difference implies more individual specificity. In other words, it quantifies the cost of mismatching individuals. When this cost is low, there is low subject specificity. In the ‘brain fingerprinting’ literature, differential identifiability quantifies the ease of telling apart different individuals based on their FC; here, we apply it to quantify the individual specificity of brain models, such that greater differential identifiability indicates that there is a larger drop in similarity between simulated and empirical FC when a model is matched to the wrong individual. We find that models with both cooperative and competitive interactions are significantly more individual specific. ***P < 0.001 from paired-sample *t*-tests (two-sided). Box plots: the central lines indicate median values; the bounds of the boxes indicate the 25th and 75th percentiles; and the whiskers indicate 1.5× the interquartile range. Each data point represents one individual. Human: *n* = 100 individuals; macaque: *n* = 19 data points from 10 animals; mouse: *n* = 10 animals. Source data are provided as a Source Data file. FC, functional connectivity. Source data

Additionally, we find that the model with competitive interactions is not only better able to reflect the empirical functional connectivity that it is intended to model, but is also better at distinguishing between the functional connectivity patterns of different individuals (Fig. 5). Arguably, a model could be very good, but also very unspecific, if it exhibited equally good fit to the functional connectivity of every individual—for example, by only capturing elements that are common across all brains while disregarding subject-specific ones. This concern may be especially prominent for our macaque and mouse models, which are based on species-specific connectomes rather than individual-specific ones (whereas the human models were fitted using individualized structural connectomes).

To investigate this question, we turn to the literature on ‘brain fingerprinting’^102–104^ and produce, for each species’s dataset, an identifiability matrix, quantifying the similarity (correlation) between the functional connectivity of each empirical subject and each simulated subject. We find that with the addition of competitive interactions, the model improves its ability to fit the individual who provided the data (self–self similarity) but also its ability to generalize to other individuals (self–other similarity) (Supplementary Fig. 4). Increased self–other correlation indicates that the cooperative–competitive model is better able to generalize to other individuals (that is, other biological brains). This should not be observed if the model were just overfitting to the idiosyncrasies of each individual and, therefore, improving its performance just by learning noise, instead of learning about brain organization. Crucially, however, the improvement in self–self similarity outstrips the improvement in self–other similarity, meaning that the cooperative–competitive model is overall more subject specific. We formally quantify this phenomenon using the measure of ‘differential identifiability’ from the literature on brain fingerprinting, which is defined as the mean difference between self–self and self–other similarity of functional connectivity^102^. In other words, this measure quantifies the cost of mismatching individuals in terms of loss of model fit. For both models (with and without competitive interactions), we find that differential identifiability is always positive (Fig. 5a): the model reproducing an individual’s functional connectivity exhibits greater affinity for the empirical functional connectivity of that individual than for the functional connectivity of other individuals. Crucially, however, we also find that the model with competitive interactions exhibits significantly larger differences between self–self and self–other functional connectivity fit (Fig. 5b; human: mean (s.d.) = 0.16 ± 0.06 for cooperative-only; 0.31 ± 0.09 for cooperative–competitive; *t*_99_ = −15.05; *P* < 0.001; Hedge’s *g* = −1.76; macaque: mean (s.d.) = 0.19 ± 0.063 for cooperative-only; 0.41 ± 0.07 for cooperative–competitive; *t*_18_ = −12.29; *P* < 0.001; Hedge’s *g* = −3.12; mouse: mean (s.d.) = 0.31 ± 0.045 for cooperative-only; 0.47 ± 0.05 for cooperative–competitive; *t*_9_ = −9.99; *P* < 0.001; Hedge’s *g* = −3.19). In other words, there is a larger drop in similarity between simulated and empirical functional connectivity, when a model is matched to the wrong individual. This effect is consistently observed across species. Note that, for the human data, both the cooperative-only model and the cooperative–competitive model are based on individual structural connectivity; therefore, the use of individual structural connectivity cannot be driving differences in the two models’ performance. Thus, observing increased self–other correlation and increased differential identifiability allows us to conclude that the model with competitive interactions is both significantly more generalizable and significantly more individual specific.

### Dynamical consequences of competitive interactions in the generative connectivity

We have shown that competitive interactions in the generative connectivity shape the ‘spatial’ organization of functional connectivity. However, mammalian brains also exhibit rich dynamics, giving rise to prominent patterns of ‘temporal’ signal coordination^20^. Therefore, the question arises: Do competitive interactions also shape brain dynamics?

**Fig. 6:**
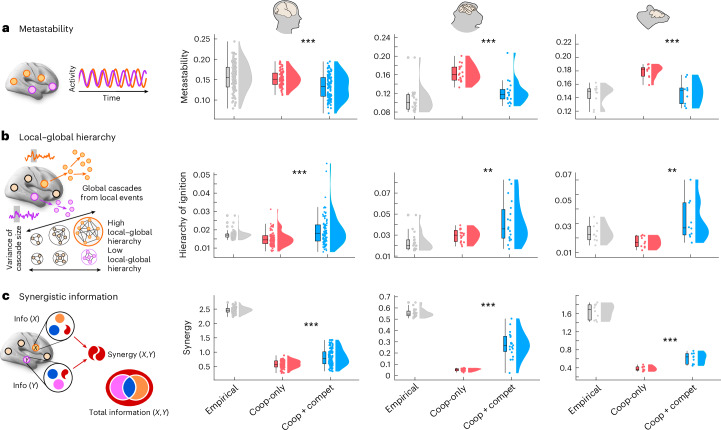
Dynamical consequences of competitive interactions in the generative connectivity. **a**, Metastability. Metastability (here, operationalized as the temporal standard deviation of the KOP) reflects the temporal alternation of synchronization and desynchronization across the brain. Human: *n* = 100 individuals; macaque: *n* = 19 data points from 10 animals; mouse: *n* = 10 animals. **b**, Local–global hierarchy. Intrinsic-driven ignition (IDI) is obtained by identifying ‘driver events’ (unusually high fMRI spontaneous activity) and measuring the number of co-occurring events. A measure of local–global hierarchy is obtained by calculating the variability across regions of their average IDI size over time, such that the brain is more hierarchical when there is greater disparity across the size of elicited intrinsic neural events. Human: *n* = 100 individuals; macaque: *n* = 19 data points from 10 animals; mouse: *n* = 10 animals. **c**, Synergistic information. The total information jointly carried by two variables *X* and *Y* (for example, two brain regions) can be exhaustively decomposed into information that is redundantly carried by both variables (blue) or uniquely by each (violet and orange) or synergistically by considering the two variables together (red). In **a**–**c**, human: *n* = 100 individuals; macaque: *n* = 19 data points from 10 animals; mouse: *n* = 10 animals. Each column displays results for a different species. *P < 0.05, **P < 0.01 and ***P < 0.001 from paired-sample *t*-tests (two-sided). Box plots: the central lines indicate median values; the bounds of the boxes indicate the 25th and 75th percentiles; and the whiskers indicate 1.5× the interquartile range. Each data point represents one individual scan. Full statistical reporting for the comparisons between cooperative-only and cooperative–competitive models is provided in Supplementary Tables 1–3. Source data are provided as a Source Data file. KOP, Kuramoto order parameter. Source data

Notably, we find that, in addition to achieving substantially better spatial similarity with the empirical functional connectivity, the model with competitive interactions also exhibits more realistic dynamics, which it was not explicitly optimized for. To demonstrate this, we use the generative connectivity recovered by each model (with and without competitive interactions) to simulate regional brain activity (hence, switching from inverse modeling to forward modeling^17^). One aspect of brain dynamics that is often studied is metastability, which, at the macroscale, is typically operationalized as the temporal standard deviation of the Kuramoto order parameter (KOP^73,105^; see ref. ^106^ for an extensive discussion of metastability and its alternative signatures in the literature). Because the KOP quantifies instantaneous synchrony, its variability over time quantifies the tendency of the brain to alternate between periods of synchronization and desynchronization, reflecting the co-existence of integrative and segregative tendencies^106^. Results show that metastability (std(KOP)) exhibits unrealistically high values for the model with positive-only interactions in the generative connectivity (Fig. 4a). By contrast, including competitive interactions leads to a more balanced level of metastability, closer to empirical values (Fig. 6a; see Supplementary Tables 1–3 for full statistical reporting).

Our results also show that the model with competitive interactions in its generative connectivity exhibits a more hierarchical organisation. ‘Intrinsic-driven ignition’ (IDI) refers to the capacity of local events of unusually high activity to propagate globally throughout the brain, which is thought to be necessary for allowing integration and differentiation to co-exist in the brain^107,108^. Building on this principle, a measure of local–global hierarchy can then be obtained by comparing the variability of regions’ capacity to ignite global activity: the brain is more hierarchical when there is greater disparity across regions for the size of elicited intrinsic neural events. In other words, local–global hierarchy is operationalized in terms of covering a broader range from local to global size of intrinsically originated events (Fig. 6b): when event sizes are all near equal (whether all very localized or all very global), there is low hierarchy, whereas, when there are wide differences in event size across regions, then there is a high level of local–global hierarchy in the brain^107,108^. We find that this measure is consistently increased for the model with versus without competitive interactions (Fig. 6b and Supplementary Tables 1–3). We obtain analogous results using an alternative way of conceptualizing hierarchical organization in the brain, related to the temporal (ir)reversibility of brain activity, which gives rise to asymmetric interactions between brain regions^43,109^. When interactions are symmetric, then sending and receiving of information is balanced for each region, whereas, when interactions are asymmetrical, then directionality exists, such that regions will differ in terms of whether they have a preference for sending or receiving signals from other regions. Hence, the greater the variability in this send–receive asymmetry across regions, the more the system’s organization is hierarchical—that is, departing from the balance of a flat organization^43^. Our results show that the presence of competitive interactions in the generative connectivity induces more hierarchical organization of asymmetry, with greater divergence in regions’ preference for sending or receiving information, being closer to what is observed in empirical brain dynamics (Supplementary Fig. 5). We note that both the models with and without competitive interactions include lag-1 functional connectivity in their fitting function, thereby incorporating some aspect of directionality and irreversibility, as previously recommended^43,44,84^. Nonetheless, we see that the model with competitive interactions is better capable of reflecting variability of send–receive asymmetry across regions compared to the model that allows only positive values of generative connectivity (Supplementary Fig. 5; see Supplementary Tables 1–3 for full statistical reporting).

Finally, as a further assessment of brain dynamics, we consider the prevalence of synergistic information in the intrinsic dynamics of the brain^110^. In a dynamical system such as the brain, activity is not random: even at rest (that is, in the absence of any explicit task), the future state of the brain’s spontaneous activity will depend on its past state. This means that the past trajectory of a region’s activity holds information about its future state, known as ‘time-delayed’ mutual information. However, brain regions are interconnected and continuously interacting, such that the activity of region *X* may also influence the future activity of region *Y* and vice versa. Mathematically, the total information jointly carried by two variables *X* and *Y* (here, two brain regions) can be exhaustively decomposed into information that is carried redundantly by both variables (such that it is equally available from each of them); information that is carried uniquely by only one variable; and, finally, information that is carried by the two variables synergistically^110–114^. Synergistic information is available only when both variables are considered jointly but not when considering either variable in isolation, thereby reflecting the extent to which interactions in the system are ‘greater than the sum of their parts’. It was recently shown that synergy is associated with higher-order cognitive operations and evolutionary expansion of the human brain, being also diminished when consciousness is lost due to pharmacological or pathological conditions^110,111,115^. Our results show that although both models fall short of the level of synergy observed in empirical brains, the model with competitive interactions produces significantly higher values of synergy in its dynamics, consistently exceeding the model with positive-only interactions, across all three mammalian species considered (Fig. 6c and Supplementary Tables 1–3).

### Competitive interactions increase the match between simulated brain activity and canonical cognitive operations of the human brain

Up to this point, we have found that allowing the generative connectivity to include competitive (negative-valued) interactions leads to significantly greater fidelity to the spatial and temporal organization of the mammalian brain. Ultimately, the goal of spatial and temporal coordination between brain regions is to support cognition. To support different cognitive roles, brain regions co-activate together to form specialized functional circuits. Such coherent patterns of co-activation can be observed even in the spontaneous activity of the brain, suggesting that they are an intrinsic feature of brain functional organization^116^. Does the cooperative–competitive model also exhibit the spontaneous emergence of coherent functional circuits?

**Fig. 7:**
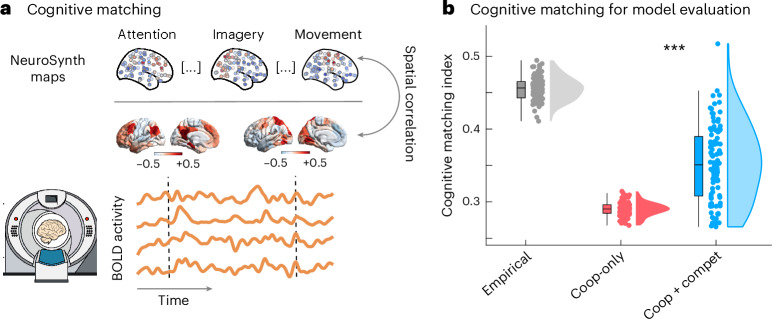
Competitive interactions increase the match between simulated brain activity and canonical cognitive operations of the human brain. **a**, At each point in time, the cognitive matching score is computed as the best spatial correlation between instantaneous brain activity and 123 NeuroSynth meta-analytic brain maps. For each individual (real or simulated), an overall index of the quality of cognitive matching is then obtained by averaging the cognitive matching scores across the entire scan duration. Higher NeuroSynth matching indicates greater alignment of spontaneous brain activity with brain maps from the cognitive neuroimaging literature. **b**, Model with negative generative connectivity produces patterns of brain activity that are significantly more realistic than when only positive generative connectivity is allowed. ***P < 0.001 from paired-sample *t*-tests (two-sided) between cooperative-only and cooperative–competitive. Box plots: the central lines indicate median values; the bounds of the boxes indicate the 25th and 75th percentiles; and the whiskers indicate 1.5× the interquartile range. Each data point represents one individual (*n* = 100 simulations corresponding to 100 human individuals, for each condition). Source data are provided as a Source Data file. Source data

To quantify how well the instantaneous patterns of brain activity match the brain patterns associated with canonical cognitive operations, we use our recently introduced ‘cognitive matching’ procedure^103^. The cognitive matching score is computed as the best spatial correlation between brain activity and 123 brain maps obtained by meta-analytic aggregation of thousands of human neuroimaging studies from the NeuroSynth engine and the Cognitive Atlas (Fig. 7a)^103,117^. For each individual (real or simulated), an overall index of the quality of cognitive matching is obtained by averaging the cognitive matching scores across the entire scan duration. Higher match to NeuroSynth meta-analytic maps indicates greater alignment of spontaneous brain activity with brain maps from the cognitive neuroimaging literature. In somewhat poetic terms, cognitive matching quantifies how ‘mind-like’ the observed brain patterns are. Indeed, we recently showed that the quality of cognitive matching declines sharply in response to anesthesia when consciousness and cognition are suppressed^103^.

Thus, through cognitive matching, we can assess whether our computational models co-activate regions that we know belong to the same cognitive circuit in the real brain (as indicated by meta-analytic co-activation). Our results show that the cognitive matching index of the model with competitive interactions is significantly higher than the one of the model with only cooperative interactions (mean (s.d.) = 0.29 ± 0.01 for cooperative-only; 0.35 ± 0.05 for cooperative–competitive; *t*_99_ = −14.12; *P* < 0.001; Hedge’s *g* = −1.68), eliciting a substantial shift toward the levels of cognitive matching observed for the real human brain (Fig. 7b). In other words, the model with competitive interactions is more capable to co-activate regions that belong to the same functional circuit, as defined in a data-driven manner from thousands of cognitive neuroscience experiments. (Note that, at present, NeuroSynth is only available for human neuroimaging studies, making this assessment of model quality possible only for the case of human brain activity.)

### Competitive interactions increase computational capacity

Despite being termed ‘functional’, the functional connectivity need not reflect any genuine function, in the sense of ongoing cognition^118^. Our cognitive matching procedure improves on this shortcoming, by associating specific patterns of brain activity with specific cognitive operations. An alternative, complementary strategy is the recently introduced approach of connectome-based neuromorphic computing^119–121^. Under this framework, a connectome is used as the network wiring diagram in a reservoir computing artificial neural network performing a specific task, such as memorizing some time series (note that this is an artificial neural network, not a neuromorphic hardware). Notably, the task is performed in silico by the connectome rather than in vivo by a person or an animal in the scanner. In silico task performance can then be used as a readout for the reservoir network’s capacity to entertain rich dynamics and, arguably, its ‘computational capacity’ (suitability for performing useful computation) as introduced in refs. ^119–121^. This meaning of ‘computation’ should not be confused with the kind of computation performed by the living brain during cognitive tasks (but see ref. ^122^ for recent evidence of a correlation between the two).

**Fig. 8:**
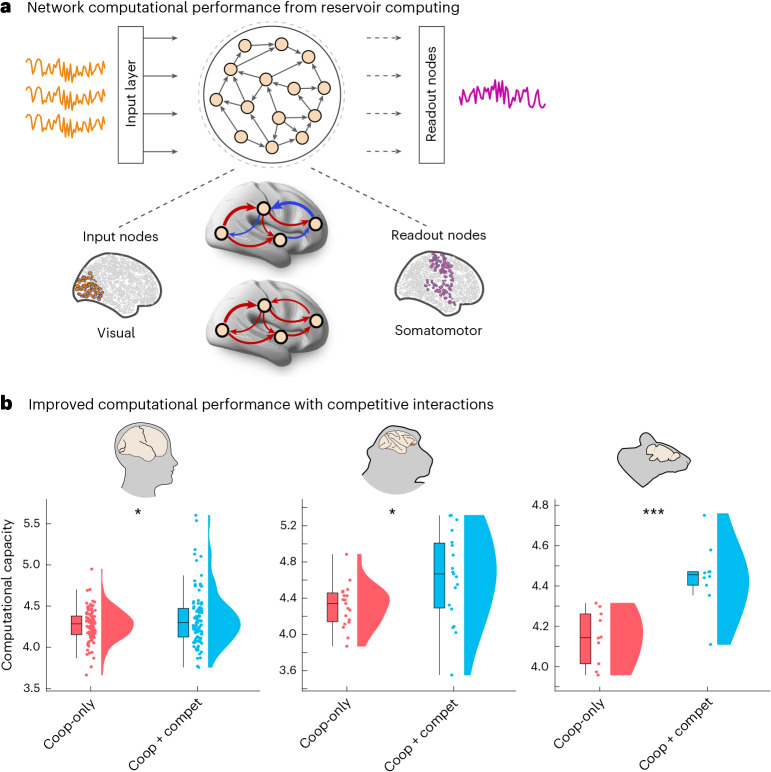
Superior computational performance of connectome-based neuromorphic networks with competitive generative interactions. **a**, The reservoir computing architecture consists of an input layer followed by a reservoir (nonlinear recurrent neural network) and a linear readout module. Here, the wiring between reservoir units is provided by generative brain connectivity patterns, allowing us to evaluate the effect of competitive generative interactions on computational performance using a memory task. In this task, the readout module is trained to reproduce a time-delayed version of a random input signal, measuring the reservoir’s ability to encode past stimuli. We choose visual cortical regions as input nodes and somatomotor regions as output nodes to reflect their respective functional roles. **b**, Memory capacity of the connectome-based reservoir is significantly higher in the presence of negative generative connectivity, across all three mammalian species considered. Human: *n* = 100 individuals; macaque: *n* = 19 data points from 10 animals; mouse: *n* = 10 animals. *P < 0.05 and ***P < 0.001 from paired-sample *t*-tests (two-sided). Box plots: the central lines indicate median values; the bounds of the boxes indicate the 25th and 75th percentiles; and the whiskers indicate 1.5× the interquartile range. Each data point represents one individual. Source data are provided as a Source Data file. Source data

Concretely, the reservoir computing architecture consists of an input layer followed by a reservoir (nonlinear recurrent neural network) and a linear readout module. Here, following the workflow established in refs. ^120,121^, we use the reservoir to perform a memory task. In this task, the readout module is trained to reproduce a time-delayed version of a random input signal, measuring the reservoir’s ability to encode past stimuli. For each species, the size of the reservoir (number of nodes) coincides with the number of regions in that species’s parcellation; input nodes are defined as the visual regions of the cortex, and output nodes are defined as the motor regions, reflecting their biological roles (Fig. 8a). However, we introduce a key distinction from previous work on connectome-based reservoir computing: whereas these previous works used the structural connectome as the wiring diagram between nodes of the reservoir network, here we use instead the generative connectivity matrix produced by our model, which corresponds to a re-weighted and signed version of the input structural connectivity optimized to best reflect the coupling between regions for each individual. Thus, we can now develop reservoir networks that are not only species specific but also individual specific, even when a subject-specific structural connectome is not available.

Notably, our results reveal that the generative connectivity networks obtained from a model with competitive interactions exhibit superior computational capacity, consistently outperforming the networks obtained with cooperative-only interactions (Fig. 8b; human: mean (s.d.) = 4.27 ± 0.19 for cooperative-only; 4.35 ± 0.33 for cooperative–competitive; *t*_99_ = −2.62; *P* = 0.010; Hedge’s *g* = −0.30; macaque: mean (s.d.) = 4.31 ± 0.24 for cooperative-only; 4.63 ± 0.49 for cooperative–competitive; *t*_18_ = −2.62; *P* = 0.017; Hedge’s *g* = −0.81; mouse: mean (s.d.) = 4.15 ± 0.13 for cooperative-only; 4.45 ± 0.16 for cooperative–competitive; *t*_9_ = −7.72; *P* < 0.001; Hedge’s *g* = −1.94). Once again, this effect is observed consistently across species. In other words, in addition to better matching the functional patterns of the human brain, the model with competitive interactions can also be used to execute an actual task with greater performance.

### Robustness and sensitivity

Throughout this work, we consistently replicated our results in three independent datasets pertaining to three distinct mammalian species and different methods of connectome reconstruction, demonstrating their generality. In this section, we summarize the results of several additional control analyses that we performed to ensure the robustness of our findings.

In addition to our alternative fitting criterion of the mutual information between regions (Supplementary Fig. 1), we show that an alternative measure of global functional connectivity fit—the structural similarity index (SSIM), which is commonly used for comparing images—is also significantly improved for the model with both cooperative and competitive interactions in the generative connectivity (Supplementary Fig. 6 and Supplementary Tables 1–3). We also note that the presence of anticorrelations in our three empirical datasets is not due to the preprocessing step of global signal regression (GSR). Indeed, because it is known that this procedure can introduce artificially high levels of anticorrelations in the functional connectivity^5^, we elected not to use GSR for any of our datasets. Therefore, the presence of empirical anticorrelations is unrelated to GSR. Nonetheless, in Supplementary Fig. 7, we show that our main results are successfully replicated when GSR is used, in fact achieving an even better fit between real and simulated human functional connectivity.

We also confirm that the human results can be replicated using a different, more fine-grained parcellation of the cortex (Schaefer-200) and also with the addition of subcortical structures from the Melbourne atlas (Supplementary Fig. 8). Because diffusion tractography is known to have difficulty resolving interhemispheric connections between homotopic regions, we also replicate our human results after explicitly adding homotopic connections, which does result in a small improvement, further demonstrating the value of the structural connectome as biological prior (Supplementary Fig. 9). Similarly, although we symmetrized the macaque and mouse connectomes to facilitate comparison with the human data, we verify that the macaque and mouse results can be replicated if the asymmetries (and, for mouse, the fully dense nature) of the structural connectome are preserved (Supplementary Figs. 10 and 11).

Additionally, we investigate if the results could be driven by a difference in overall connectivity between the resulting cooperative-only and cooperative–competitive models. For this, we consider a different way of comparing these models: rather than fitting a cooperative-only model in the traditional way (whereby updates that would make a connection negative are disallowed), we, instead, obtain the generative connectivity from the cooperative–competitive model and then take the absolute value such that all competitive interactions are now cooperative. As a result, the magnitude of interregional coupling between each pair of regions is identical for the cooperative-only and cooperative–competitive models, which was not guaranteed when fitting the models separately. Nevertheless, results across all three species show that if competitive interactions are turned cooperative, the resulting functional connectivity loses structure and performance declines dramatically, accompanied by a loss of dynamical realism (Supplementary Figs. 12–14). Note that this result should not be surprising: this version of a cooperative-only model could have been produced by the model optimization but was not, because it did not lead to good fit. This analysis confirms that the competitive nature of the interactions is important, not merely their number and weight.

### Role of optimization procedure

Here, we use the same heuristic pseudo-gradient descent optimization as in the latest Hopf modeling literature^43,44^ (that is, the cooperative-only model), following the procedure introduced in ref. ^81^. Therefore, as in these previous works, our model uses the structural connectivity to provide an anatomical constraint on the sparsity of the inferred generative connectivity matrix, because the model is not allowed to update any connections that are not present in the original structural connectivity. This means that the number of parameters to tune coincides with the number of non-zero entries in the structural connectome. Although we do not increase the number of parameters in our model vis-à-vis the cooperative-only model introduced in ref. ^43^, we do increase the possible range of values that generative connections can take, by allowing them to be negative. Thus, we provide several control analyses to demonstrate that an increased range alone is not sufficient to explain the improved performance of the cooperative–competitive model.

**Extended Data Fig. 2:**
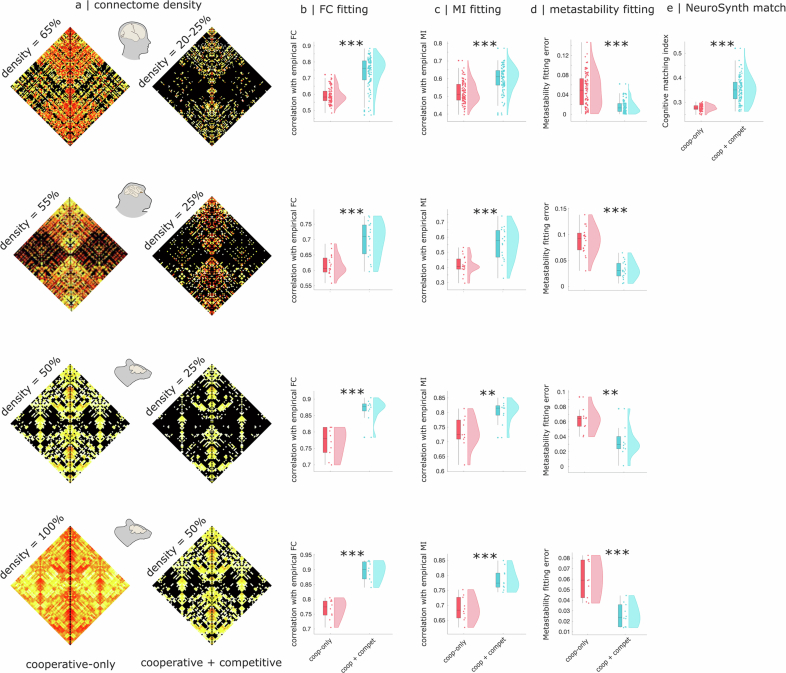
Generative competitive interactions lead to superior model fit over a cooperative-only model with twice as many parameters. Generative competitive interactions lead to superior model fit over a cooperative-only model with twice as many parameters | (**a**) Structural connectomes used for the cooperative-only model (denser, first column) or cooperative-competitive model (sparser, second column). For the human, the denser (~65%) connectome is obtained as a group-average of all individual connectomes. For the macaque, we reduced the density of the SC used for the cooperative-competitive to 25%. For the mouse, two strategies are used: additional thresholding of the SC for the cooperative-competitive model (25% vs 50% density), or using the fully dense SC for the cooperative-only model. (**b**) Correlation between empirical and simulated FC at the level of individual subjects is significantly higher for the model with competitive interactions. Human: *n* = 100 individuals; macaque: *n* = 19 data-points from 10 animals; mouse: *n* = 10 animals. (**c**) Correlation between empirical and simulated mutual information (MI) at the level of individual subjects is significantly higher for the model with competitive interactions. Human: *n* = 100 individuals; macaque: *n* = 19 data-points from 10 animals; mouse: *n* = 10 animals. Mouse 50% vs 25% density: p = 0.005. (**d**) Difference between empirical and simulated metastability is significantly lower for the model with competitive interactions. Human: *n* = 100 individuals; macaque: *n* = 19 data-points from 10 animals; mouse: *n* = 10 animals. Mouse 50% vs 25% density: p = 0.002. (**e**) Cognitive matching from NeuroSynth at the level of individual subjects is significantly higher for the model with competitive interactions. *n* = 100 human individuals. (**b**-**e**) Box plots: the central lines indicate median values, the bounds of the boxes indicate the 25th and 75th percentiles, the whiskers indicate 1.5× the interquartile range. **, p < 0.01; ***, p < 0.001 from paired-samples t-tests (two-sided). Source data are provided as a Source Data file. Source data

First, we double the maximum value allowed for connections in the training of the cooperative-only model (Methods), from 0.1 to 0.2, whereas the cooperative–competitive model is kept at 0.1. Despite the broader range of available values, the cooperative-only model does not approach the level of performance observed for the cooperative–competitive model (Supplementary Fig. 15). Second, we pit the cooperative–competitive model against a cooperative-only model that has twice as many parameters to tune (that is, structural connections that can be adjusted: the structural connectivity density determines the number of parameters in the model, because the model is only allowed to tune a connection if it is non-zero in the starting structural connectivity). This is carried out as follows:

(1) For the human case, we advantage the cooperative-only model: we base it on a group-average connectome (density approximately 65%) instead of the individual connectomes whose density is only approximately 20–25%, corresponding to more than twice as many parameters that the cooperative-only model can tune. (2) For the macaque connectomes, which is already much denser (approximately 55%), instead of advantaging the cooperative-only model, we handicap the cooperative–competitive model by basing it on a thresholded structural connectome that has only 25% density (that is, approximately half the number of parameters). For the mouse connectome, which, for consistency, is thresholded at 50% density in our main analysis but is available as fully dense (see Supplementary Fig. 11 for the cooperative–competitive results with the fully dense mouse model), we adopt both approaches: (3) we compare a cooperative-only model based on 50% density against a cooperative–competitive model based on 25% density; (4) we also compare a cooperative-only mouse model based on 100% density against a cooperative–competitive model based on 50% density. In all cases, even with twice as many degrees of freedom (connections that can be changed), the cooperative-only model is unable to match the performance of the cooperative–competitive model (Extended Data Fig. 2). In other words, allowing competitive interactions allows us to remove half of the model’s parameters compared to the previous (cooperative-only) Hopf model used in recent publications^43,44^ while still achieving superior fit across a wide range of criteria.

A related concern is that high-dimensional optimization problems can present a risk of overfitting, whereby the model is matching idiosyncratic noise in the training data rather than capturing the underlying data-generating process, thereby leading to poor generalization. As one way to mitigate this concern, we implement L1 regularization in our model—a widely used approach to mitigate the risk of overfitting by penalizing large model weights (Methods). We compare the L1-penalized cooperative–competitive model against the original cooperative-only model, and we show that the penalized cooperative–competitive model is still superior, across multiple fitting criteria (Supplementary Fig. 16). This analysis complements the previous approach that explicitly reduced the number of model parameters a priori by using a sparser structural connectivity, suggesting that the superior performance of the cooperative–competitive model vis-à-vis the cooperative-only model is unlikely to be attributable to overfitting.

**Extended Data Fig. 3:**
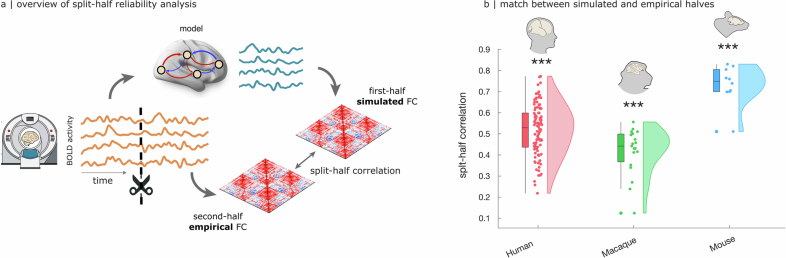
Split-half generalisability of the cooperative-competitive model. Split-half generalisability of the cooperative-competitive model | (**a**) Overview of split-half reliability analysis. We cut the empirical timeseries of each individual in two equal halves; then we fitted a cooperative-competitive model on the first half of the data; and finally, we quantified the correspondence between the model-simulated FC based on the first half of data, and the empirical FC obtained from only the second half of data. (**b**) The FC simulated by the model fitted to the first half of the data is significantly and highly correlated with the empirical FC obtained from the second half of the empirical data, which the model has never seen. ***, p < 0.001 from one-sample t-tests (two-sided) against the null hypothesis of a distribution with zero mean. Human: *n* = 100 individuals; macaque: *n* = 19 data-points from 10 animals; mouse: *n* = 10 animals. Box plots: the central lines indicate median values, the bounds of the boxes indicate the 25th and 75th percentiles, the whiskers indicate 1.5× the interquartile range. Source data are provided as a Source Data file. Source data

**Extended Data Fig. 4:**
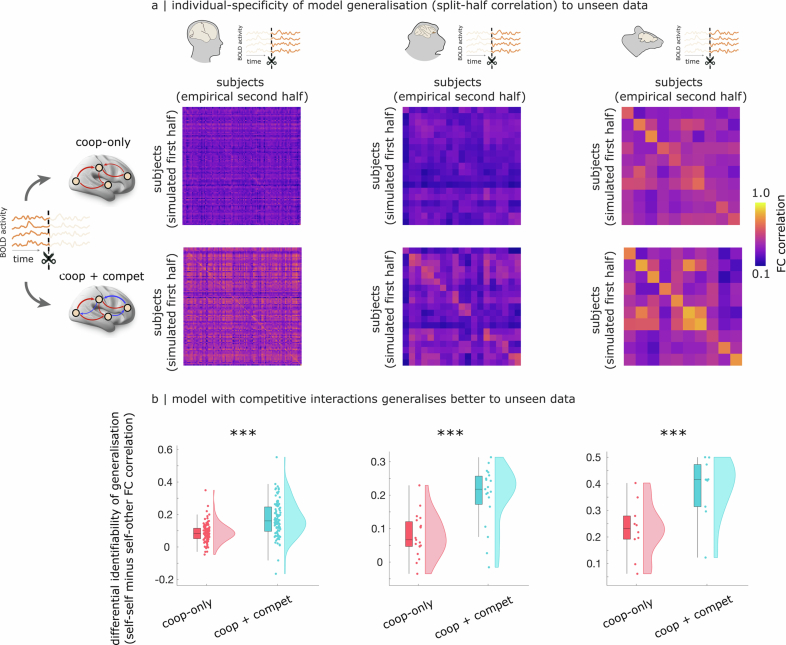
FC simulated from model fitted to the first half of a subject’s data is subject-specific. FC simulated from model fitted to the first half of a subject’s data is subject-specific | (**a**) Identifiability matrices (FC correlation) between each subject’s simulated FC from the first half of data (rows) and empirical FC from the second half of data (columns). Differential identifiability is defined as the difference between self-self similarity of FC (diagonal elements in panel (**a**)) and mean self-other similarity of FC (off-diagonal elements), such that greater distinctiveness of the diagonal implies more individual-specificity. (**b**) Differential identifiability across split-halves is significantly greater than zero, and significantly higher for the cooperative-competitive model than the cooperative-only model. Human: *n* = 100 individuals; macaque: *n* = 19 data-points from 10 animals; mouse: *n* = 10 animals. ***, p < 0.001 from paired-samples t-tests (two-sided). Box plots: the central lines indicate median values, the bounds of the boxes indicate the 25th and 75th percentiles, the whiskers indicate 1.5× the interquartile range. Source data are provided as a Source Data file. Source data

We also take further steps to mitigate the concern of overfitting. First, we perform split-half assessment of our model. We divide the empirical time series in two equal halves; then we optimize the model on the first half; and, finally, we quantify the correspondence between the model-simulated functional connectivity based on the first half of data and the empirical functional connectivity obtained from the second half of data. If the model were overfitting to the noise in the data, it should produce poor fit for any data that it has not explicitly seen before. On the contrary, we show that the model trained on the first half of the empirical fMRI data produces functional connectivity that is highly correlated with the empirical functional connectivity from the unseen second half of the fMRI data (Extended Data Fig. 3) and displays significant subject specificity (Extended Data Fig. 4).

**Extended Data Fig. 5:**
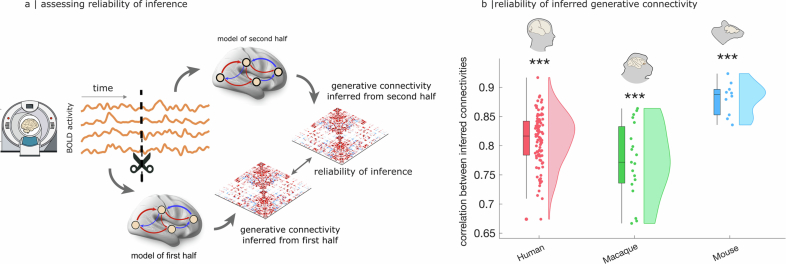
Generative connectivity recovered from different halves of the same empirical fMRI data consistently converge on the same architecture with high accuracy. Generative connectivity recovered from different halves of the same empirical fMRI data consistently converge on the same architecture with high accuracy | (**a**) Overview of split-half reliability analysis. We cut the empirical timeseries of each individual in two equal halves; then we fitted a separate cooperative-competitive model to each half of the data; and finally, we quantified the reliability of the inference as the correspondence between the generative connectivity matrices inferred by the two models. (**b**) The generative connectivity inferred by the two models are significantly and highly correlated, indicating reliable convergence on the same generative architecture. ***, p < 0.001 from one-sample t-tests (two-sided) against the null hypothesis of a distribution with zero mean. Human: *n* = 100 individuals; macaque: *n* = 19 data-points from 10 animals; mouse: *n* = 10 animals. Box plots: the central lines indicate median values, the bounds of the boxes indicate the 25th and 75th percentiles, the whiskers indicate 1.5× the interquartile range. Source data are provided as a Source Data file. Source data

Next, we show that the split-half reliability does not hold only for the simulated functional connectivity but also for the generative connectivity matrices inferred by the model. In each species, generative connectivity matrices obtained by using as input the first and second halves of the same fMRI scan are highly similar (mean correlation >0.75 for macaque and >0.80 for human and mouse; Extended Data Fig. 5). In other words, they consistently converge on the same architecture with high accuracy. This is what should be expected for a model that correctly infers the data-generating process, because the real data were generated by the same biological process. Note that this consistency is all the more noteworthy because such highly similar generative connectivity matrices are not obtained from two repetitions performed on the same input data but, rather, on different halves of the data.

Conversely, we reasoned that if the model is reflecting a biological data-generating process, then its performance should be compromised when trying to fit data that are not biological. If, instead, the model is overfitting, then its performance should be just as high on biological and non-biological data. To test this, we use a circular-shifted surrogate model, whereby the time series are shifted by a random number of timepoints, thereby preserving univariate time series properties (mean, amplitude, autocorrelation spectrum, etc.) because the signals are exactly the same, but the cross-correlation is reduced to chance levels^50,84^. We show that our model is significantly better at reproducing the real biological data than the circular-shifted null data (Supplementary Fig. 17). This superior ability for reproducing biological versus random data should not be observed in a model that is overfitting.

### Validation and stability of inferred generative connections

**Extended Data Fig. 6:**
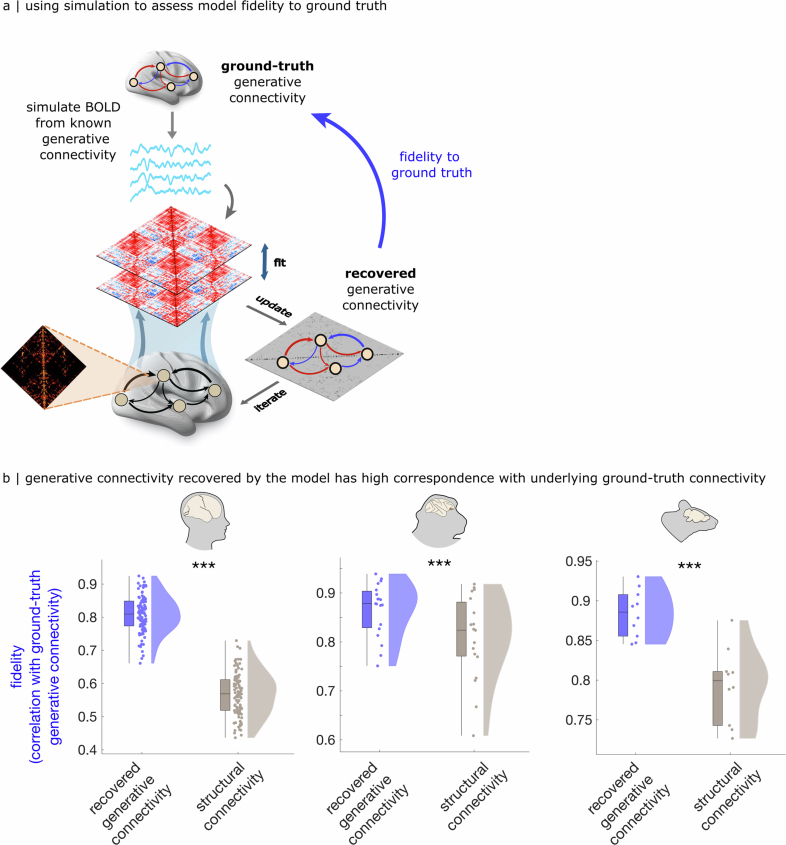
Cooperative-competitive model systematically recovers ground-truth generative connectivity. Cooperative-competitive model systematically recovers ground-truth generative connectivity | (**a**) We used a Hopf model to simulate time-series based on a generative connectivity matrix with both cooperative and competitive connections (one per subject). Then we used a L1-regularised cooperative-competitive model to fit the data, that is recover the generative connectivity, and we quantified the correlation between the ground-truth and inferred generative connectivity. (**b**) The inferred generative connectivity displays high fidelity to the ground-truth generative connectivity, across individuals and across species, significantly outperforming the structural connectivity. ***, p < 0.001 from paired-samples t-tests (two-sided). Human: *n* = 100 individuals; macaque: *n* = 19 data-points from 10 animals; mouse: *n* = 10 animals. Box plots: the central lines indicate median values, the bounds of the boxes indicate the 25th and 75th percentiles, the whiskers indicate 1.5× the interquartile range. Source data are provided as a Source Data file. Source data

Another related concern is degeneracy: for high-dimensional optimization problems, different combinations of parameters (here, corresponding to different configurations of the generative connectivity) could provide equivalent performance. In other words, the final model output may reflect one of many possible local minima rather than the true generative connectivity that gave rise to the input time series. To address this concern, we demonstrate that the model can faithfully recover the ground truth generative connectivity. Although, of course, this is not known for the empirical data, we can use the generative connectivity of each subject (obtained from our model) to generate simulated blood oxygen level-dependent (BOLD) time series. For these time series, we, therefore, have the ground truth of the underlying generative connectivity. Then, we provide the simulated time series as inputs to our model (Extended Data Fig. 6a), asking: Can our model recover the known ground truth?

We find that the answer is unequivocally yes (Extended Data Fig. 6b). The subjectwise correlations between ground truth generative connectivity (the one used to simulate the time series) and recovered generative connectivity (the one inferred by our model, starting from the simulated timeseries) is approximately 0.85 or more for most individuals. This is significantly higher than the correlation between the recovered generative connectivity and the structural connectivity used for initialization, indicating that the model-based inference is providing valuable information. We also observe correlations of approximately 0.95 between the prevalence of negative connections in the ground truth generative connectivity and the recovered generative connectivity (Supplementary Fig. 18). These results provide confidence that the model is consistently converging toward a generative connectivity that is close to the ground truth. In other words, even if the final solution identified by the model is a local minimum, we demonstrate that such local minima are close to the ground truth.

**Extended Data Fig. 7:**
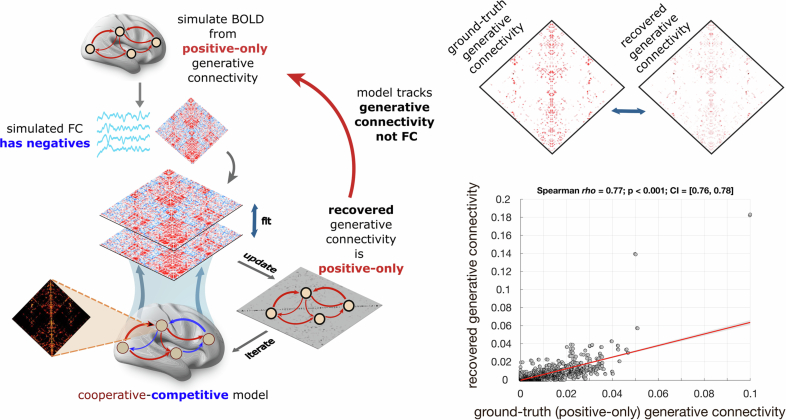
Cooperative-competitive model can recover positive-only ground-truth weights in the presence of negative FC. Cooperative-competitive model can recover positive-only ground-truth weights in the presence of negative FC | (Left) We used a cooperative-only Hopf model to simulate time-series whose functional connectivity included ~30% of negative values. Then we used the L1-regularised cooperative-competitive model to fit the data, that is recover the generative connectivity. (Right) Despite the presence of negative FC, the recovered generative connectivity features no negative values, and displays high fidelity to the true generative connectivity that generated the input data (Spearman’s rho = 0.77, p < 0.001, two-sided). Shading indicates 95% confidence intervals (CI). Source data are provided as a Source Data file. Source data

We further confirm that if the ground truth generative connectivity does not include negative generative connections, then the cooperative–competitive model is likely to produce a positive-only generative connectivity matrix, even in the presence of negative entries in the functional connectivity. To show this, we start from a cooperative-only generative coupling matrix, which represents our ground truth. Next, we use it to simulate time series whose functional connectivity includes approximately 30% negative values. Finally, we use a cooperative–competitive model to fit the data—that is, recover the generative connectivity, using the simulated time series instead of empirical BOLD. We find that the recovered generative connectivity is indeed achieved without negative values and with high fidelity to the ground truth coupling matrix that generated the input data (Extended Data Fig. 7). Thus, we show that our model can faithfully track the presence or absence of negative weights in the generative connectivity.

Taken together, these analyses indicate that the model converges on stable and reproducible configurations. Furthermore, in the case where the ground truth is known, we have clear evidence that such stable configurations consistently and with high accuracy are located in the vicinity of the ground truth connectivity that generated its input time series. On the one hand, this suggests that when competitive connections truly are present, the model will be able to detect them; and, on the other hand, it is evidence that when our model identifies negative connections, they are likely to be indicative of true competitive interactions in the generative connectivity.

### Role of anatomical connectivity as biological prior

**Extended Data Fig. 8:**
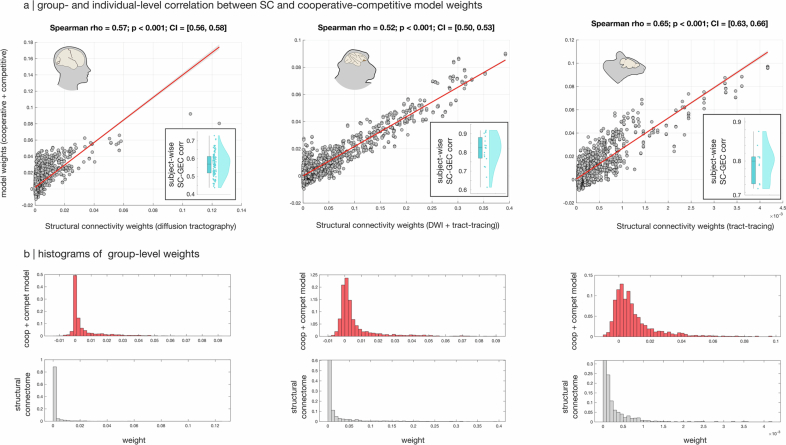
Similarity between generative connectivity inferred using cooperative-competitive model, and anatomical connectivity across species. Similarity between generative connectivity inferred using cooperative-competitive model, and anatomical connectivity across species | (**a**) Scatterplots indicate significant Spearman correlation (two-sided) between group-level generative connectivity inferred from the cooperative-competitive model, and structural connectivity. Shading indicates 95% confidence intervals (CI). Insets: violin plots of individual-level correlation between SC and generative connectivity. At both group-level and individual-level, stronger SC edges translate to stronger edges in the generative connectivity. Human: *n* = 100 individuals; macaque: *n* = 19 data-points from 10 animals; mouse: *n* = 10 animals. Box plots: the central lines indicate median values, the bounds of the boxes indicate the 25th and 75th percentiles, the whiskers indicate 1.5× the interquartile range. (**b**) Histograms of weights for the model-inferred generative connectivity, and the anatomical connectivity, revealing distributions with a pronounced right tail in all three species (human: diffusion tractography; macaque and mouse: tract-tracing). Source data are provided as a Source Data file. Source data

Our model fitting uses the structural connectome as a source of anatomical constraint on the sparsity and location of connections: only connections that are present in the structural connectivity can be tuned by the model^43,81^. For the connections that do exist, the structural connectivity also acts as a biological prior for the final weight, because the model is initialized from the original structural connectivity weight. Indeed, we find that this biological information is preserved in the final model: there is high and significant correlation between the final generative connectivity matrix and the original structural connectivity on which it is based (whether obtained from diffusion tractography in humans or from tract tracing in animals), at both the group level and the individual subject level (human mean *ρ* = 0.57, macaque mean *ρ* = 0.81, mouse mean *ρ* = 0.79; Extended Data Fig. 8). This is evidence that the structural connectivity is successfully acting as a prior for the model: stronger structural connectivity edges tend to translate to stronger generative edges.

Next, we deploy several null models (Methods) to demonstrate more directly that both the location and weight of the empirical structural connectivity contribute to the fit of the model to biological data. Each of these null models is optimized following the same procedure as the main cooperative–competitive model, thereby disentangling the improved performance due to presence of competitive interactions from the role of weight heterogeneity and network topology of the connectome. Concretely, we find that model fit is broadly degraded, across criteria and across species: (1) if the model is initialized using a binarized connectome, thereby removing biological information about heterogeneity of weights (Supplementary Fig. 19), or (2) if the model is initialized from a network where existing connections have been replaced with randomly placed non-existing ones of equal weight, whether or not the degree sequence is also preserved, thereby demonstrating the importance of network topology (Supplementary Figs. 20 and 21). Notably, these analyses also reveal that the benefit in metastability fit provided by the presence of negative generative weights is largely independent of their specific placement but only on node degree (number of connections): once degree is accounted for, equivalent levels of fit are observed for real or random topology (Supplementary Figs. 20 and 21). By contrast, the functional connectivity/mutual information and NeuroSynth fitting criteria (based on interregional interactions and instantaneous activation patterns, respectively) appear to be more sensitive to the topology of the anatomical network used for initialization, such that starting from a randomized structural connectivity degrades model performance vis-à-vis the model based on real connectomes (Supplementary Figs. 20 and 21).

Similar results are observed for the memory capacity obtained from using the model-derived generative connectivity as the wiring diagram for a reservoir computing network (Supplementary Fig. 22). Namely, a reservoir based on the generative connectivity from the cooperative–competitive model broadly outperforms reservoirs based on the generative connectivity from (1) the cooperative-only model with twice the number of connections (except in the case of the macaque where the denser connectome prevails); (2) cooperative–competitive models initialized from null structural connectomes that preserve the topology but obliterate weight differences (binary); (3) cooperative–competitive models initialized from null structural connectivity with preserved weight distribution but randomized topology (that is, replacing existing connections with non-existing ones of equal weight); and (4) cooperative–competitive models initialized from null structural connectivity with preserved weight distribution and degree sequence (Supplementary Fig. 22). In other words, a cooperative–competitive generative matrix initialized from real biological connectivity provides the best basis for a high-performing reservoir network.

Altogether, the model (1) reproduces unoptimized dynamics of the data (synergy, metastability, hierarchy and co-activation of cognitive patterns); (2) is unable to match biologically implausible null data; but (3) successfully generalizes to unseen real data from the same individual. The model-inferred connections are highly stable and subject specific and can closely match a known ground truth generative connectivity. Performance is consistently degraded when the input time series or input connectome are replaced with biologically implausible surrogates. These are all hallmarks of a model that is capturing the true data-generating process rather than overfitting to the idiosyncratic noise in the training data.

## Discussion

Altogether, we examined the dynamical and computational relevance of competitive interactions in the mammalian connectome. We used generative computational modeling to integrate multimodal data about brain structure and function across human, macaque and mouse brains. Consistently across species, our best generative account of how brain network structure gives rise to function combines modular cooperative interactions with diffuse, long-range competitive interactions between regions with opposite biological annotations. Generative models with competitive interactions are more individual specific and achieve excellent fidelity to the spatial coordination and temporal dynamics of the empirical brain, including properties that were not explicitly optimized but, rather, emerged spontaneously. Below, we discuss the implications of these findings while also positioning our model in the broader space of possible models in the literature and outlining promising directions for future work.

Our species-specific computational models revealed that allowing the generative connectivity to include competitive as well as cooperative interactions produces consistently superior fit to the empirical functional connectivity, at both the group level (with up to 0.95 correlation between real and simulated functional connectivity) and the level of individual subjects. Notably, upon introducing competitive interactions, the increase in the model’s explicit fitting objective (spatial organization of functional connectivity) was accompanied by increased fit to several additional, unoptimized properties, which are dynamical rather than spatial: metastability, synergy and local–global hierarchical organization. We found that our signature of metastability in the model with cooperative-only interactions is consistently higher than in real brains (consistent with similar observations from the recent voxelwise model of ref. ^33^). Adding competitive interactions brings it closer to empirically observed levels. A possible interpretation of this observation is that metastability (here, operationalized as the temporal variability of the KOP, as is common in the fMRI literature^73,105,106^) is intended to quantify a balance between integrative and segregative tendencies. Temporal variability of the brain’s level of synchrony (KOP) indicates that high and low synchronization co-exist and alternate over time. If only cooperative interactions are present, this can lead to periods of excessive synchrony due to regions reciprocally increasing each other’s activity level in a vicious cycle (we confirm this in Supplementary Fig. 23, showing that the maximum observed synchrony is significantly higher for the model without competitive interactions, often approaching total synchronization). Competitive interactions may play a stabilizing role, because as one region’s activity grows, activity of its competitively connected neighbors will diminish, preventing runaway global activity.

Finally, synergy reflects the presence of complementary information in different brain regions’ activity, such that there is greater ability to predict the joint future state of two brain regions when both regions are considered together rather than in isolation^110,111^. In practice, synergy is low both when two regions are fully independent (such that neither of them has information about the other but only about itself) but also when two regions are strongly dependent, such that the same information is present in each, without need to consider the other^110,111^. Thus, synergy reflects a balance between dependency and independence in the system, complementing metastability (which, in its operationalization as std(KOP), considers only instantaneous relationships). A system with synergy is a system whose elements act as a whole rather than as disjoint parts. We found that by countering the tendency to extreme synchronization, competitive interactions allow greater diversity of activity to be present and available for integration, manifesting as greater synergy.

Altogether, competitive interactions increase synergy and hierarchical functional organization while exerting a moderating effect on metastability, bringing the simulated dynamics more in line with empirical dynamics of the mammalian brain. This increased fidelity of the model’s dynamics is accompanied by increased realism of the specific patterns of model-generated brain activity, as revealed by our recently introduced ‘cognitive matching’ criterion. The cognitive matching index reflects the similarity between spontaneous brain activity and brain patterns associated with 123 canonical cognitive operations, as synthesized in a data-driven way from over 14,000 published neuroimaging studies^103^. When applied to recordings from real human brains, cognitive matching may be understood as recovering echoes of cognition in the spontaneous activity of the brain. When translated into a criterion of model realism, as introduced in the present report, cognitive matching measures whether the model co-activates regions in a manner consistent with their membership of the same cognitive circuits. Thus, the present results suggest that competitive interactions are crucial for enabling co-activation of regions that belong to the same cognitive macro-circuit, because we are considerably less likely to observe appropriate co-activations in the absence of competitive interactions.

A key advantage of our model-based approach is that, unlike studies of functional connectivity, here we can assign a definite meaning to the negative sign of the competitive generative interactions: they represent suppression of the target’s activity by the source, justifying the term ‘competitive’. However, we highlight the caveat that a competitive interaction in our model need not correspond to a long-range inhibitory connection. Suppressive influences of one region over another could arise from a variety of biological mechanisms, potentially including direct long-range inhibitory projections but also (and perhaps more likely) long-range excitatory projections onto local inhibitory circuits, or homeostatic regulation, network-level feedback, or even more complex phenomena such as conduction delays and the relative contributions of cerebral blood volume and flow to the hemodynamic signal, among others^5,88–92^. Indeed, many such biological mechanisms could even co-exist, implementing competitive interactions in different parts of the brain or in different species (see Supplementary Note 2 for a more in-depth discussion of this point and of the role of computational modeling for biological discovery).

Altogether, our generative model consistently indicates that competitive interactions play a pivotal role in explaining the emergence of functional interactions from the brain’s structural connectivity, achieving unprecedented realism. This meaning of ‘functional’ goes beyond regional co-fluctuations (what is commonly referred to as functional connectivity), instead also encompassing co-activation of brain regions in accordance with membership of the same cognitive macro-circuit, as determined by meta-analytic synthesis with NeuroSynth. The same competitive interactions also endow brain dynamics with greater synergy and hierarchical organization and overall greater fidelity to the empirical dynamics. Note that only functional connectivity and lagged functional connectivity are explicitly optimized by the algorithm, yet the remaining criteria are also significantly improved. Along with the model’s excellent split-half reliability, successful recovery of the ground truth connectivity, and degraded performance upon replacing the biological input data with null surrogates, this evidence suggests that the model is not overfitting to idiosyncratic noise in the input data but, rather, genuinely converging toward the data-generating process across both spatial and dynamical dimensions.

Systematic patterns can also be identified in the organization of competitive interactions, which are consistent across species. Whereas cooperative (positive-valued) interactions in the generative connectivity tend to be strong, modular and relatively short range, competitive interactions are weaker but more long range, more diffuse and less clustered: it is less likely that competitively interacting neighbors of a node will themselves be interacting competitively. Corroborating these results, lower clustering for negatively weighted connections than for positively weighted connections was also recently reported by Tanner et al.^50^. Crucially, we found that competitive generative connections consistently link regions characterized by opposite (anticorrelated) patterns of biological annotations and situated at opposite ends of the cortical hierarchy. This observation was highly consistent across all three species and across multiple modalities of biological organization, including cytoarchitecture, microstructural profile, myeloarchitecture, gene expression and receptor expression. It stands to reason that if macroscale competition exists in the brain, it should preferentially occur between regions with opposite biological profiles. For example, if two regions express opposite patterns of receptors, then they are likely to respond in opposite ways to the same input signal. The consistency of these observations across species and modalities, therefore, hints at possible phylogenetically conserved biological origins for the competitive interactions observed here, grounded in systematic patterns of regional heterogeneity in the mammalian cortex.

The phenomenon of strong modular connections complemented by diffuse weaker ones is reminiscent of the ‘strength of weak ties’, observed in social and neuronal networks alike^123^. Our present results suggest that, in the mammalian brain, many such weak ties may be of a predominantly competitive nature. The resulting architecture balances local cooperation (within specialized modules) and global competition, reminiscent of the ‘global workspace’ architecture^2,4^. We have shown that such a cooperative–competitive architecture achieves a greater range of local-to-global responses and greater synergy in its dynamics. In the real human brain, synergy is especially prevalent in integrative regions of association cortex that support higher-order cognitive functions^110,111,124^.

Convergent evidence for an association between synergy and cognitive capacity was recently provided, showing that synergy supports flexible and efficient learning in artificial neural networks^125^. This evidence is further corroborated by our own present results: across humans, macaques and mice, we found that connectome-based neuromorphic networks reach the highest performance on a memory task (recently introduced as a measure of in silico ‘computational capacity’^119–122^) when both cooperative and competitive interactions are present, especially when connection placement and heterogeneity respect empirical biological organization. It is intriguing that, in addition to matching spatiotemporal properties and dynamical richness of the real brain, competitive interactions also endow the generative connectivity with greater computational capacity to support memory processes—arguably a key building block of cognition. To be clear: there are countless possible types of ‘computation’, and our artificial measure of computation (memory capacity) is qualitatively different and more abstract than the kind of computation performed by the living brain during cognitive tasks. Cognitive tasks can themselves be modeled at multiple levels of abstraction. At one end are attractor and drift-diffusion models. At the other end are detailed biophysical models that incorporate anatomical constraints such as cell type composition or NMDA, AMPA and dopamine receptors, including recent work showing that biologically plausible gradients of feedforward excitation and feedback inhibition between different cell types contribute to more realistic spatiotemporal patterns of propagation and persistence of sensory information (that is, stimulus detection and working memory)^28,126–128^. Our connectome-based reservoir using generative connectivity arguably lies in between these two extremes. Notably, a recent study showed that, across hundreds of human participants, computational memory capacity estimated from structural connectome-based reservoir computing is associated with cognitive performance in several domains, notably including memory itself^122^. Altogether, connectome-based reservoir computing provides a valuable complement for existing methods of assessing the functional relevance of macroscale brain networks^120,121^, which we leverage here to provide convergent evidence about the dynamical consequences of competitive interactions.

Altogether, the present work provides a generative link between network architecture and dynamical properties in the mammalian brain, expanding understanding of how structure can give rise to function. At the same time, this work represents a major advance in the development of in silico brain models—a topic of intense research^41^. Generative models such as ours are particularly desirable from a clinical perspective, to develop virtual screening tools for potential interventions^41^. Compared with state-of-the-art cooperative-only models of this kind^43,44^, our cooperative–competitive model achieves markedly superior fidelity across diverse dimensions of brain connectivity and dynamics. This is observed not only at the group level: our models are fitted to individual human brains using their own structural and functional data. In addition to providing a superior match to the empirical brain of each individual, our improved model exhibits also greater subject specificity. In other words, we do not simply have a better model of human brain function; we have a better model of the brain of each individual. This represents a critical advance toward the development of personalized computational models of individual patients’ brains, based on their own data. The success of our modeling framework is not limited to the human brain, but, rather, it extends to mouse and macaque, highlighting the similarities between humans and two fundamental model organisms in neuroscience. Invasive therapeutic interventions are typically assessed in animal models prior to human trials^15^, adding translational potential to our working model of the mammalian brain.

## Methods

See Supplementary Methods for species-specific details of fMRI data acquisition and processing; species-specific structural connectome reconstruction; and species-specific gene expression, receptor density, cell type composition, myeloarchitecture and other biological annotations.

### Generative whole-brain modeling

The local dynamics of each individual node is described by the normal form of a supercritical Hopf bifurcation with stochastic input, which is able to describe the transition from asynchronous noisy behavior to full oscillations.

Oscillators have been widely used to model many physical systems, going from the simplest linear, harmonic oscillator to nonlinear oscillators^129,130^. Small perturbations to linear oscillators lead to changes in oscillation amplitudes, whereas perturbations to nonlinear oscillators lead to self-regulating relaxation and a return to the same region in phase space. With an ordinary differential equation of a complex order parameter, the Stuart–Landau model of a single oscillator provides the simplest nonlinear extension of a linear oscillator that mathematically describes the onset of spontaneous oscillations (that is, bifurcation from fixed-point dynamics toward a limit cycle)^130^. Specifically, in the regime at the edge (but just below) the Hopf bifurcation, the Hopf model generates neither mere noise nor the single sustained oscillation of Wilson–Cowan and Kuramoto models but, rather, a fluctuating stochastically structured signal with oscillatory components that matches the infra-slow fluctuations typically observed in fMRI signal^74–76,131^.

Thus, each node $n$ is represented by the following set of coupled stochastic differential equations in Cartesian coordinates:

$$\frac{{\mathrm{d}}{x}_{n}}{{\mathrm{d}}{t}}=({a}_{n}-{x}_{n}^{2}-{y}_{n}^{2}){x}_{n}-{\omega }_{n}{y}_{n}{+\beta \eta }_{n}(t)$$

1 $$\frac{{\mathrm{d}}{y}_{n}}{{\mathrm{d}}{t}}=\left({a}_{n}-{x}_{n}^{2}-{y}_{n}^{2}\right){y}_{n}+{\omega }_{n}{x}_{n}{+\beta \eta }_{n}(t)$$

In these equations, $\eta$ represents additive Gaussian noise with a standard deviation $\beta$. The system undergoes a supercritical bifurcation at ${a}_{n}=0$. When ${a}_{n} > 0$, the system engages in a stable limit cycle with a frequency ${f}_{n}=\frac{{\omega }_{n}}{2\pi }$, whereas, for ${a}_{n} < 0$, the dynamics stabilize at a fixed point, representing a low-activity noisy state dominated by the Gaussian noise. In this model, each node has an intrinsic frequency ${\omega }_{n}$ within the range of the empirical fMRI range, determined by the averaged peak frequency of the narrowband BOLD signals in each brain region. We set ${a}_{n}$ = −0.02, consistent with previous studies^43,44^.

To model whole-brain dynamics, we incorporate coupling between regions using a diffusive coupling term. This term represents the input received by region *n* from every other region *p*, weighted by the corresponding connection from the adjacency matrix ${G}_{{np}}$, representing empirical structural connectivity. The input is modeled using a common difference coupling, approximating the simplest (linear) component of a general coupling function. The equations governing the whole-brain dynamics are as follows:

$$\frac{{\mathrm{d}}{x}_{n}}{{\mathrm{d}}{t}}=({a}_{n}-{x}_{n}^{2}-{y}_{n}^{2}){x}_{n}-{\omega }_{n}{y}_{n}+\mathop{\sum }\limits_{p=1}^{N}{G}_{{np}}({x}_{p}{-x}_{n}){+\beta \eta }_{n}(t)$$

2 $$\frac{{\mathrm{d}}{y}_{n}}{{\mathrm{d}}{t}}=\left({a}_{n}-{x}_{n}^{2}-{y}_{n}^{2}\right){y}_{n}+{\omega }_{n}{x}_{n}+\mathop{\sum }\limits_{p=1}^{N}{G}_{{np}}(\,{y}_{p}{-y}_{n}){+\beta \eta }_{n}(t)$$

In these equations, the noise standard deviation is set to $\beta$ = 0.01. This coupled oscillator model effectively captures the transition between different dynamical states of the brain and provides a framework for understanding how brain regions interact to produce complex patterns of activity.

### Updating the generative connectivity

We optimized the generative connectivity (GC) between brain regions by aligning the model’s output with empirical measures, specifically forward and reverse time-shifted correlations and empirical functional connectivity (FC). The same heuristic gradient algorithm previously introduced in the Hopf modeling literature^43,44^ was then employed to iteratively update the GC, refining the fit:

3 $${\mathrm{GC}}_{{np}}={\mathrm{GC}}_{{np}}+\varepsilon \left({\mathrm{FC}}_{np}^{\,{\mathrm{emp}}}-{\mathrm{FC}}_{{np}}^{{\ }{\mathrm{sim}}}\right)+\left({\mathrm{FC}}_{{\mathrm{lag}},np}^{\,{\mathrm{emp}}}-{\mathrm{FC}}_{{\mathrm{lag}},np}^{\,{\mathrm{emp}}}\right)$$

The model was iteratively run with the updated generative connectivity until the fit converged to a stable value. As per previous work, the maximum weight is capped at a value of 0.1. However, for one of our control analyses, the maximum weight of the cooperative-only model was, instead, allowed to reach a value of 0.2.

Crucially, the algorithm does not tune all possible connections between regions. Rather, following the current Hopf modeling literature^43,44^, and in line with recent developments more broadly^44,81^, initialization is based on empirical anatomical connectivity, and the model is only allowed to update non-zero connections within the matrix, which, therefore, provides a biological constraint on the sparsity of the model-inferred generative connectivity. The structural connectivity also provides a biological prior on the weight of the generative connectivity, because model weights are initialized from the structural connectivity weights^43^. Following previous work^43^, the algorithm was run with *ε* = 0.0002 and *ε*′ = 0.00004, continuing until convergence was achieved. Two versions of the algorithm were considered: cooperative-only, whereby connection updates that reached negative values were not allowed (note that this is not a new type of model introduced here but, rather, matches the current literature on Hopf modeling^43,44^) and cooperative–competitive, whereby the algorithm is allowed to update the connections to negative values. In both cases, however, the model is only allowed to update connections that are present in the structural connectivity matrix provided at initialization.

We also implemented an optional step of L1 regularization. L1 regularization is applied to promote sparsity in the estimated generative connectivity matrix, by adding a penalty term proportional to the absolute magnitude of each connection weight. Specifically, during each optimization iteration, the term α_L1_ × sign(GEC(*i*,*j*)) is subtracted from the connection update. Here, α_L1_ represents the regularization strength, and the sign function provides the gradient of the L1 penalty term with respect to the connection weight. This regularization technique encourages the optimization algorithm to drive weak or redundant connections toward zero, thereby identifying a relevant subset of the most functionally relevant effective connections while maintaining the model’s ability to reproduce empirical functional connectivity patterns.

### Differential identifiability for model evaluation

‘Brain fingerprinting’ refers to using brain-derived metrics (here, the functional connectivity obtained from resting-state fMRI) to discriminate individuals from each other, analogously to how the grooves on one’s fingertips may be used to discern one’s identity^102,104^. This requires brain fingerprints (just like conventional fingerprints) to be different across different people (to avoid confusing distinct individuals) but consistent within the same individual (to track identity).

Let *A* be the ‘identifiability matrix’—that is, the similarity between individuals’ test and retest scans (or, in this case, individuals’ empirical and simulated functional connectivity), such that the size of *A* is *S* × *S* (with *S* being the number of individuals in the dataset). Each entry of *A* is obtained as the correlation between the corresponding individuals’ vectorized matrices of functional connectivity. Let *I*_self_ = 〈*A*_*ii*_〉 represent the average of the main diagonal elements of *A*, which consist of the Pearson’s correlation values between scans of the same individual: from now on, we will refer to this quantity as self-identifiability or *I*_self_. Similarly, let *I*_others_ = 〈*A*_*ij*_〉 define the average of the off-diagonal elements of matrix *A*—that is, the correlation between models and scans of different individuals *i* and *j*. Then, we define the differential identifiability (*I*_diff_) of the sample as the difference between both terms^102^:

4 $${I}_{\mathrm{diff}}=({I}_{\mathrm{self}}-{I}_{\mathrm{others}})$$

which quantifies the difference between the average functional connectivityʼs similarity using the matched model and the average functional connectivityʼs similarity when model and empirical data are mismatched. The higher the value of *I*_diff_, the higher the model’s individual specificity^102,103^.

### Dynamical measures

#### Synchrony and metastability

Metastability was quantified using a widely used signature: the standard deviation of the KOP across time (std(KOP))^73,105,106^.

In turn, the KOP is defined by the following equation:

5 $${\mathrm{KOP}}_{t}=|\mathop{\sum }\limits_{k=1}^{n}{e}^{i{\phi }_{k}(t)}|/n$$

where *φ*_*k*_(*t*) is the instantaneous phase of each bandpass-filtered BOLD signal at node *k*.

Following ref. ^73^: ‘We computed the instantaneous phase *φ*_*k*_(*t*) of each bandpass-filtered signal *k* using the Hilbert transform. The Hilbert transform yields the associated analytical signals. The analytic signal represents a narrowband signal, s(t), in the time domain as a rotating vector with an instantaneous phase, *φ*(*t*), and an instantaneous amplitude, *A*(*t*). Thus*, s*($t$) = $A$ ($t$)cos($\phi$($t$)). The phase and the amplitude are given by the argument and the modulus, respectively, of the complex signal *z*(*t*), given by $z$($t$)=$s$($t$)+${iH}$[$s$($t$)], where *i* is the imaginary unit and *H*[*s*(*t*)] is the Hilbert transform of *s*(t)’. At each point in time, the KOP measures the global level of synchronization across these oscillating signals. Under complete independence, the phases are uniformly distributed, and, thus, KOP is nearly zero, whereas KOP = 1 if all phases are equal (full synchronization)^132^.

The variability (standard deviation) of the KOP over time is commonly used as a signature of metastability, first proposed by Shanahan et al.^105^ and also adopted by others^106,133^. See ref. ^106^ for a detailed discussion of the difference between metastability and its various signatures. Intuitively, if std(KOP) is high, it indicates that the system alternates between high and low synchronization, thereby combining tendencies for integration (high synchrony) and segregation (low synchrony). For each individual (and simulation), we obtain the std(KOP) signature of metastability as well as the maximum observed value of global synchrony.

#### Local–global hierarchy from intrinsic-driven ignition

Intrinsic-driven ignition (IDI)^108^ quantifies the extent to which spontaneously occurring (‘intrinsic’) neural events elicit activation across the rest of the brain (‘ignition’). The fMRI signal time series are transformed into *z*-scores and subsequently thresholded to obtain a binary sequence *σ* based on the combined mean and standard deviation of the regional transformed signal, such that *σ*(*t*) = 1 if *z*(*t*) > 1 and is crossing the threshold from below, indicating that a local event has been triggered; otherwise, *σ*(*t*) = 0. Subsequently, for each brain region, when that region triggers a local event (*σ*(*t*) = 1), the resulting global ignition is computed within a time window of 4 time-points (note that the threshold of 1 s.d. and window of 4 time-points for defining an event are chosen for consistency with previous work^34,108^, but it has been demonstrated that the results of this procedure are robust to the specific threshold chosen^134^).

An *N* × *N* binary matrix *M* is then constructed, indicating whether, in the period of time under consideration, two regions *i* and *j* both triggered an event (*M*_*ij*_ = 1). The size of the largest connected component of this binary matrix *M* defines the breadth of the global ignition generated by the driver region at time *t*, termed IDI. To obtain a measure of local–global hierarchy, the variability (standard deviation) across event sizes is then computed^34,108^. Consequently, higher standard deviation reflects more heterogeneity with respect to regions’ capability to induce ignition, which suggests, in turn, a more elaborate hierarchical organization between them.

#### Hierarchy from temporal irreversibility

We estimate pairwise interactions between brain regions by computing time-shifted correlations between both the forward and the reversed fMRI BOLD time series of any two regions^43,109^. This method effectively quantifies the asymmetry in interactions between region pairs, thereby indicating how one region influences another. This approach is inspired by thermodynamics, where the breaking of detailed balance is associated with non-reversibility, often referred to as the ‘arrow of time’. Irreversibility is captured as the difference between the time-shifted correlations of forward and reverse time series^43,109^.

To illustrate, consider the detection of irreversibility between two time series, *x*(*t*) and *y*(*t*). The causal dependency between *x*(*t*) and *y*(*t*) is measured using time-shifted correlations. For forward evolution, the time-shifted correlation is given by:

6 $${c}_{\mathrm{forward}}(\Delta t)= < x(t),y(t+\Delta t) >$$

Similarly, we create a reversed version of *x*(*t*) (or *y*(*t*)), denoted ${x}^{(r)}(t)$ (or ${y}^{(r)}(t)$), by inverting the time sequence. The time-shifted correlation for the reversed evolution is then:

7 $${c}_{\mathrm{reversed}}(\Delta t)= < {x}^{(r)}(t),{y}^{(r)}(t+\Delta t) >$$

The pairwise level of irreversibility, representing the degree of temporal asymmetry or the arrow of time, is quantified as the absolute difference between the forward and reversed time-shifted correlations at a given shift Δ*t* = *T* (here, for consistency across species, we set *T* = 1 TR):

8 $${I}_{x,y}(T)={|c}_{\mathrm{forward}}(T)-{c}_{\mathrm{reversed}}(T)|$$

To compute the whole-brain level of non-reversibility, we defined forward and reversal matrices of time-shifted correlations for the forward version ${x}_{n}(t)$ and the reversed backward version ${x}_{n}^{(r)}(t)$ of a multidimensional time series, where the subscript *n* represents different brain regions. These matrices capture the functional causal dependencies between the variables in the forward and artificially generated reversed time series, respectively. The forward and reversed matrices are expressed as:

$${\mathrm{FS}}_{{\mathrm{forward}}{,}{np}}(\Delta t)=-\frac{1}{2}\log [1- < {x}_{n}(t),{x}_{p}(t+\Delta t) >^{2} ]$$

9 $${\mathrm{FS}}_{{\mathrm{reversed}},{np}}(\Delta t)=-\frac{1}{2}\log [1- < {x}_{n}^{(r)}(t),{y}_{p}^{(r)}(t+\Delta t) >^{2} ]$$

These matrices, representing the functional temporal dependencies, are based on the mutual information derived from the respective time-shifted correlations^43,109^.

${\mathrm{FS}}_{\mathrm{diff},{np}}$ is a matrix containing the squared differences of the elements between the forward and reversed matrices:

10 $${\mathrm{FS}}_{{\mathrm{diff}},{np}}={(\mathrm{FS}}_{{\mathrm{forward}},{np}}(T)-{\mathrm{FS}}_{{\mathrm{reversed}},{np}}{(T))}^{2}$$

where each element reflects the irreversibility level for that region pair. We describe the level of hierarchy as the standard deviation of the elements of the matrix ${\mathrm{FS}}_{{\mathrm{diff}},{np}}$^43,109^.

Irreversibility between two regions occurs when the interaction between them is asymmetric, such that one region sends more information to the other than it receives from it^43,109^. For each region, its mean irreversibility with the rest of the brain, therefore, quantifies how far it is from equilibrium between sending and receiving. The variability across regions of this send–receive imbalance provides a measure of hierarchical organization, analogous to the ignition-based hierarchy. If this variability is high, it means that regions vary widely in their preference for sending or receiving signals, reflecting greater hierarchical character (in terms of directedness rather than local–global ignition) of the functional organization^43,109^.

#### Synergistic information

The framework of integrated information decomposition unifies integrated information theory (IIT) and partial information decomposition (PID) to decompose information flow into interpretable, disjoint parts. For a brief introduction to PID, see Supplementary Note 3; see also refs. ^111,112^ for detailed explanations. In this section, we provide a brief description of integrated information decomposition and formulas required to compute the results.

In a dynamical system such as the brain, one can calculate the amount of information flowing from the system’s past to its future, known as time-delayed mutual information (TDMI). Specifically, by denoting the past of variables as *X*_*t−τ*_ and *Y*_*t−τ*_ and treating them as sources, and their joint future state (*X*_*t*_, *Y*_*t*_) as target, one can apply the PID framework and decompose the information flowing from past to future as

11 $$\begin{array}{l}{\rm{I}}({X}_{t-\tau },{Y}_{t-\tau };{X}_{t},{Y}_{t})={\rm{Red}}({X}_{t-\tau },{Y}_{t-\tau };{X}_{t},{Y}_{{\rm{t}}})+{\rm{Un}}({X}_{t-\tau };{{X}_{t},{Y}_{t}|Y}_{t-\tau })\\\qquad\qquad\qquad\qquad\quad+{\rm{Un}}({Y}_{t-\tau };{{X}_{t},{Y}_{t}|X}_{t-\tau })+{\rm{Syn}}({X}_{t-\tau },{Y}_{t-\tau };{X}_{t},{Y}_{t})\end{array}$$

Above, Un corresponds to the unique information that one source provides but the other does not; Red is the redundancy between both sources; and Syn is their synergy: information that neither *X* nor *Y* alone can provide but that can be obtained by considering *X* and *Y* together. Applying integrated information decomposition to this quantity allows us to distinguish among redundant, unique and synergistic information shared with respect to the future variables *X*_*t*_, *Y*_*t*_ (refs. ^111,112^). Notably, this framework has identified a stronger notion of redundancy, in which information is shared by *X* and *Y* in both past and future^112^. Accordingly, using the MMI-ΦID decomposition for Gaussian variables, we use

12 $$\mathrm{Red}(X,Y)=\min \{{I}({X}_{t-\tau };{X}_{t}),{I}({X}_{t-\tau };{Y}_{{t}}),{I}({Y}_{t-\tau };{X}_{t}),{I}({Y}_{t-\tau };{Y}_{t})\}$$

Equivalently, this measure corresponds to the information that was redundantly carried by *X* and *Y* in the past and is also redundantly carried redundantly by the two sources in the future. Using this definition of redundancy, we can then solve the system of equations and recover how each type of information (synergistic, unique and redundant) evolves over time. Of these, we focus on the temporally persistent synergy (denoted by ${I}_{\partial }^{\{12\}\to \{12\}}$ in standard PID notation): information that was synergistic in the past and remains synergistic in the future.

Although there is ongoing research on the advantages of different information decompositions for discrete data, several decompositions converge into the same simple form for the case of univariate Gaussian variables^135^. Known as minimum mutual information PID (MMI-PID), this decomposition quantifies redundancy in terms of the minimum mutual information of each individual source with the target; synergy, then, becomes identified with the additional information provided by the weaker source once the stronger source is known. Because linear Gaussian models are sufficiently good descriptors of fMRI time series (and more complex, nonlinear models have been shown to offer no significant advantage^136,137^), here we adopt the MMI-PID decomposition, following our own and others’ previous applications of PID to neuroscientific data^111,112^. MATLAB/Octave and Python code to compute measures of integrated information decomposition of time series with the Gaussian MMI solver is available at https://github.com/Imperial-MIND-lab/integrated-info-decomp.

### Cognitive matching from NeuroSynth

We recently introduced ‘cognitive matching’ in ref. ^103^. For consistency, we report this procedure using the same wording as in ref. ^103^: ‘Continuous measures of the association between voxels and cognitive categories were obtained from NeuroSynth, an automated term-based meta-analytic tool that synthesizes results from more than 14,000 published fMRI studies by searching for high-frequency key words (such as ‘pain’ and ‘attention’ terms) that are systematically mentioned in the papers alongside fMRI voxel coordinates (https://github.com/neurosynth/neurosynth), using the volumetric association test maps^117^. This measure of association strength is the tendency that a given term is reported in the functional neuroimaging study if there is activation observed at a given voxel. Note that NeuroSynth does not distinguish between areas that are activated or deactivated in relation to the term of interest, nor the degree of activation, only that certain brain areas are frequently reported in conjunction with certain words.

Although more than 1,000 terms are catalogued in the NeuroSynth engine, we refine our analysis by focusing on cognitive function, and, therefore, we limit the terms of interest to cognitive and behavioral terms. To avoid introducing a selection bias, we opted for selecting terms in a data-driven fashion instead of selecting terms manually. Therefore, terms were selected from the Cognitive Atlas, a public ontology of cognitive science^138^, which includes a comprehensive list of neurocognitive terms. This approach totaled to *t* = 123 terms, ranging from umbrella terms (‘attention’, ‘emotion’) to specific cognitive processes (‘visual attention’, ‘episodic memory’), behaviors (‘eating’, ‘sleep’) and emotional states (‘fear’, ‘anxiety’) (note that the 123 term-based meta-analytic maps from NeuroSynth do not explicitly exclude patient studies). The Cognitive Atlas subdivision was previously used in conjunction with NeuroSynth^103,139–141^, so we opted for the same approach to make our results comparable to previous reports. The full list of terms included in the present analysis is shown in Supplementary Fig. 24. The probabilistic measure reported by NeuroSynth can be interpreted as a quantitative representation of how regional fluctuations in activity are related to psychological processes. As with the resting-state BOLD data, voxelwise NeuroSynth maps were parcellated into 100 cortical regions according to the Schaefer atlas (or 232 cortical and subcortical regions for the replication with subcortex included).’

For each individual, their parcellated BOLD signals at each point in time were spatially correlated against each of the 123 NeuroSynth maps, producing one value of correlation per NeuroSynth map per BOLD volume. We refer to this operation as ‘cognitive matching’. For each volume, the quality of cognitive matching was quantified as the highest value of (positive) correlation across all 123 NeuroSynth maps. These values were subsequently averaged across all volumes to obtain a single value per condition per participant/simulation.

### Computational memory capacity from reservoir computing

The reservoir computing architecture used in this study consists of a nonlinear recurrent neural network (RNN; reservoir) complemented by a linear readout module that approximates a target signal by means of a linear combination of the signals of output nodes selected from the reservoir^142^. Only the readout module is trained, allowing us to build the reservoir from a generative connectivity matrix that remains unchanged throughout learning.

In this study, the reservoir network’s size (number of nodes) coincides with the number of nodes in each species’ parcellation. For each individual of each species, input nodes were chosen to coincide with visual cortical regions, and the somatomotor regions were used as output nodes, to reflect their respective functional roles^120,143^. A key departure from previous work^18,118^ is that the reservoir network’s topology (how nodes are wired) is not provided directly by the structural connectome but, rather, by the generative connectivity matrices produced by our generative whole-brain model (that is, a re-weighted and signed version of the structural connectivity, which is not only species specific but also individual specific). Concretely, each individual-specific reservoir wiring matrix *W* is obtained by normalizing the generative connectivity matrix by its spectral radius:

13 $$W=\alpha \frac{{W}_{0}}{\rho ({W}_{0})}$$

where ${W}_{0}$ is the original generative connectivity matrix and $\rho ({W}_{0})$ is its spectral radius.

Then, the connection weights are uniformly scaled, so as to produce different *W* matrices with a range of spectral radii $\alpha \in [\mathrm{0.1,1.6}]$ in 0.1 increments. This allows us to parametrically tune the reservoir’s global dynamics^121,144^. The same approach is used for generative connectivity matrices obtained from both cooperative and cooperative–competitive models.

In this study, the reservoir states obey the following discrete-time update equation:

14 $$x\left(t+1\right)=f\left({W}_{\mathrm{in}}u\left(t+1\right)+{Wx}\left(t\right)\right)$$

where $x(t)$ is the vector of nodal reservoir activation states at time $t$; $u\left(t\right)$ is the input signal at time $t$; ${W}_{\mathrm{in}}$ is the input matrix mapping the input signal to the input nodes; $W$ is the reservoir weight matrix; and $f$ is the hyperbolic tangent. An input gain of 0.0001 was used for ${W}_{\mathrm{in}}$ following ref. ^121^.

The readout module is trained following ridge regression as implemented in sklearn using the default $\alpha =0.5$ L2 regularization parameter from the freely available conn2res package (note that this parameter is distinct from the spectral radius parameter above) (https://github.com/netneurolab/conn2res)^120^.

To evaluate computational capacity, we chose the widely used memory capacity task, which measures the reservoir’s ability to encode past stimuli^119,121,143–146^. In this task, the readout module is trained to reproduce a time-delayed version of a random uniformly distributed input signal $u\left(t\right) \sim U(-1,\,1)$. Specifically, $y\left(t\right)=u(t-\tau )$, where $y\left(t\right)$ is the target signal at time $t$ and $\tau$ is the time lag considered. For each time lag, performance at the task was evaluated as the absolute value of the Pearson’s correlation coefficient between the target signal $y\left(t\right)$ and the predicted signal $\hat{y}\left(t\right)$ obtained from the trained readout module. Memory capacity (MC) was then evaluated as the sum of the performance scores across all time lags:

15 $${\mathrm{MC}}=\mathop{\sum }\limits_{\tau }\left|\rho (y,\hat{y})\right|$$

In other words, we let the input signal propagate between nodes of the artificial neural network (representing brain regions) through the reservoir (network of connections between regions), and a linear unit was trained to reproduce a delayed representation of the input signal, based on activation of the output nodes^120,122^.

We generated 4,050 input signal timepoints and used a 70:30 train:test split ratio. Reservoir states were then simulated separately for the training and testing input time series. The first 50 timepoints of both resulting reservoir statesʼ time series were discarded to account for initial transients. Memory capacity was evaluated only in the test time series across 20 time lags, monotonically increased in one-timepoint steps in the range [1,20]. To increase the robustness of our estimates, we repeated this procedure and evaluated memory capacity across 10 simulations, selecting half of the visual regions ($K$) as input nodes at random each time and subsequently averaging performance across simulations. Finally, this assessment was repeated across each spectral radius, and we retained the best performance across all spectral radii considered for each generative connectivity matrix, such that each input matrix was endowed with dynamics that maximize its performance. Altogether, within each species, we obtain individual-specific reservoirs based on the input wiring matrix ${W}_{0}$ corresponding to the generative connectivity matrix of each individual produced by the optimized Hopf model.

### Network properties

#### Modularity

The network modularity function quantifies the extent to which a network can be partitioned such that the number of within-group edges is maximized and the density of between-group edges is minimized. We employed an implementation of Newman’s spectral modularity algorithm available in the Brain Connectivity Toolbox (BCT^147,148^).

#### Clustering coefficient

The clustering coefficient of node *i* (*C*_*i*_) is a node-specific measure of how well connected a node’s neighborhood is; it is calculated as the fraction of neighbors of the node that are also neighbors of each other:

16 $${C}_{i}=\frac{{2t}_{i}}{{k}_{i}({k}_{i}-\,1)}$$

where *t*_*i*_ is the number of triangles around node *i*, and ${k}_{i}$ is the number of edges connected to node *i*. An overall measure of clustering for the entire network is obtained by averaging the nodes’ clustering coefficients. We used the implementation of clustering coefficient available in the BCT^147,148^.

### Surrogate models

To assess the importance of biological organization for our model, we tested the main cooperative−competitive model (that is, the one initialized from empirical structural connectivity) against several surrogate models, each removing different features of biological organisation.

- Binarized null model: This model preserves the network topology of the input structural connectome but erases biological information about weight heterogeneity, setting all to the same uniform value.
- Topology-rewired null model: In this model, existing connections in the input structural connectome are replaced with randomly placed non-existing connections of equal weight. Therefore, this model preserves weight heterogeneity but assesses the relevance of network topology^149^.
- We also implement an alternative version of the topology-rewired null model, where the randomization is constrained to preserve the degree of each node, such that each node makes the same number of connections as in the original structural connectome^150^. This second model, therefore, evaluates the role of biological network topology beyond what is conferred by degree alone.

Each of these models is optimized following the same procedure as the main model, thereby disentangling the improved performance due to presence of competitive interactions from the role of weight heterogeneity and network topology of the connectome.

### Circular-shifted surrogates

To assess whether model performance is sensitive to disrupting the biological relationships between input time series, we use a circular-shifted surrogate. For each BOLD dataset, a surrogate is generated by circularly shifting regional time series by a random (integer) number of timepoints^50,84^. This procedure exactly preserves time series properties that are invariant to the order of timepoints, such as the mean and standard deviation. Properties such as autocorrelation and power spectrum are also approximately preserved on average. However, the synchrony (co-fluctuation) between different regions observed in the biological data is disrupted^50,84^.

### Statistical reporting

Statistical significance of comparisons between the cooperative-only model and the cooperative–competitive model was assessed using a resampling-based, paired-sample *t*-test. This non-parametric implementation of the test ensures robustness to violations of the normality assumption, which was not formally tested. Effect sizes are provided as Hedge’s measure of standardized difference, *g*, which is analogous to Cohen’s *d* but recommended for smaller sample sizes, such as the ones available in the present study.

### Reporting summary

Further information on research design is available in the Nature Portfolio Reporting Summary linked to this article.

## Online content

Any methods, additional references, Nature Portfolio reporting summaries, source data, extended data, supplementary information, acknowledgements, peer review information; details of author contributions and competing interests; and statements of data and code availability are available at 10.1038/s41593-026-02205-3.

## Supplementary information

Supplementary Information Supplementary Notes 1–3, Methods, Figs. 1–24 and References.

Reporting Summary

Supplementary Table 1 Statistical results for human dataset.

Supplementary Table 2 Statistical results for macaque dataset.

Supplementary Table 3 Statistical results for mouse dataset.

## Source data

Source Data Fig. 2 Statistical source data for Fig. 2.

Source Data Fig. 3 Statistical source data for Fig. 3.

Source Data Fig. 4 Statistical source data for Fig. 4.

Source Data Fig. 5 Statistical source data for Fig. 5.

Source Data Fig. 6 Statistical source data for Fig. 6.

Source Data Fig. 7 Statistical source data for Fig. 7.

Source Data Fig. 8 Statistical source data for Fig. 8.

Source Data Extended Data Fig. 1 Statistical source data for Extended Data Fig. 1.

Source Data Extended Data Fig. 2 Statistical source data for Extended Data Fig. 2.

Source Data Extended Data Fig. 3 Statistical source data for Extended Data Fig. 3.

Source Data Extended Data Fig. 4 Statistical source data for Extended Data Fig. 4.

Source Data Extended Data Fig. 5 Statistical source data for Extended Data Fig. 5.

Source Data Extended Data Fig. 6 Statistical source data for Extended Data Fig. 6.

Source Data Extended Data Fig. 7 Statistical source data for Extended Data Fig. 7.

Source Data Extended Data Fig. 8 Statistical source data for Extended Data Fig. 8.

## Data Availability

The Human Connectome Project functional and structural datasets are freely available from http://www.humanconnectome.org/. Macaque fMRI data are available from the PRIMatE Data Exchange (PRIME-DE) through the Neuroimaging Informatics Tools and Resources Clearinghouse (NITRC; http://fcon_1000.projects.nitrc.org/indi/indiPRIME.html). The macaque connectome is available on Zenodo at 10.5281/zenodo.1471588. The CoCoMac database on which it is based is also available online at http://cocomac.g-node.org/main/index.php?. Mouse functional and structural connectome data are available from author A.G. NeuroSynth is available at https://neurosynth.org/. The original macaque cortical gene expression and cell type density data from ref. ^151^ are available at https://macaque.digital-brain.cn/spatial-omics. The dataset is provided by the Brain Science Data Center, Chinese Academy of Sciences (https://braindatacenter.cn/). The original macaque receptor density data from autoradiography are available from https://balsa.wustl.edu/study/P2Nql and https://search.kg.ebrains.eu/instances/de62abc1-7252-4774-9965-5040f5e8fb6b97. The original map of macaque intracortical myelination from T1w/T2w ratio from ref. ^97^ is available at https://balsa.wustl.edu/study/P2Nql. Mouse cell type data are available as described in ref. ^152^. Human gene expression data^153^ are available from the Allen Human Brain Atlas at http://human.brain-map.org/static/download. Mouse gene expression data^154^ are available at https://mouse.brain-map.org/. Human cell type data are available as described in ref. ^155^. Human receptor density data are available online from the neuromaps toolbox. Source data are provided with this paper.

## Code availability

MATLAB and C++ code for the Hopf model with cooperative and competitive interactions is provided at https://github.com/Hana-Ali/competitive-cooperative-hopf.git.

MATLAB/Octave and Python code (version 1.0) to compute measures of integrated information decomposition of time series with the Gaussian MMI solver is available at https://github.com/Imperial-MIND-lab/integrated-info-decomp. The conn2res Python toolbox for reservoir computing (version 1.0.0) is available at https://github.com/netneurolab/conn2res. The Brain Connectivity Toolbox code (version 2019-03-03) used for graph-theoretical analyses and network randomization is freely available online (https://sites.google.com/site/bctnet/). The Python processing for PreClinical data pipeline, Pypreclin version 1.0.1, is freely available at https://github.com/neurospin/pypreclin. The FMRIB Software Library (FSL) is freely available online (http://www.fmrib.ox.ac.uk/fsl/; version accessed on 4 February 2018). CONN toolbox version 17f is freely available at http://www.nitrc.org/projects/conn/. DSI Studio is freely available at https://dsi-studio.labsolver.org/. Java Information Dynamics Toolbox version 1.5 is freely available online (https://github.com/jlizier/jidt). The latest version of the BigBrainWarp toolbox is freely available online (https://bigbrainwarp.readthedocs.io). The abagen toolbox (version 0.1.4) for processing of the AHBA human transcriptomic dataset is available at https://abagen.readthedocs.io/. The neuromaps toolbox (version 0.0.5) is available at https://netneurolab.github.io/neuromaps/.
