# Supplementary material for: Competitive interactions shape mammalian brain network dynamics and computation
Source: Nat Neurosci. 2026 Mar 11;29(4):915–33. doi: 10.1038/s41593-026-02205-3 (PMC13061630; doi:10.1038/s41593-026-02205-3)
Supplement: Supplementary file 1 — Supplementary Notes 1–3, Methods, Figs. 1–24 and References. [file 41593_2026_2205_MOESM1_ESM.pdf]

nature neuroscience

Supplementary information https://doi.org/10.1038/s41593-026-02205-3

Competitive interactions shape mammalian brain network dynamics and computation

In the format provided by the authors and unedited

Supplementary Note 1

Sanz Perl et al[1] implemented an exact mean-field whole-brain model based on inhibitory and excitatory integrate-and-fire neurons; when they explicitly endowed the model with oscillatory dynamics, they found that this much more biologically detailed model was also poised just below the critical Hopf bifurcation. Moreover, this exact mean-field model and the Hopf model were equally good at fitting static and dynamic empirical data from resting-state fMRI recordings of healthy participants[1]. Likewise, Piccinini and colleagues [2] demonstrated that among a wide range of possible connectome-based models, human fMRI data is most accurately represented by a Hopf model poised just below the bifurcation point. Similar conclusions were also reached by Sip et al.[3] who set out to independently validate the methodologies used in traditional connectome-based modelling approaches. They developed a framework to learn the functional form of the neural mass model in a data-driven fashion from the observed dynamics in resting-state fMRI. Crucially, they found that the regional and network-level dynamics can be described as noisy fluctuations around a single fixed point, which is accurately captured by a Hopf bifurcation operating just below the bifurcation[3] – just like the one used here.

Supplementary Note 2

The fact that we work with generative connectivity enables us to draw inferences about the role of competitive interactions for generating the kinds of dynamics observed in empirical fMRI data. However, it is important to emphasise that our present modelling framework does not incorporate the level of biological detail that would be required to derive specific hypotheses about the underlying neurobiology though which competitive interactions are implemented in the brain. The mapping from excitatory or inhibitory synaptic polarity of a long-range neuronal projection to its overall macroscale functional effect is not one-to-one. Rather, it depends on circuit context, synaptic integration, and neuromodulatory regime (among other factors). A long-range excitatory projection could yield an overall suppressive/competitive effect if it were exciting a local inhibitory circuit at its target location, as suggested by the recent model of [4]. In the same vein, an overall cooperative functional effect could arise from a long-range inhibitory projection that provides dis-inhibition at the target site. Thus, we reiterate that macroscale competitive interaction between regions does not equate with synaptic inhibition. The converse also holds: inhibition need not imply competition, since depending on circuit context, circuits of inhibitory connections may also have cooperative effects[5,6]. Altogether, more biologically detailed models that explicitly incorporate such alternative circuits will be necessary, to distinguish between these options - in combination with experimental work and approaches such as MR spectroscopy [7,8].

By definition, every model is an abstraction and a simplification of the true biology, and ours is no exception. Models exist on a continuum of biological realism and ensuing complexity, and a balance between the two needs to be struck[9–11]. Indeed, it is important to bear in mind that even today's most detailed models that incorporate different neuronal populations fall drastically short of accounting for the full diversity of over 260 cell types of the primate cortex [12]. Here we chose the Hopf model on the edge of bifurcation because of its capacity to reproduce many dynamical behaviours of macroscopic fMRI signals, including both asynchronous and synchronous (oscillatory) dynamics, with performance comparable to more complex exact mean-field models[1–3], while also remaining computationally feasible to optimise using our framework. The Hopf model is less detailed and arguably less realistic than a dynamic mean-field or neural mass model, or Jansen-Rit, or a Wilson-Cowan models; and arguably more realistic than the Kuramoto, Ising, cellular-automata, or random-walker models - all of which have found fruitful use in the published neuroscience literature (e.g., [9–11,13–26]). We expect that future work will be able to build on our modelling framework by incorporating competitive interactions into more detailed models that explicitly implement distinct excitatory and inhibitory circuits, to provide additional insight about the potential neurobiological circuitry.

In particular, it is important to note that our present approach is distinct from the dynamic causal modelling (DCM) framework for inferring effective connectivity[27–31]. Whereas our model with Hopf oscillators at the edge of bifurcation provides the advantage of reflecting both oscillatory and non-oscillatory dynamics, DCM employs more biologically realistic modelling of regional neuronal dynamics, which can then be interpreted in terms of excitation and inhibition[27–29]. DCM also incorporates an explicit haemodynamic response function to link the neural activity to the measured BOLD signals [28,32] : an advance that we hope to incorporate in future developments of our model to increase its realism. However, the additional biological realism of DCM comes with additional complexity, meaning that until recently DCM was limited to a small number of pre-selected regions. Additionally, in most implementations of

DCM the underlying brain anatomy is not used to constrain the results, such that a long-range inhibitory connection could be inferred between two regions that are not known to have a direct anatomical connections (although structurally-informed versions of DCM are now also available[33,34]). Finally, our model and DCM use different approaches for fitting. Here we used a pseudo-gradient descent whereby network connections are tuned individually (but not independently)[35,36]. While the more principled estimation schemes such as Bayesian model inversion or variational approaches favoured by DCM offer advantages in terms of uncertainty quantification and incorporation of priors beyond SC, these methods are currently not computationally scalable to the kind of whole-brain model considered here. Bringing the two types of models closer to a convergence is a topic of ongoing work.

In turn, our generative modelling approach is also fundamentally distinct from methods for quantifying direct (as opposed to indirect) functional connectivity, such as partial correlation; or directed (as opposed to undirected) functional connectivity, such as transfer entropy and Granger causality, or multiple regression models [27,37,38]. In particular, although these methods are also sometimes referred to as 'effective connectivity' (and can converge under specific assumptions[37]), it is important to note that they do not seek to infer a full generative causal model of the observed BOLD time-series data. Rather, they infer a statistical form of causality from the lag structure of the timeseries. This is distinct from DCM, and also from our present approach, which does take the lag structure into account, but embodies a generative form of causality and involves anatomical constraints and explicit simulation of time-series.

In light of the vastness of the space of possible models, it is worth remarking that our observation of a role of competitive/suppressive interactions in explaining macroscale brain activity (whatever their ultimate biological origin) is corroborated by two recent reports, which used different modelling strategies. Ruffini and colleagues [39] fitted Ising models to binarised fMRI data, recovering a signed approximation to the structural connectome that included negative connections. Tanner and colleagues [38] used a multi-linear regression framework to predict the future of a region's activity from the weighted histories of its structurally-connected neighbours, allowing them to infer the sign, weight, and direction of structural interactions. When subsequently used as the wiring diagram for a generative neural mass model, the signed SC of [38] outperformed the original SC at reproducing the empirical FC. Overall, both approaches converged with our own, in suggesting that our best statistical and generative accounts of structure-function relationships in the brain should incorporate negatively-signed structural connections. Identifying their neurobiological origins remains an exciting challenge for computational and systems neuroscience.

A major strength of the present study is the consistent replication of results across three independent datasets pertaining to three different mammalian species. This approach is in line with a recent call for greater validation of modelling results across different methods for connectome reconstruction (in vivo diffusion tractography and the gold standard of ex vivo tract-tracing)[27]. However, our species-specific datasets also inevitably come with numerous differences. For the functional MRI data, differences include scanner and acquisition parameters. For the structural connectome data, the mouse SC was entirely obtained from axonal tract-tracing, which is considered the current gold-standard [40], whereas the macaque connectome was reconstructed by augmenting diffusion MRI tractography with existing tract-tracing data, and the human structural connectomes were reconstructed entirely from diffusion MRI tractography, as the only technique that can be applied *in vivo*. However, tractography-derived connectomes can exhibit both false positives and false negatives [41–44]. The superiority of tract-tracing may account for the higher quality of fitting observed in mice than in humans

and macaques (indeed, the mouse tract-tracing connectome – and resulting generative connectivity – display clear inter-hemispheric connections, which are notoriously difficult to resolve with diffusion tractography). On the other hand, a single macaque and mouse connectome were used for all animals, whereas for the human data we were able to use subject-specific structural connectomes, demonstrating that our modelling framework is fully applicable to the single-subject case. Overall, the differences between datasets could represent a limitation, if the present study were focused on inter-species differences. However, our interest is rather in the consistencies across these species, which demonstrate the replicability and generalisability of our results. In this context, differences in how the various datasets were acquired may even be seen as an asset: they are evidence that our discoveries do not critically depend on the specific sampling rate or MRI parameters used, nor on the specific technique that is used to reconstruct the structural connectome, but rather reflect more fundamental properties of mammalian brain organisation.

Supplementary Note 3

Partial information decomposition

We begin with Shannon's Mutual information (MI), which quantifies the interdependence between two random variables *X* and *Y*. It is calculated as

$$\mathrm{I(X;Y)} = \mathrm{H(X)} - \mathrm{H(X|Y)} = \mathrm{H(X)} + \mathrm{H(Y)} - \mathrm{H(X,Y)} \tag{S1}$$

where H(*X*) stands for the Shannon entropy of a variable *X*. Above, the first equality states that the mutual information is equal to the reduction in entropy (i.e., uncertainty) about *X* after *Y* is known. Put simply, the mutual information quantifies the information that one variable provides about another [45].

Crucially, Williams and Beer [46] observed that the information that two source variables *X* and *Y* give about a third target variable *Z*, I(*X, Y* ; *Z*), should be decomposable in terms of different *types* of information: information provided by one source but not the other (unique information), by both sources separately (redundant information), or jointly by their combination (synergistic information). Following this intuition, they developed the *Partial Information Decomposition* (PID; [46]) framework, which leads to the following fundamental decomposition:

$$\mathrm{I(X,Y;Z)} = \mathrm{Red(X,Y;Z)} + \mathrm{Un(X;Z\backslash Y)} + \mathrm{Un(Y;Z\backslash X)} + \mathrm{Syn(X,Y;Z)}. \tag{S2}$$

Above, *Un* corresponds to the unique information one source but the other doesn't, *Red* is the redundancy between both sources, and *Syn* is their synergy: information that neither *X* nor *Y* alone can provide, but that can be obtained by considering *X* and *Y* together.

The simplest example of a purely synergistic system is one in which *X* and *Y* are independent fair coins, and *Z* is determined by the exclusive-OR function *Z* = XOR(*X, Y*): i.e., *Z*=0 whenever *X* and *Y* have the same value, and *Z*=1 otherwise. It can be shown that *X* and *Y* are both statistically independent of *Z*, which implies that neither of them provide - by themselves - information about *Z*. However, *X* and *Y* together fully determine *Z*, hence the relationship between *Z* with *X* and *Y* is purely synergistic.

As another example for the case of Gaussian variables (as employed here), consider a 2-node coupled autoregressive process with two parameters: a noise correlation *c* and a coupling parameter *a*. As *c* increases, the system is flooded by "common noise", making the system increasingly redundant because the common noise "swamps" the signal of each node. As *a* increases, each node has a stronger influence both on the other and on the system as a whole, and we expect synergy to increase. Therefore, synergy reflects the joint contribution of parts of the system to the whole that is not driven by common noise. This can be demonstrated empirically [47,48].

Supplementary Methods

Human FMRI data

The dataset of functional and structural neuroimaging data used in this work came from the Human Connectome Project (HCP, http://www.humanconnectome.org/), Release Q3 [49,50]. Per HCP protocol, all subjects gave written informed consent to the HCP consortium. These data contained fMRI and diffusion weighted imaging (DWI) acquisitions from 100 unrelated subjects (54 females and 46 males, mean age = 29.1 + 3.7 years) of the HCP 900 data release[49,50]. All HCP scanning protocols were approved by the local Institutional Review Board at Washington University in St. Louis, and participants were compensated for their time. Detailed information about the acquisition and imaging is provided in the dedicated HCP publications. Briefly: anatomical (T1-weighted) images were acquired in axial orientation, with FOV = 224 × 224 mm, voxel size 0.7 mm3 (isotropic), TR 2,400ms, TE 2.14ms, flip angle 8°. Functional MRI data (1200 volumes) were acquired with EPI sequence, 2 mm isotropic voxel size, TR 720ms, TE 33.1ms, flip angle 52°, 72 slices.

Functional MRI preprocessing and denoising

We used the minimally preprocessed fMRI data from the HCP, which includes bias field correction, functional realignment, motion correction, and spatial normalisation to Montreal Neurological Institute (MNI-152) standard space with 2mm isotropic resampling resolution. We also removed the first 10 volumes, to allow magnetisation to reach steady state. Additional denoising steps were performed using the CONN toolbox (http://www.nitrc.org/projects/conn), version 17f [51].

To reduce noise due to cardiac and motion artifacts, we applied the anatomical CompCor method of denoising the functional data. The anatomical CompCor method (also implemented within the CONN toolbox) involves regressing out of each individual's functional data the first 5 principal components corresponding to white matter signal, and the first 5 components corresponding to cerebrospinal fluid signal, as well as six subject-specific realignment parameters (three translations and three rotations) and their first- order temporal derivatives, and nuisance regressors identified by the artifact detection software *art* [52]. The subject-specific denoised BOLD signal time-series were linearly detrended and band-pass filtered between 0.008 and 0.09 Hz to eliminate both low-frequency drift effects and high-frequency noise.

Human brains were parcellated into 100 regions of interest (ROIs) covering the entire cortex. The 100 cortical ROIs were obtained from the scale-100 version of the recent local-global functional parcellation of Schaefer et al (2018) [53]. We replicated our main results using the scale-200 Schaefer cortical atlas, augmented with augmented with 32 subcortical ROIs from a recent subcortical functional parcellation [54]. We refer to this 232-ROI parcellation as the augmented "Schaefer-232" parcellation. The timecourses of denoised BOLD signals were averaged between all voxels belonging to a given atlas-derived ROI, using the CONN toolbox.

Macaque FMRI data

The non-human primate MRI data were made available as part of the Primate neuroimaging Data-Exchange (PRIME-DE) monkey MRI data sharing initiative, a recently introduced open resource for non-human primate imaging [55].

Macaque dataset description

We used fMRI data from rhesus macaques (Macaca mulatta) scanned at Newcastle University. This samples includes 14 exemplars (12 male, 2 female); Age distribution: 3.9-13.14 years; Weight distribution: 7.2-18 kg (full sample description available online: http://fcon_1000.projects.nitrc.org/indi/PRIME/files/newcastle.csv and http://fcon_1000.projects.nitrc.org/indi/PRIME/newcastle.html).

Out of the 14 total animals present in the Newcastle sample, 10 had awake resting-state fMRI data; of these 10, all except the first had two scanning sessions available: to maximise our statistical power, these repeated sessions were included in the analysis. Thus, the total was 19 distinct sessions across 10 individual macaques, as in our previous publication [56].

Ethics approval: All of the animal procedures performed were approved by the UK Home Office and comply with the Animal Scientific Procedures Act (1986) on the care and use of animals in research and with the European Directive on the protection of animals used in research (2010/63/EU). We support the Animal Research Reporting of In Vivo Experiments (ARRIVE) principles on reporting animal research. All persons involved in this project were Home Office certified and the work was strictly regulated by the U.K. Home Office. Local Animal Welfare Review Body (AWERB) approval was obtained. The 3Rs principles compliance and assessment was conducted by National Centre for 3Rs (NC3Rs). Animal in Sciences Committee (UK) approval was obtained as part of the Home Office Project License approval.

Animal care and housing: All animals were housed and cared for in a group-housed colony, and animals performed behavioural training on various tasks for auditory and visual neuroscience. No training took place prior to MRI scanning.

Macaque MRI acquisition

Animals were scanned in a vertical Bruker 4.7T primate dedicated scanner, with single channel or 4-8 channel parallel imaging coils used. No contrast agent was used. Optimization of the magnetic field prior to data acquisition was performed by means of 2nd order shim, Bruker and custom scanning sequence optimisation.

Animals were scanned upright, with MRI compatible head-post or non-invasive head immobilisation, and working on tasks or at rest (here, only resting-state scans were included). Eye tracking, video and audio monitoring were employed during scanning.

Resting-state scanning was performed for 21.6 minutes, with a TR of 2600ms, 17ms TE, Effective Echo Spacing of 0.63ms, voxels size 1.22 x 1.22 x 1.24. Phase Encoding Direction: Encoded in columns. Structural scans comprised a T1 structural, MDEFT sequence with the following parameters: TE: 6ms; TR: 750 ms; Inversion delay: 700ms; Number of slices: 22;

In-plane field of view: 12.8 x 9.6cm2 on a grid of 256 x 192 voxels; Voxel resolution: 0.5 x 0.5 x 2mm; Number of segments: 8.

Macaque functional MRI preprocessing and denoising

The macaque MRI data were preprocessed using the recently developed pipeline for non-human primate MRI analysis, *Pypreclin* (https://github.com/neurospin/pypreclin), which addresses several specificities of monkey research. The pipeline is described in detail in the associated publication [57]. Briefly, it includes the following steps: (i) Slice-timing correction. (ii) Correction for the motion-induced, time-dependent B0 inhomogeneities. (iii) Reorientation from acquisition position to template; here, we used the recently developed National Institute of Mental Health Macaque Template (NMT): a high-resolution template of the average macaque brain generated from in vivo MRI of 31 rhesus macaques (Macaca mulatta) [58]. (iv) Realignment to the middle volume using FSL MCFLIRT function. (v) Normalisation and masking using Joe's Image Program (JIP) -align routine (http://www.nmr.mgh .harvard.edu/~jbm/jip/, Joe Mandeville, Massachusetts General Hospital, Harvard University, MA, USA), which is specifically designed for preclinical studies: the normalization step aligns (affine) and warps (non-linear alignment using distortion field) the anatomical data into a generic template space. (vi) B1 field correction for low-frequency intensity non-uniformities present in the data. (vii) Coregistration of functional and anatomical images, using JIP -align to register the mean functional image (moving image) to the anatomical image (fixed image) by applying a rigid transformation. The anatomical brain mask was obtained by warping the template brain mask using the deformation field previously computed during the normalization step. Then, the functional images were aligned with the template space by composing the normalization and coregistration spatial transformations.

Denoising: The aCompCor denoising method implemented in the CONN toolbox was used to denoise the macaque functional MRI data, to ensure consistency with the human data analysis pipeline. White matter and CSF masks were obtained from the corresponding probabilistic tissue maps of the high-resolution NMT template (eroded by 1 voxel); their first five principal components were regressed out of the functional data, as well as linear trends and 6 motion parameters (3 translations and 3 rotations) and their first derivatives. Finally, data were bandpass-filtered in the range of 0.008-0.09Hz Hz, as in our previous work with these data [56]. Macaque functional data were parcellated according to the 82-ROI "Regional Mapping" cortical atlas of Kotter and Wanke [59], nonlinearly registered to the NMT template used for preprocessing.

Mouse FMRI data

The mouse fMRI data used here have been previously reported [60]. For clarity and consistency of reporting, where possible we use the same wording as in the original publication [60].

Animals and ethics

In vivo experiments were conducted in accordance with the Italian law (DL 26/214, EU 63/2010, Ministero della Sanita, Roma) and with the National Institute of Health recommendations for the care and use of laboratory animals [60]. The animal research protocols

for this study were reviewed and approved by the Italian Ministry of Health and the animal care committee of Istituto Italiano di Tecnologia (IIT). All surgeries were performed under anesthesia.

Adult (< 6 months old) male C57BL/6J mice were used throughout the study. Mice were group housed in a 12:12 hours light-dark cycle in individually ventilated cages with access to food and water ad libitum and with temperature maintained at 21 ± 1 degrees centigrade and humidity at 60 ± 10%. All the imaged mice were bred in the same vivarium and scanned with the same MRI scanner and imaging protocol employed for the awake scans (see below).

Experimental groups and datasets

A group of mice (n = 10, awake dataset) underwent head-post surgery, scanner habituation and fMRI image acquisitions as described below. See [60] for the full surgical, habituation, and scanner protocol. The scans so obtained constitute the awake rsfMRI dataset we used throughout our study.

MRI data acquisition

For awake scanning, the mouse was secured using an implanted headpost the custom-made MRI-compatible animal cradle and the body of the mouse was gently restrained (for details of the headpost implantation and habituation protocol, see the original publication [60]).

All scans were acquired at the IIT laboratory in Rovereto (Italy) on a 7.0 Tesla MRI scanner (Bruker Biospin, Ettlingen) with a BGA-9 gradient set, a 72 mm birdcage transmit coil, and a four-channel solenoid receive coil. Awake rsfMRI scans were acquired using a single-shot echo planar imaging (EPI) sequence with the following parameters: TR/TE=1000/15 ms, flip angle=60 degrees, matrix=100 x 100, FOV=2.3 x 2.3 cm, 18 coronal slices (voxel-size 230 x 230 x 600 mm), slice thickness=600 mm and 1920 time points, for a total time of 32 minutes.

Functional MRI preprocessing, denoising, and timeseries extraction

Preprocessing of fMRI images was carried out as described in previous work [60]. Briefly, the first 2 minutes of the time series were removed to account for thermal gradient equilibration. RsfMRI timeseries were then time despiked (3dDespike, AFNI), motion corrected (MCFLIRT, FSL), skull stripped (FAST, FSL) and spatially registered (ANTs registration suite) to an in-house mouse brain template with a spatial resolution of 0.23 x 0.23 x 0.6mm^3. Denoising involved the regression of 25 nuisance parameters. These were: average cerebral spinal fluid signal plus 24 motion parameters determined from the 3 translation and rotation parameters estimated during motion correction, their temporal derivatives and corresponding squared regressors. No global signal regression was employed. In-scanner head motion was quantified via calculations of frame-wise displacement (FD). Average FD levels in awake conditions were comparable to those obtained in anesthetized animals (halothane) under artificial ventilation ($p = 0.13$, t-test) [60]. To rule out a contribution of residual head-motion, we further introduced frame-wise fMRI scrubbing (FD > 0.075 mm). The resulting time series were band-pass filtered (0.01-0.1 Hz band) and then spatially smoothed with a Gaussian kernel of 0.5 mm full width at half maximum. The timeseries were trimmed to ensure that the same number of timepoints were included for all animals, resulting in 1414 volumes per animal. Finally data were parcellated into 72 cortical symmetric regions from the Allen Mouse Brain Atlas (CCFv3).

Species-specific connectomes

Human structural connectome

We used diffusion-weighted imaging (DWI) MRI data from the Human Connectome Project. The DWI acquisition protocol is covered in detail elsewhere [50]. The diffusion MRI scan was conducted on a Siemens 3T Skyra scanner using a 2D spin-echo single-shot multiband EPI sequence with a multi-band factor of 3 and monopolar gradient pulse. The spatial resolution was 1.25 mm isotropic. TR = 5500 ms, TE = 89.50 ms. The b-values were 1000, 2000, and 3000 s/mm2. The total number of diffusion sampling directions was 90, 90, and 90 for each of the shells in addition to 6 b0 images. We used the version of the data made available in DSI Studio-compatible format at http://brain.labsolver.org/diffusion-mri-templates/hcp-842-hcp-1021 [61].

We adopted previously reported procedures to reconstruct the human connectome from DWI data. The minimally-preprocessed DWI HCP data were corrected for eddy current and susceptibility artifact. After preprocessing, the DTI data were reconstructed using the model-free q-space diffeomorphic reconstruction algorithm (QSDR) implemented in DSI Studio (www.dsi-studio.labsolver.org) [62], following our previous work [63]. QSDR initially reconstructs DWI data in native space, and subsequently computes values of quantitative anisotropy (QA) in each voxel, based on which DSI Studio performs a nonlinear warp from native space to a template QA volume in Montreal Neurological Institute (MNI) space. Once in MNI standard space, spin density functions are reconstructed, with a mean diffusion distance of 1.25 mm with three fiber orientations per voxel [62]. Finally, fiber tracking was carried out by means of DSI Studio's own "FACT" deterministic tractography algorithm, requesting 1,000,000 streamlines according to widely adopted parameters [63]: angular cutoff = 55°, step size = 1.0 mm, tract length between 10mm (minimum) and 400mm (maximum), no spin density function smoothing, and QA threshold determined by DWI signal in the cerebro-spinal fluid. Streamlines were automatically rejected if they presented improper termination locations, based on a white matter mask automatically generated by applying a default anisotropy threshold of 0.6 Otsu's threshold to the anisotropy values of the spin density function [63].

For each individual, their structural connectome was reconstructed by drawing an edge between each pair of regions i and j if there were white matter tracts connecting the corresponding brain regions end-to-end; edge weights were quantified as the number of streamlines connecting each pair of regions.

Macaque structural connectome

Anatomical (structural) connectivity data were derived from the recent macaque connectome of [64], which combines diffusion MRI tractrography with axonal tract-tracing studies from the CoCoMac database [65], representing the most complete representation of the macaque connectome currently available. Structural connectivity data are expressed as a matrix in which the 82 cortical regions of interest are displayed in x-axis and y-axis. Each cell of the matrix represents the strength of the anatomical connection between any pair of cortical areas. For consistency with the human structural connectome, a symmetrised connectome was used, as in our previous work [66].

Mouse structural connectome

For the mouse structural connectome, we used a parcellated version of the high-resolution mouse connectome of Coletta *et al.* [67]. Below, we summarise how Coletta and colleagues obtained the high-resolution mouse structural connectome.

The present mouse structural connectome is based on "high-resolution models of the mouse brain connectome (100 μm^3) previously released by Knox and colleagues [68]. The Knox connectome is based on 428 viral microinjection experiments in C57BL/6J male mice obtained from the Allen Mouse Brain Connectivity Atlas (http://connectivity.brain-map.org/). The connectome data were derived from imaging enhanced green fluorescent protein (eGFP)–labeled axonal projections that were then registered to the Allen Mouse Brain Atlas and aggregated according to a voxel-wise interpolation model [68]".

Before constructing the SC matrix, Coletta et al. ensured symmetry along the right-left axis for all the major macrostructures of the mouse brain. To this purpose, they "flipped each macrostructure (isocortex, hippocampal formation, subcortical plate, pallidum, striatum, pons, medulla, midbrain, thalamus, hypothalamus, cerebellum, and olfactory bulb) along the sagittal midline (once for the right hemisphere and once for the left hemisphere) and took the intersection with the respective nonflipped macrostructure. This procedure resulted in the removal of a set of nonsymmetric voxel (total fraction, 8.6%), the vast majority of which reside in fringe white/gray matter or cerebrospinal fluid/gray matter interfaces. The removal of these nonsymmetric voxels did not substantially affect the network structure of the resampled connectome, as assessed with a spatial correlation analysis between the symmetrized and nonsymmetrized right ipsilateral (i.e., squared) connectome". Coletta et al. then filtered out fiber tracts and ventricular spaces, and estimated SC using a resampled version of the recently published voxel scale model of the mouse structural connectome [68], to make the original matrix computationally tractable. Resampling of the Knox et al. connectome was carried out by aggregating neighboring voxels according to a Voronoi diagram based on Euclidean distance between neighboring voxels, preserving the intrinsic architectural foundation of the connectome while minimizing spatial blurring and boundary effects between ontogenically distinct neuroanatomical divisions of the mouse brain, or white/gray matter, and parenchymal/ventricular interfaces (see Coletta et al. for details of the Voronoi aggregation scheme [68]). By averaging the connectivity profile of neighboring voxels based on their relative spatial arrangement, this strategy has also the advantage of mitigating limitations related to the enforced smoothness of source space used by the original kernel interpolation used by [68].

A whole-brain connectome was then built under the assumption of brain symmetry [67]. Forty-four dangling nodes (i.e., nodes with no outgoing connectivity) were next removed from the resulting matrix, resulting in a final weighted and directed 15,314 × 15,314 matrix composed of 0.027-mm3 aggregate Voronoi voxels. The obtained Voronoi diagram made it possible to map the results back into the original 100-µm three-dimensional coordinate system of the Allen Institute mouse brain connectome [CCFv3]. For each pair of the 72 Allen Atlas cortical regions that we employed, their structural connectivity was obtained by averaging the connectivity of the respective constituent voxels.

To facilitate comparison of results between human and other species, the structural connectomes of macaque and mouse were symmetrised, to avoid imposing structural asymmetries and instead allow the model itself to determine the most suitable level of asymmetry (if any). Similarly, since the human and macaque structural connectomes are sparse, whereas the mouse connectome is provided as fully dense, the latter was thresholded to 50%

density. However, we demonstrate that our results are not dependent on either of these methodological choices, as shown in the Supplementary Information where we vary both density and symmetry in each species.

Biological annotations

Covariance of gene expression

Human gene expression from microarray probes

Human gene expression profiles were obtained using microarray data from the Allen Human Brain Atlas (AHBA) [69]. We followed the same preprocessing as recently described [70]. Briefly, the Allen Human Brain Atlas (AHBA) is a publicly available transcriptional atlas containing gene expression data measured with DNA microarrays and sampled from hundreds of histologically validated neuroanatomical structures across six (five male and one female) normal postmortem human brains. We extracted and mapped gene expression data to the 100 cortical ROIs of the Schaefer parcellation using the *abagen* toolbox [71]. Data was pooled between homologous cortical regions to ensure adequate coverage of both left (data from six donors) and right hemisphere (data from two donors). Distances between samples were evaluated on the cortical surface with a 2 mm distance threshold. Only probes where expression measures were above a background threshold in more than 50% of samples were selected. A representative probe for a gene was selected based on highest intensity. 15,633 genes survived these preprocessing and quality assurance steps.

Macaque gene expression from high-resolution spatial transcriptomics

We used cortex-wide macaque gene expression data recently made available by [12], who combined single-nucleus RNA sequencing ("snRNA-seq") with high-resolution, large-field-of view spatial transcriptomics from spatiotemporal enhanced resolution omics sequencing ("stereo-seq") [12]. Specifically, the authors made available (https://macaque.digital-brain.cn/spatial-omics) post-mortem gene expression data covering 143 regions of the left cortical hemisphere of one 6yo male cynomolgus macaque (*Macaca fascicularis*). We refer the reader to [12] for details. Briefly, Chen and colleagues obtained 119 coronal sections at 500-µm spacing, covering the entire cortex of the left hemisphere, which were used for stereo-seq transcriptomics [12]. Adjacent 50-µm thick sections were also acquired for regional microdissection and snRNAseq analysis, as well as 10-µm sections adjacent to each stereo-seq section, which were used for the anatomical parcellation of brain regions via immunostaining [12]. Stereo-seq is a DNA nanoball (DNB) barcoded solid-phase RNA capture method that involves reverse transcription of RNAs released from frozen tissue sections fixated onto the stereo-seq chip, and subsequent PCR amplification. The resulting "amplified barcoded complementary DNA (cDNA) is used as template for library preparation, and sequenced" to obtain high-resolution spatially resolved transcriptomics [12]. Gene expression data were made available by [12] for 143 cortical regions of the left hemisphere, including "prefrontal, frontal, cingulate, somatosensory, insular, auditory, temporal, parietal, occipital and piriform areas". As reported in [12], "for each coronal section, the cortical region and layer parcellation were manually delineated on Stereo-seq data background, based on cytoarchitectual pattern (e.g. cell density, cell size) revealed by total mRNA expression, nucleic acid staining, and NeuN staing of adjacent sections." Since the original parcellation used by [12] is different from the "Regional

Mapping" macaque cortical atlas [59] that was used for our macaque functional MRI data, here we used the version of the macaque gene expression data that was mirrored between hemispheres and mapped onto the "Regional Mapping" macaque cortical atlas, as made available by [72].

Mouse gene expression from in situ hybridization

Mouse gene expression profiles were obtained using in situ hybridization data from the Allen Mouse Brain Atlas [73]. We followed the same preprocessing as recently described [74]. Briefly, the Allen Mouse Brain Atlas consists of data acquired from a pipeline that includes semi-automated riboprobe generation, tissue preparation and sectioning, in-situ hybridization (ISH), imaging, and data post-processing. These data were acquired both sagittally and (for a smaller set of genes) coronally, and were further processed, aligned by the Allen Institute to their Common Coordinate Framework version 3 (CCFv3) reference atlas [75] and summarized voxelwise through a measure termed gene expression energy (defined as the sum of expressing pixel intensity divided by the sum of all pixels in a division), resulting in 3D gene expression images at a 200 μm isotropic resolution. This gene expression energy increases in regions of high expression, and is bounded by zero in regions of no expression. For our study, we used gene expression energy data from the coronal dataset (4345 gene expression images corresponding to 4082 unique genes), because of its whole-brain coverage and data quality. Voxelwise gene expression data were further summarized as normalized mean expression within regions of interest as defined by the CCFv3 reference atlas. For each ROI and each gene, voxelwise expression energy data were averaged over voxels containing valid expression signal.

For each species, we consider a list of evolutionary-conserved brain-related genes pertaining to neurotransmitter receptors and cell type markers [76,77]. These human genes have orthologs in both mouse and macaque, making them comparable across species. From the genes in this list, a total of 81 genes are available and pass our quality control criteria in each of the three species (human, macaque, and mouse). To obtain inter-regional covariance of gene expression, each gene expression pattern was z-scored and then genes were correlated across regions, thereby obtaining a regions-by-regions matrix.

Similarity of position along the PV-SST axis

For each species, we used the transcriptomic data to obtain a proxy for the axis from output-modulating parvalbumin-positive interneurons, to input-modulating somatostatin-positive interneurons (PV-SST axis) identified by Kim, Young and colleagues, [78] as follows. First, we ranked the regional distribution of the *PVALB/Pvalb* gene across cortical regions, and we ranked the regional distribution of the *SST/Sst* gene across cortical regions. Second, we subtracted the *PVALB/Pvalb* rank from the *SST/Sst* rank, obtaining a vector of regional differences in ranks [56], which therefore indicates the relative prevalence in expression of one gene over the other. Finally, we turned this vector into a region-by-region matrix by taking its outer product with itself. In this matrix, negative entries indicate that the corresponding regions are situated on opposite ends of the PV-SST axis, whereas positive entries indicate that the corresponding regions are situated the same end (PV-dominated or SST-dominated) of the PV-SST axis.

Similarity of position along the anatomical hierarchy

For each species, we used maps of intracortical myelination (from T1w:T2w ratio) as an *in vivo* marker of the anatomical cortical hierarchy [76,79,80]. For the human, we used the map available in the *neuromaps* toolbox[81]. For the macaque, we used the map originally shared by [82] and translated to the Regional Mapping macaque cortical parcellation of Kötter and Wanke [59] by [77]. As above, the z-scored vector of regional intracortical myelination was turned into a region-by-region matrix by taking its outer product with itself. In this matrix, negative entries indicate that the corresponding regions are situated on opposite ends of the anatomical hierarchy, whereas positive entries indicate that the corresponding regions are situated the same end of the anatomical hierarchy.

Covariance of cytoarchitectonic composition

Human cell type composition

We obtained cortical maps of 24 cellular classes made available by [83], comprising nine GABAergic inhibitory interneurons (PAX6, SNCG, VIP, LAMP5, LAMP5 LHX6, Chandelier, PVALB, SST CHODL and SST), nine glutamatergic excitatory neurons (L2/3 IT, L4 IT, L5 IT, L6 IT, L5 ET, L5/6 NP, L6 CT, L6b and L6 IT Car3), and six non-neuronal cells (Astro, Endo, VLMC, Oligo, OPC and Micro/PVM). These cell classes were identified in the Allen Institute's bulk sample data [69] based on transcriptional signatures from cortical snRNA-seq data from different cortical areas [84]. From these transcriptional signatures, the regional abundance of each cell type was estimated in available AHBA bulk samples, for each region of the Schaefer atlas [84]. To obtain inter-regional covariance of cytoarchitecture, each cortical cell type pattern was z-scored and then cell types were correlated across regions, thereby obtaining a regions-by-regions matrix of cell type similarity.

Macaque cell type composition

We obtained maps of 23 transcriptomically derived cell types for the macaque cortex, based on data-driven clustering of snRNA-seq gene expression data[12]. They comprise 10 subclasses of excitatory (glutamatergic) neurons, 7 subclasses of GABA-ergic neurons, and 6 subclasses of nonneuronal cells. The 10 glutamatergic neuron subclasses were annotated by their layer preferences (L for layer: L2, L2/3, L2/3/4, L3/4, L3/4/5, L4, L4/5, L4/5/6, L5/6, and L6). The 7 GABAergic neuron subclasses were divided into chandelier cells (CHC) and cells preferentially expressing lysosome-associated membrane protein 5 (labelled LAMP5), vasoactive intestinal peptide (VIP), reelin (RELN), VIP and reelin (VIP-RELN), parvalbumin (PV), somatostatin (SST). The 6 non-neuronal subclasses included astrocytes (ASC), oligodendrocyte precursor cells (OPC), oligodendrocytes (OLG), microglia (MG), endothelial cells (EC), and vascular leptomeningeal cells (VLMC). Since the original parcellation used by [12] is different from the "Regional Mapping" macaque cortical atlas [59] that was used for our macaque functional MRI data, here we used the version of the macaque cell type data that was mapped onto the "Regional Mapping" macaque cortical atlas, as made available by [72]. To obtain inter-regional covariance of cytoarchitecture, each cortical cell type pattern was z-scored and then cell types were correlated across regions, thereby obtaining a regions-by-regions matrix of cell type similarity.

Mouse cell type composition

We obtained maps of mouse cell types from [85] who combined whole brain Nissl and gene expression stains to provide literature-validated estimates of the regional densities of excitatory, inhibitory, and modulatory neurons, astrocytes, oligodendrocytes, and microglia across regions of the Allen Mouse Brain Atlas. To obtain inter-regional covariance of cytoarchitecture, each cortical cell type pattern was z-scored and then cell types were correlated across regions, thereby obtaining a regions-by-regions matrix of cell type similarity.

Covariance of receptor expression

Human receptor density from in vivo Postitron Emission Tomography

Receptor densities were estimated using PET tracer studies for a total of 19 receptors and transporters, across 9 neurotransmitter systems, recently made available by Hansen and colleagues at https://github.com/netneurolab/hansen_receptors[86]. These include dopamine (D1[87], D2[88–91], DAT[92]), norepinephrine (NET[93–96]), serotonin (5-HT1A[97], 5-HT1B[97–100,100–102], 5-HT2A[103], 5-HT4[103], 5-HT6[104,105], 5-HTT[103]), acetylcholine ($\alpha 4\beta 2$[106,107], M1[108], VAChT[109,110]), glutamate (mGluR5[111,112], NMDA[113,114]), GABA (GABA-A[115]), histamine (H3[116]), cannabinoid (CB1[117–120]), and opioid (MOR[121]). Volumetric PET images were registered to the MNI-ICBM 152 nonlinear 2009 (version c, asymmetric) template, averaged across participants within each study, then parcellated and receptors/transporters with more than one mean image of the same tracer (5-HT1b, D2, VAChT) were combined using a weighted average. See the dedicated article by Hansen *et al*[86] for detailed information about each PET dataset and their respective acquisition and limitations. The matrix of similarity of regional gene expression was obtained by correlating the z-scored receptor density patterns.

Macaque receptor density from in vitro autoradiography

In vitro autoradiography data for 14 neurotransmitter receptors were obtained from [82]: *AMPA*, *kainate*, *NMDA*, $GABA_{\mathrm{A}}$, $GABA_{\mathrm{B}}$, $GABA_{\mathrm{A/BZ}}$, M_1, M_2, M_3, α_1, α_2, $5HT_{1\mathrm{A}}$, $5HT_{2\mathrm{A}}$, D_1. The authors applied quantitative in vitro receptor autoradiography to label 14 neurotransmitter receptors in three male *Macaca fascicularis* brains (7.3 $\pm$ 0.6 years old; body weight 6 $\pm$ 0.8 kg) obtained from Covance Preclinical Services, where they were housed and used as control animals for pharmaceutical studies performed in compliance with legal requirements. Animal experimental procedures and husbandry had the approval of the respective Institutional Animal Care and Use Committee and were carried out in accordance with the European Council Directive of 2010 [82]. We refer the reader to the original paper for details [82].

Briefly, the data of density of receptors per neuron were made available for 109 cortical areas of the macaque brain, which were identified based on their cytoarchitecture and receptor-architecture characteristics [82]. Here we used the version of the macaque receptor density data that was mapped onto the "Regional Mapping" macaque cortical atlas, as made available by [72]. The matrix of similarity of regional gene expression was obtained by correlating the z-scored receptor density patterns.

Human laminar similarity

Laminar similarity is estimated from histological data and aims to uncover how similar pairs of cortical regions are in terms of cellular distributions across the cortical layers [122]. Here we followed the same procedure as [122]. Laminar similarity was first introduced in Paquola et al. [123] and has also been referred to as "microstructure profile covariance". Specifically, we use data from the BigBrain, a high-resolution (20 μm) histological reconstruction of a post-mortem brain from a 65 year old male [123,124]. Cell-staining intensity profiles were sampled across 50 equivolumetric surfaces from the pial surface to the white mater surface to estimate laminar variation in neuronal density and soma size. Intensity profiles at various cortical depths can be used to approximately identify boundaries of cortical layers that separate supragranular (cortical layers I–III) granular (cortical layer IV), and infragranular (cortical layers V-VI) layers[122]. The data were obtained on fsaverage surface (164k vertices) from the BigBrainWarp toolbox (https://bigbrainwarp.readthedocs.io) and were parcellated into 100 cortical regions according to the Schaefer atlas, as per [122]. The regions-by-regions laminar similarity matrix was calculated as the partial correlation of cell intensities between pairs of cortical regions, after correcting for the mean intensity across cortical regions.

Supplementary Figures

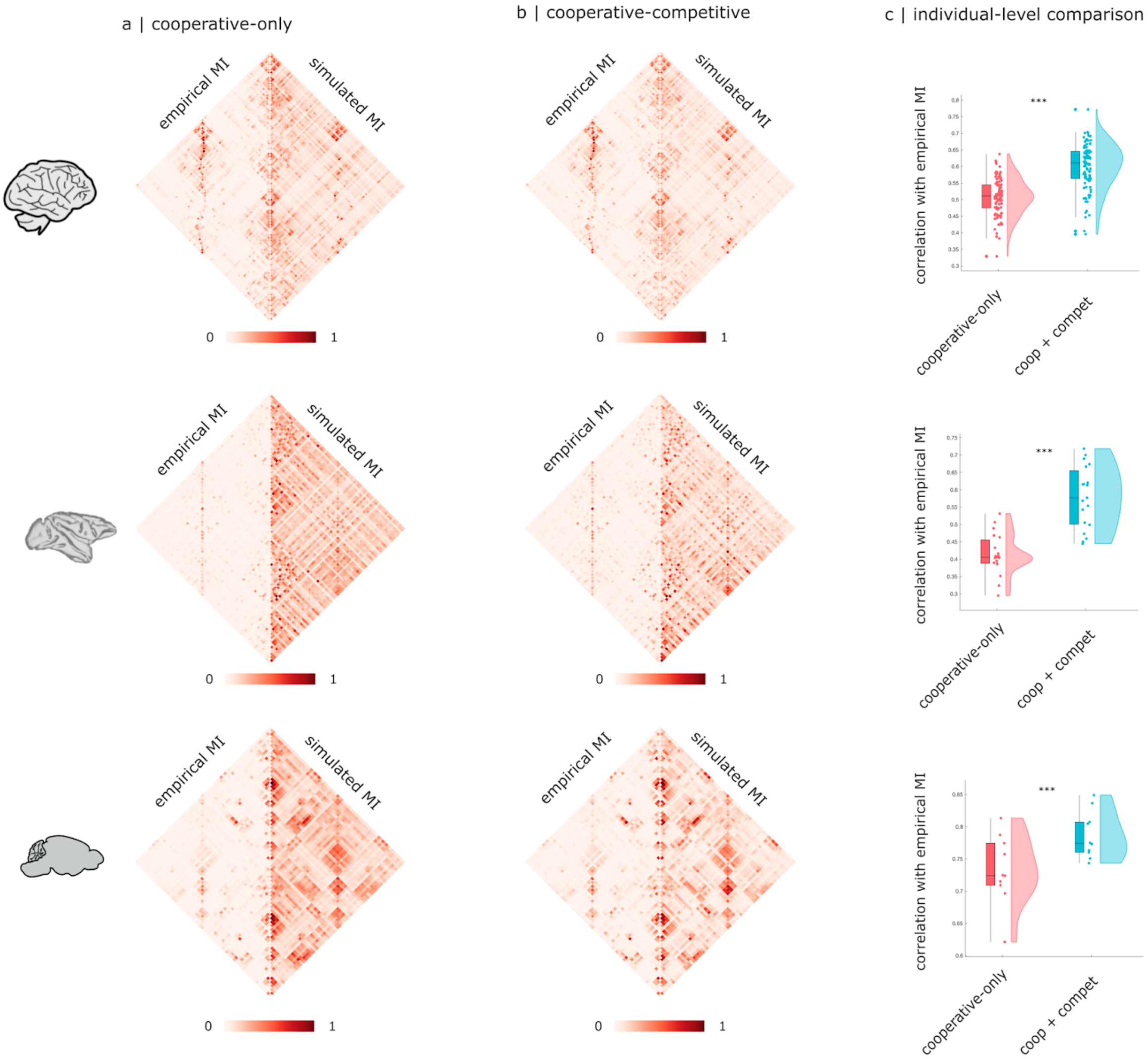

Figure S1. Competitive interactions improve fit to mutual information between regions. Matrices show group-average mutual information (MI) between pairs of regions for empirical data (left half) and simulated data (right half), for the cooperative-only model (first column) and cooperative-competitive model (second column). (a) Cooperative-only model. (b) Cooperative-competitive model. (c) Violin plots show individual-level fit (correlation between empirical and simulated MI matrices). ***, $p < 0.001$ from paired-samples t-tests (two-sided). Box plots: the central lines indicate median values, the bounds of the boxes indicate the 25th and 75th percentiles, the whiskers indicate 1.5× the interquartile range. Each data-point represents one individual scan. Human: n=100 individuals; macaque: n=19 data-points from 10 animals; mouse: n=10 animals.

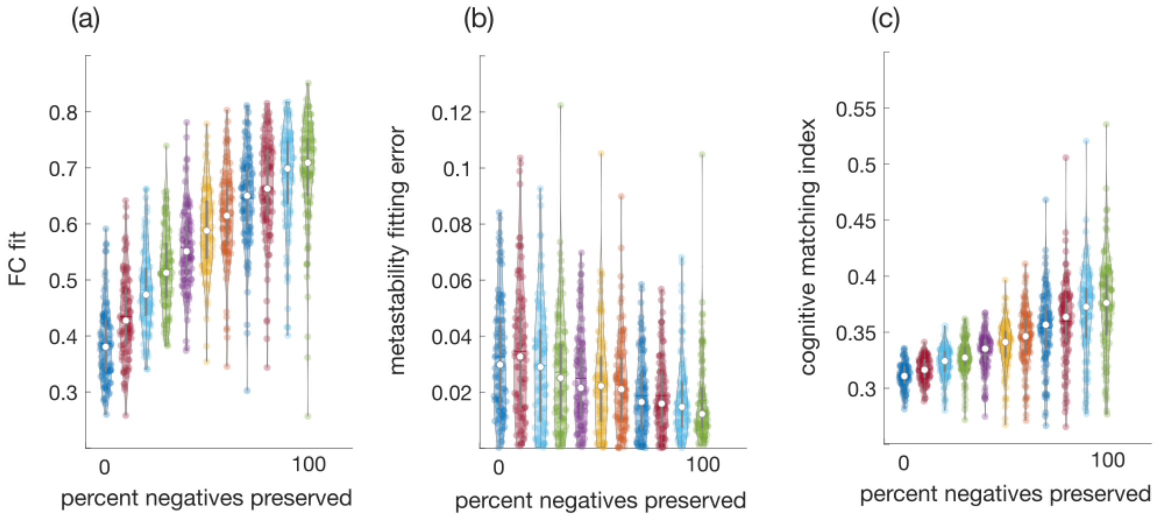

Figure S2. Removing negative generative weights deteriorates model fit | (**a**) Similarity between empirical and simulated FC. (**b**) Difference between empirical and simulated metastability. (**c**) Cognitive matching from NeuroSynth. Violin plots: the central dot indicate median values, the bounds of the boxes indicate the 25th and 75th percentiles, the whiskers indicate 1.5× the interquartile range. Each data-point represents one individual scan (n=100 human individuals).

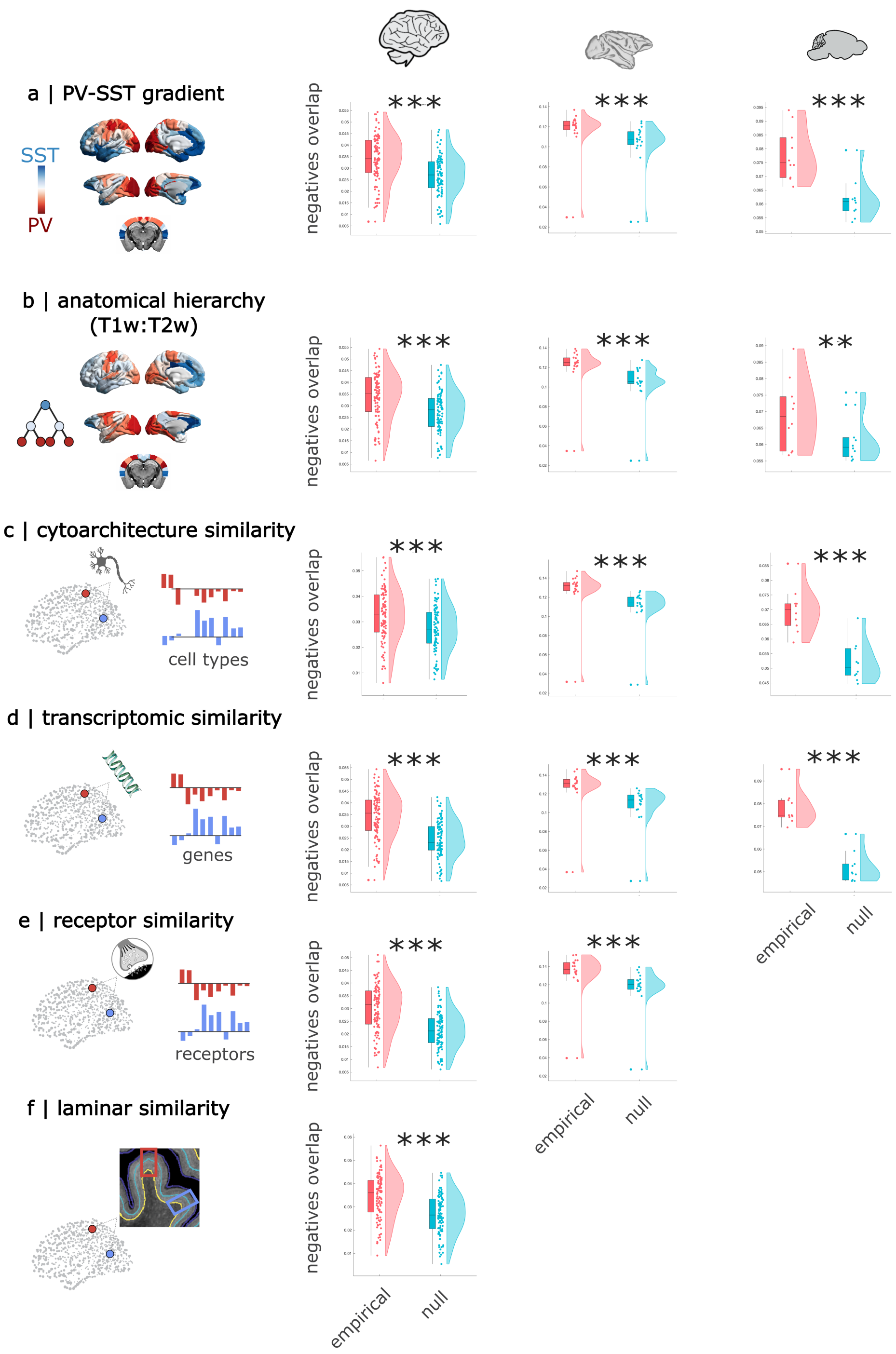

Figure S3 Competitive interactions link regions with opposite biological annotations significantly more than chance | (**a**) Axis from output-modulating parvalbumin-positive interneurons, to input-modulating somatostatin-positive interneurons, quantified as the difference in rank between *PVALB/Pvalb* and *SST/Sst* gene expression in each species ('PV-SST axis)'. (**b**) Anatomical hierarchy quantified by intracortical myelination from T1w:T2w MRI. Matrix displays the similarity between regions' locations along the anatomical hierarchy, obtained as the outer product of the z-scored T1w:T2w vectors, such that regions at opposite ends of the hierarchy will have a negative link between them. Mouse: $p = 0.012$. (**c**) Cytoarchitectonic covariance. (**d**) Covariance of regional gene expression. (**e**) Covariance of regional receptor density (only available for human and macaque). (**f**) Covariance of regional laminar profiles from the BigBrain human dataset. Violin plots indicate the prevalence of overlaps between negative connections in the inferred connectivity matrix and in the biological networks; or if connection signs were random. (a-f) ***, $p < 0.001$ from paired-samples t-tests (two-sided). Box plots: the central lines indicate median values, the bounds of the boxes indicate the 25th and 75th percentiles, the whiskers indicate 1.5× the interquartile range. Each data-point represents one individual scan. Human: n=100 individuals; macaque: n=19 data-points from 10 animals; mouse: n=10 animals.

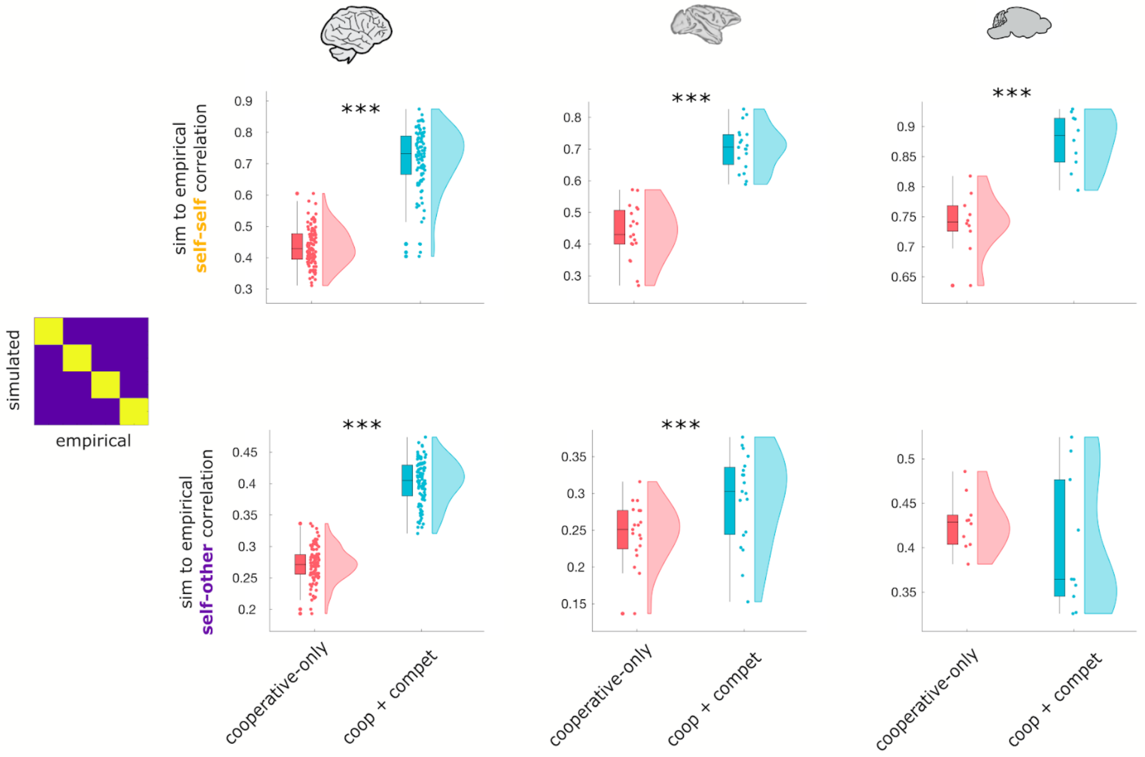

Figure S4. Model with competitive interactions provides superior match to the original individual's FC, and superior generalisation to other individuals' FC. (Top) Correlation between the empirical and simulated FC of each subject is significantly higher for the model with competitive interactions. (Bottom) FC generated from the model with competitive interactions is significantly more correlated with the FC of other individuals, indicating superior generalisation. Note that this increase in generalisability does not come at the expense of subject-specificity, because the self-self increase (diagonal entries in the identifiability matrix) is significantly larger than the self-other increase in correlation (off-diagonal entries in the identifiability matrix), resulting in an overall *increase* in differential identifiability, as shown in the main text. ***, $p < 0.001$ from paired-samples t-tests (two-sided). Box plots: the central lines indicate median values, the bounds of the boxes indicate the 25th and 75th percentiles, the whiskers indicate 1.5× the interquartile range. Each data-point represents one individual scan. Human: n=100 individuals; macaque: n=19 data-points from 10 animals; mouse: n=10 animals.

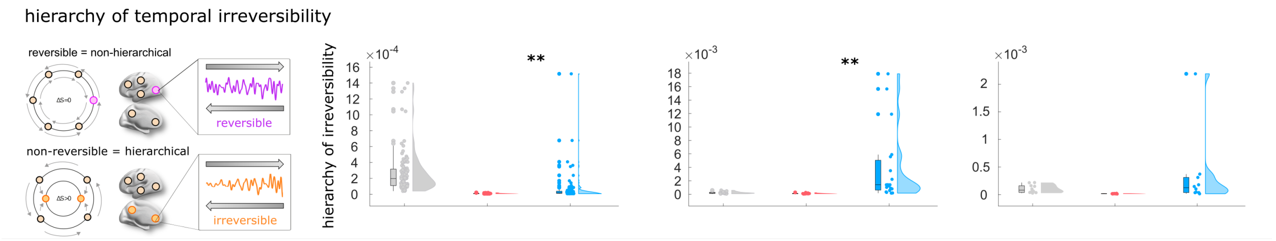

Figure S5. Greater hierarchy of temporal irreversibility is observed in the presence of competitive interactions. Level of hierarchy is given by the level of asymmetry of interactions between brain regions arising from the breaking of the detailed balance. A non-hierarchical system is depicted, being in full detailed balance and thus fully reversible over time. In contrast, asymmetry of the interactions results in a hierarchical organisation. **, $p < 0.01$ from paired-samples t-tests (two-sided). See Supplementary Tables 1-3 for full statistical reporting. Box plots: the central lines indicate median values, the bounds of the boxes indicate the 25th and 75th percentiles, the whiskers indicate 1.5× the interquartile range. Each data-point represents one individual scan. Human: n=100 individuals; macaque: n=19 data-points from 10 animals; mouse: n=10 animals.

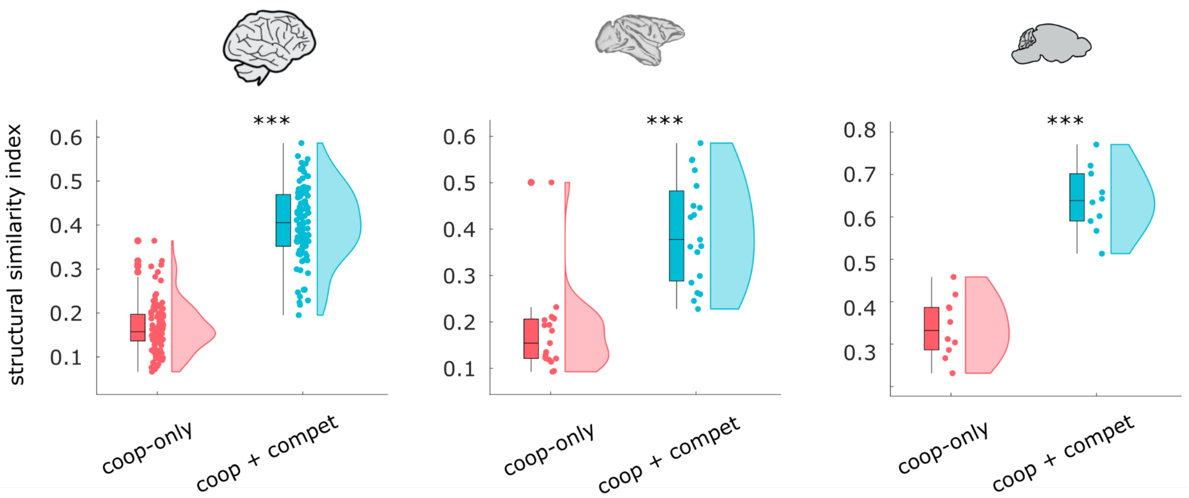

Figure S6. Alternative fitting measure with SSIM. In each species, the model with cooperative and competitive interactions achieves superior subject-level fit with the empirical FC, as measured by the structural similarity index (SSIM). ***, $p < 0.001$ from paired-samples t-tests (two-sided). Box plots: the central lines indicate median values, the bounds of the boxes indicate the 25th and 75th percentiles, the whiskers indicate 1.5× the interquartile range. Each data-point represents one individual scan. Human: n=100 individuals; macaque: n=19 data-points from 10 animals; mouse: n=10 animals. See Supplementary Tables 1-3 for full statistical reporting.

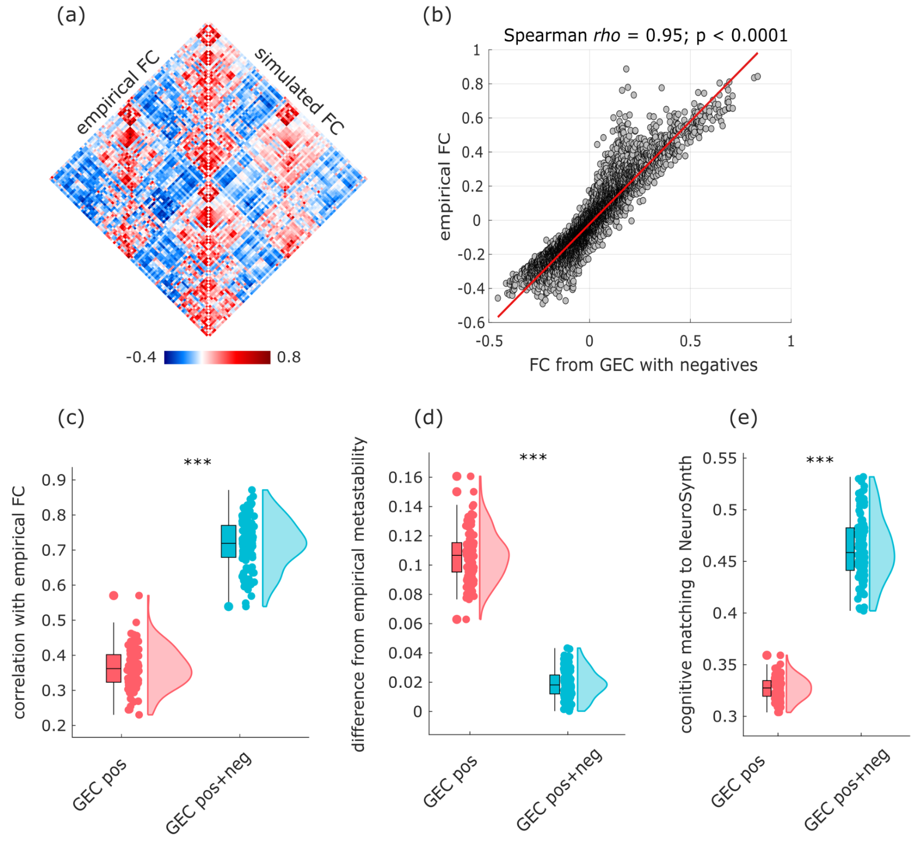

Figure S7. Replication of human results with global signal regression. (**a**) Group-wise empirical and simulated FC. (**b**) Correlation between group-wise empirical and simulated FC (Spearman *rho* = 0.95, p < 0.001; n=9,900 edges). (**c**) Subject-wise correlations between empirical and simulated FC are significantly higher for the model including both cooperative and competitive interactions in the generative connectivity. (**d**) Subject-wise difference between empirical and simulated metastability is significantly lower (reflecting greater similarity) for the model including both cooperative and competitive interactions in the generative connectivity. (**e**) Subject-wise cognitive matching to NeuroSynth meta-analytic maps is significantly higher for the model including both cooperative and competitive interactions in the generative connectivity. ***, p < 0.001 from paired-samples t-tests (two-sided). Box plots: the central lines indicate median values, the bounds of the boxes indicate the 25th and 75th percentiles, the whiskers indicate 1.5× the interquartile range. Each data-point represents one individual scan; n=100 individuals.

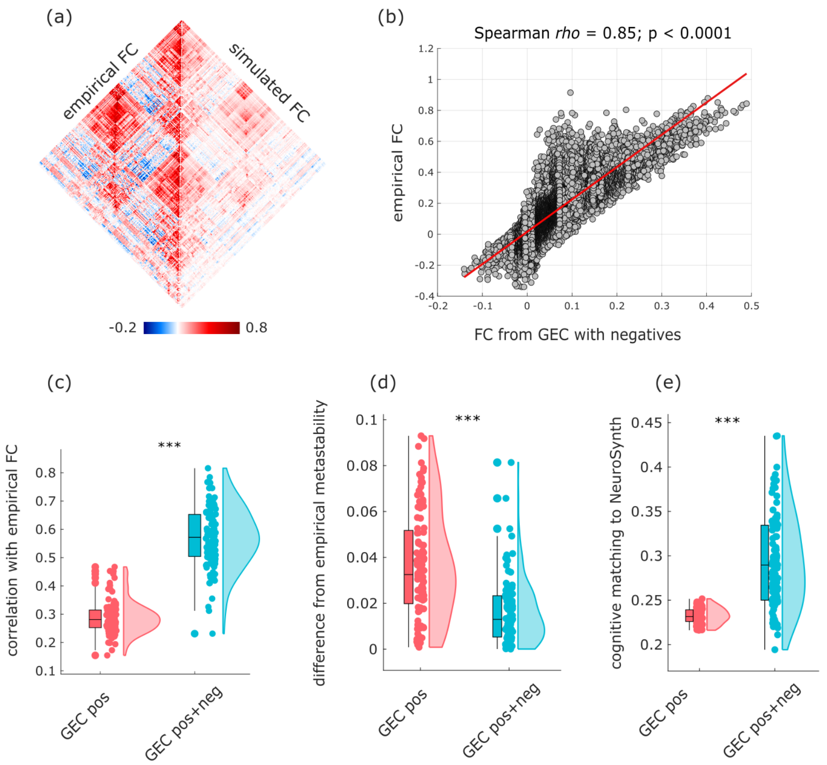

Figure S8. Replication of human results with a different cortical parcellation including subcortex (Schaefer-232). (**a**) Group-wise empirical and simulated FC. (**b**) Correlation between group-wise empirical and simulated FC (Spearman *rho* = 0.85, $p < 0.001$, n = 53,592 edges). (**c**) Subject-wise correlations between empirical and simulated FC are significantly higher for the model including both cooperative and competitive interactions in the generative connectivity. (**d**) Subject-wise difference between empirical and simulated metastability is significantly lower (reflecting greater similarity) for the model including both cooperative and competitive interactions in the generative connectivity. (**e**) Subject-wise cognitive matching to NeuroSynth meta-analytic maps is significantly higher for the model including both cooperative and competitive interactions in the generative connectivity. ***, $p < 0.001$ from paired-samples t-tests (two-sided). Box plots: the central lines indicate median values, the bounds of the boxes indicate the 25th and 75th percentiles, the whiskers indicate 1.5× the interquartile range. Each data-point represents one individual scan; n=100 individuals.

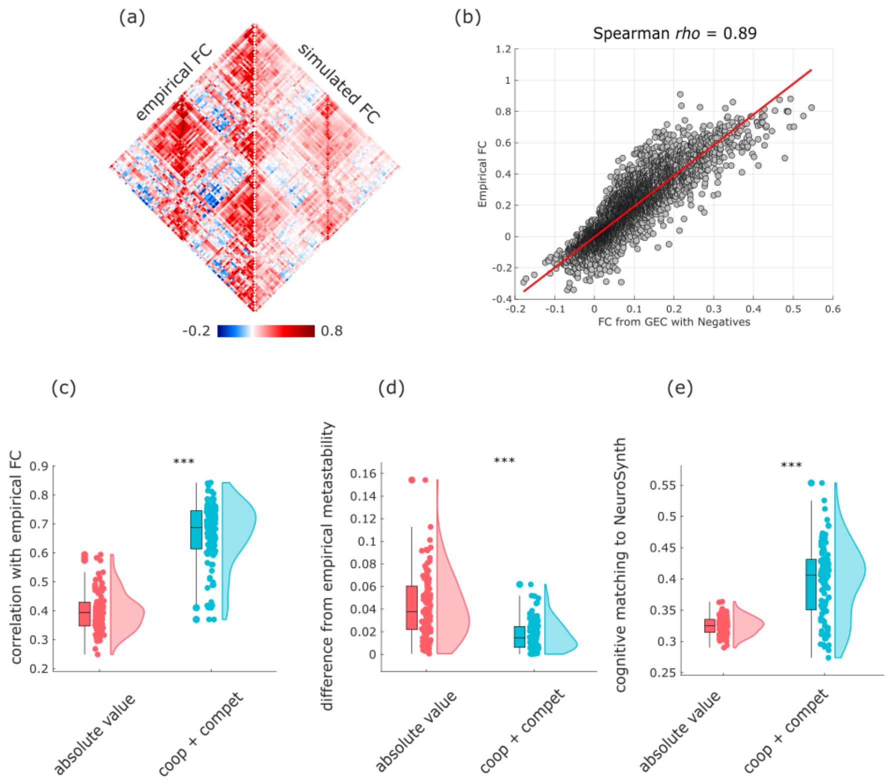

Figure S9. Replication of human results with homotopic connections added to the SC. (**a**) Group-wise empirical and simulated FC. (**b**) Correlation between group-wise empirical and simulated FC (Spearman *rho* = 0.89, p < 0.001, n = 9900 edges). (**c**) Subject-wise correlations between empirical and simulated FC are significantly higher for the model including both cooperative and competitive interactions in the generative connectivity. (**d**) Subject-wise difference between empirical and simulated metastability is significantly lower (reflecting greater similarity) for the model including both cooperative and competitive interactions in the generative connectivity. (**e**) Subject-wise cognitive matching to NeuroSynth meta-analytic maps is significantly higher for the model including both cooperative and competitive interactions in the generative connectivity. ***, p < 0.001 from paired-samples t-tests (two-sided). Box plots: the central lines indicate median values, the bounds of the boxes indicate the 25th and 75th percentiles, the whiskers indicate 1.5× the interquartile range. Each data-point represents one individual scan; n=100 individuals.

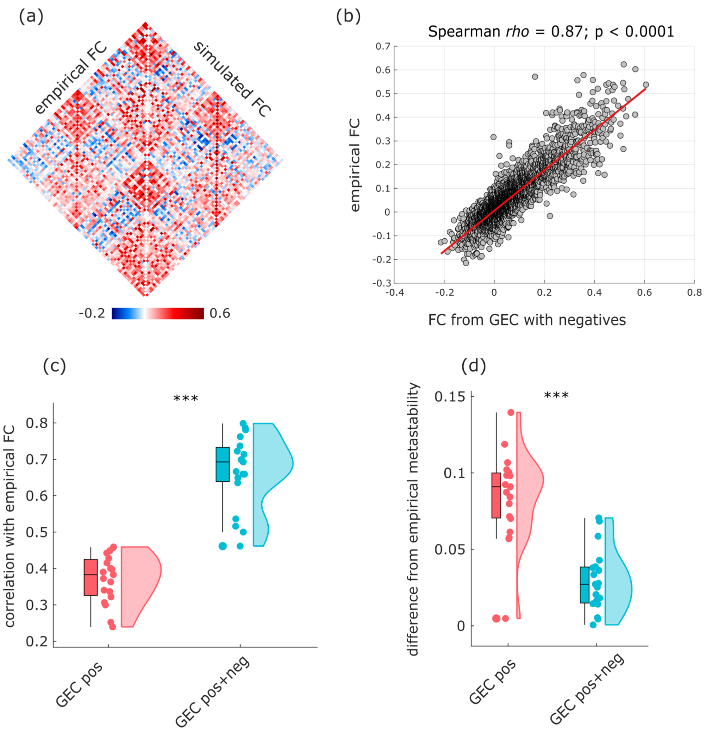

Figure S10. Replication of macaque results with asymmetric connectome. (**a**) Group-wise empirical and simulated FC. (**b**) Correlation between group-wise empirical and simulated FC (Spearman *rho* = 0.87, $p < 0.001$, n = 6642 edges). (**c**) Subject-wise correlations between empirical and simulated FC are significantly higher for the model including both cooperative and competitive interactions in the generative connectivity. (**d**) Subject-wise difference between empirical and simulated metastability is significantly lower (reflecting greater similarity) for the model including both cooperative and competitive interactions in the generative connectivity. ***, $p < 0.001$ from paired-samples t-tests (two-sided). Box plots: the central lines indicate median values, the bounds of the boxes indicate the 25th and 75th percentiles, the whiskers indicate 1.5× the interquartile range. Each data-point represents one individual scan; n=19 scans from 10 individual animals.

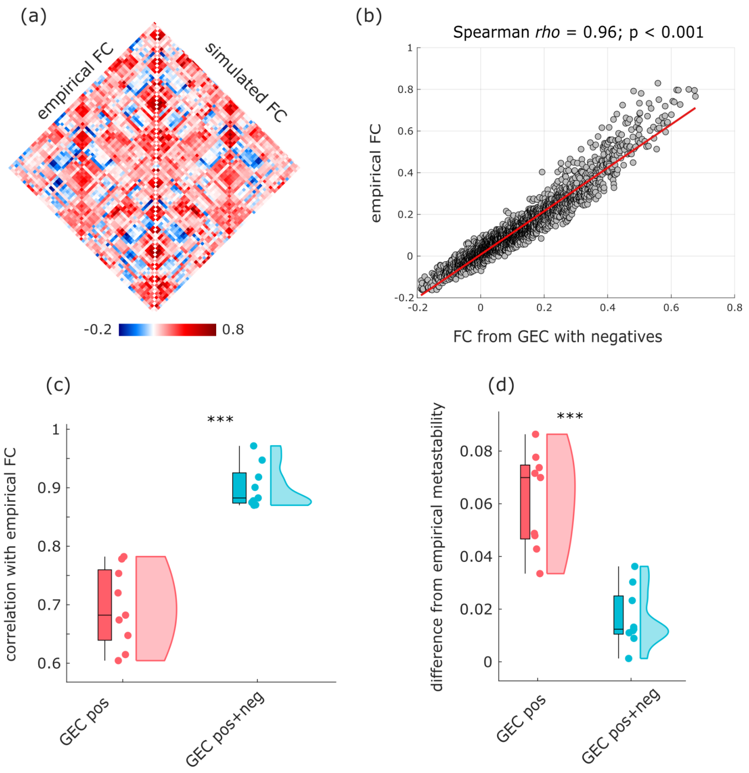

Figure S11. Replication of mouse results with asymmetric, fully dense connectome. (**a**) Group-wise empirical and simulated FC. (**b**) Correlation between group-wise empirical and simulated FC (Spearman *rho* = 0.96, $p < 0.001$, n = 5112 edges). (**c**) Subject-wise correlations between empirical and simulated FC are significantly higher for the model including both cooperative and competitive interactions in the generative connectivity. (**d**) Subject-wise difference between empirical and simulated metastability is significantly lower (reflecting greater similarity) for the model including both cooperative and competitive interactions in the generative connectivity. ***, $p < 0.001$ from paired-samples t-tests (two-sided). Box plots: the central lines indicate median values, the bounds of the boxes indicate the 25th and 75th percentiles, the whiskers indicate 1.5× the interquartile range. Each data-point represents one individual scan; n= 10 animals.

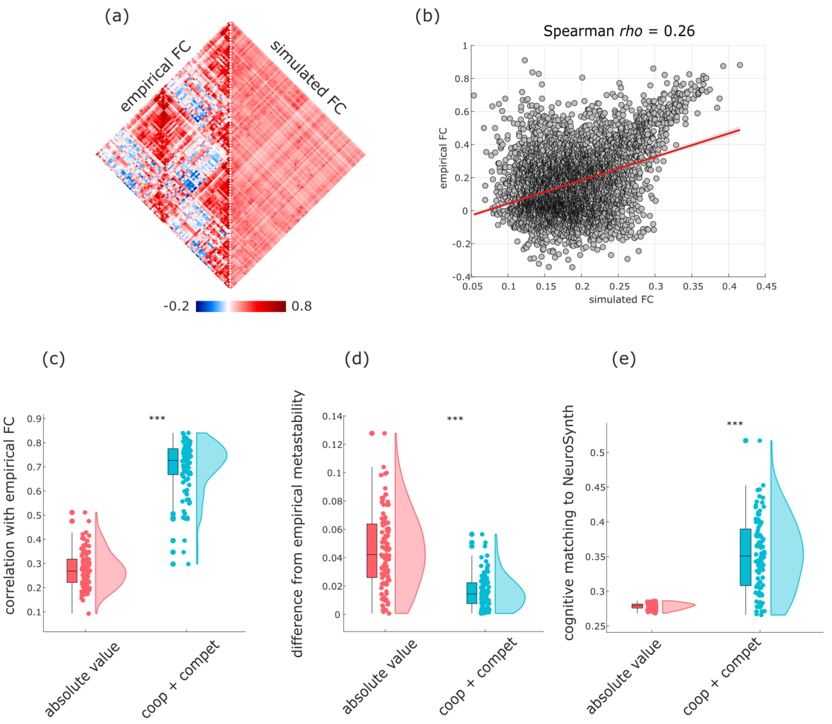

Figure S12. Turning competitive interactions into cooperative degrades performance of human model. (**a**) Group-wise empirical and simulated FC. (**b**) Correlation between group-wise empirical and simulated FC (Spearman *rho* = 0.26, n = 9900 edges). (**c**) Subject-wise correlations between empirical and simulated FC are significantly higher for the model including both cooperative and competitive interactions in the generative connectivity, than for the model where competitive interactions are turned into cooperative. (**d**) Subject-wise difference between empirical and simulated metastability is significantly lower (reflecting greater similarity) for the model including both cooperative and competitive interactions in the generative connectivity, than for the model where competitive interactions are turned into cooperative. (**e**) Subject-wise cognitive matching to NeuroSynth meta-analytic maps is significantly higher for the model including both cooperative and competitive interactions in the generative connectivity, than for the model where competitive interactions are turned into cooperative. ***, $p < 0.001$ from paired-samples t-tests (two-sided). Box plots: the central lines indicate median values, the bounds of the boxes indicate the 25th and 75th percentiles, the whiskers indicate 1.5× the interquartile range. Each data-point represents one individual scan; n=100 individuals.

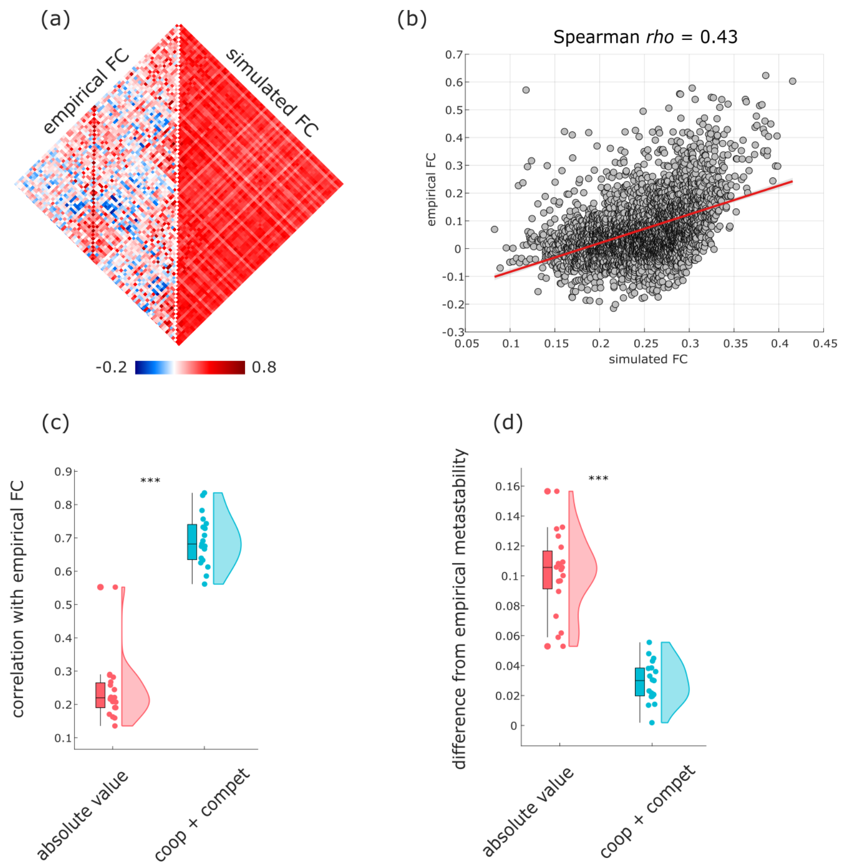

Figure S13. Turning competitive interactions into cooperative degrades performance of macaque model. (**a**) Group-wise empirical and simulated FC. (**b**) Correlation between group-wise empirical and simulated FC (Spearman *rho* = 0.43, n = 6642 edges). (**c**) Subject-wise correlations between empirical and simulated FC are significantly higher for the model including both cooperative and competitive interactions in the generative connectivity, than for the model where competitive interactions are turned into cooperative. (**d**) Subject-wise difference between empirical and simulated metastability is significantly lower (reflecting greater similarity) for the model including both cooperative and competitive interactions in the generative connectivity, than for the model where competitive interactions are turned into cooperative. ***, $p < 0.001$ from paired-samples t-tests (two-sided). Box plots: the central lines indicate median values, the bounds of the boxes indicate the 25th and 75th percentiles, the whiskers indicate 1.5× the interquartile range. Each data-point represents one individual scan; n=19 scans from 10 individual animals.

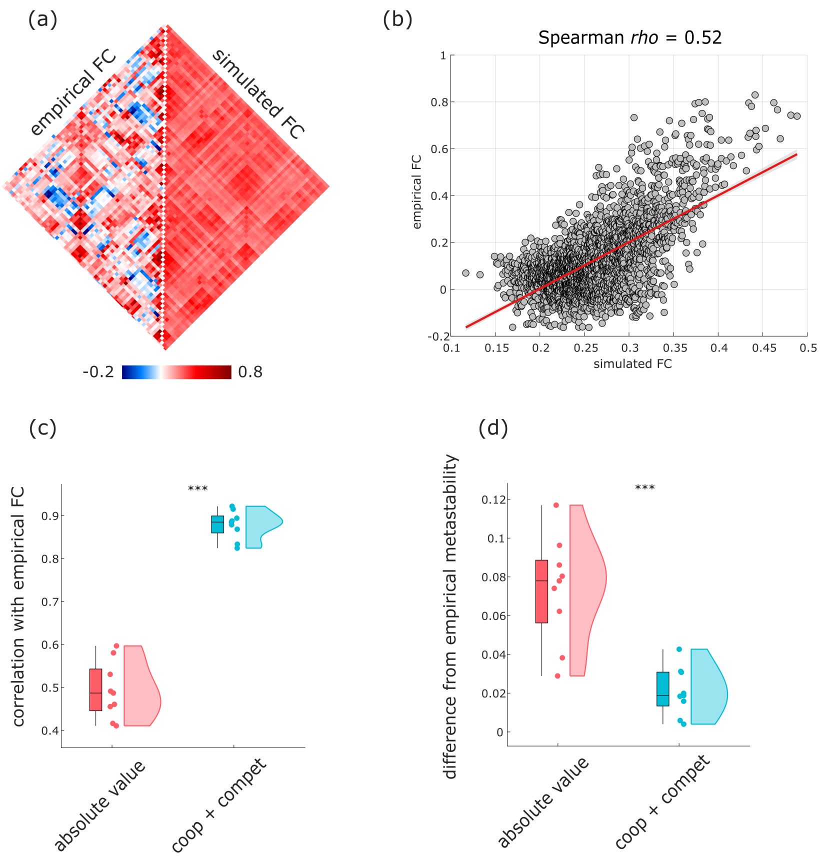

Figure S14. Turning competitive interactions into cooperative degrades performance of mouse model. (**a**) Group-wise empirical and simulated FC. (**b**) Correlation between group-wise empirical and simulated FC (Spearman *rho* = 0.52, n = 5112 edges). (**c**) Subject-wise correlations between empirical and simulated FC are significantly higher for the model including both cooperative and competitive interactions in the generative connectivity, than for the model where competitive interactions are turned into cooperative. (**d**) Subject-wise difference between empirical and simulated metastability is significantly lower (reflecting greater similarity) for the model including both cooperative and competitive interactions in the generative connectivity, than for the model where competitive interactions are turned into cooperative. ***, $p < 0.001$ from paired-samples t-tests (two-sided). Box plots: the central lines indicate median values, the bounds of the boxes indicate the 25th and 75th percentiles, the whiskers indicate 1.5× the interquartile range. Each data-point represents one individual scan; n= 10 animals.

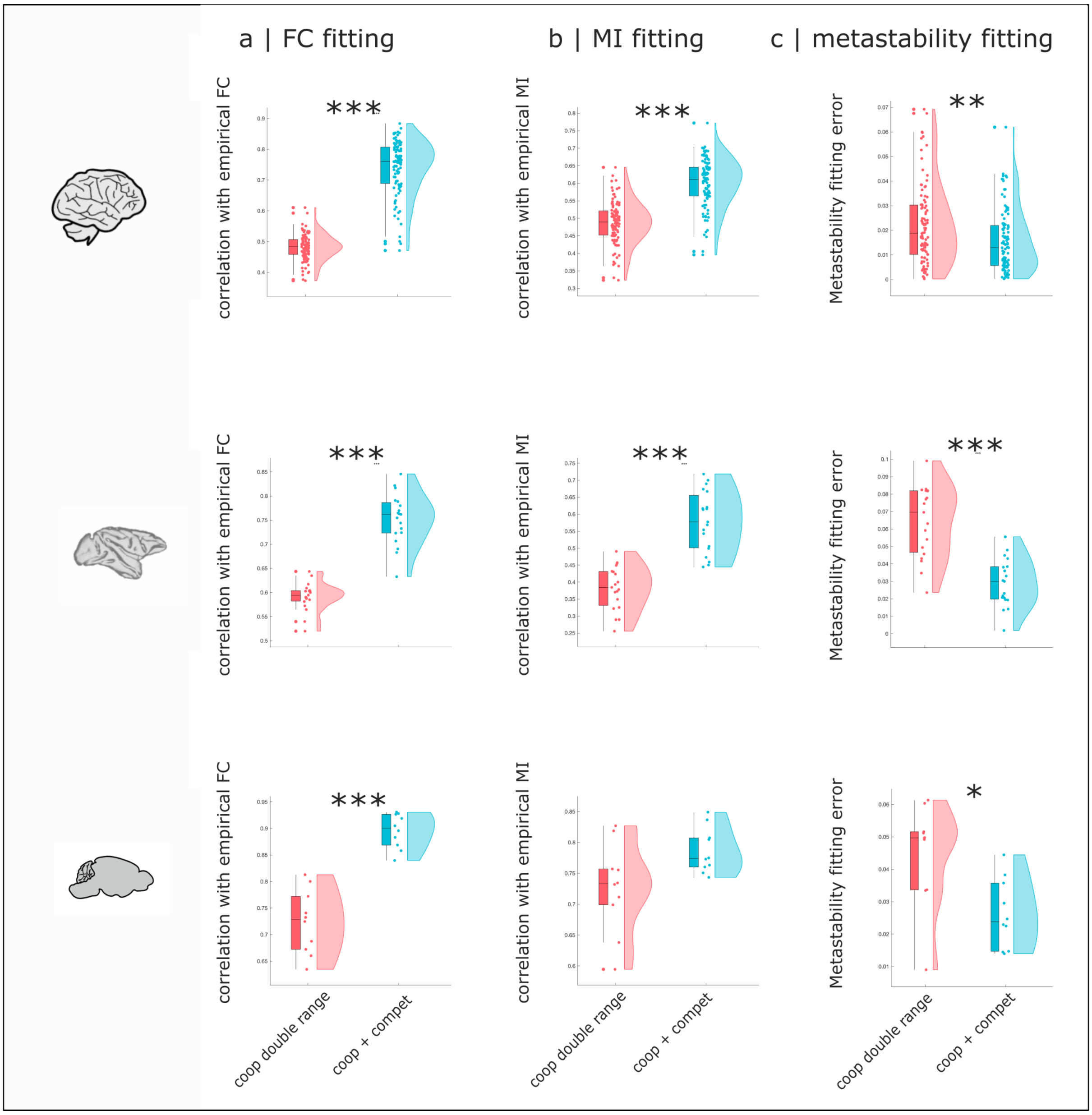

Figure S15. Generative competitive interactions lead to superior model fit beyond the role of increased weight range | (a) Correlation between empirical and simulated FC at the level of individual subjects is significantly higher for the model with competitive interactions. (**b**) Correlation between empirical and simulated mutual information (MI) at the level of individual subjects is significantly higher for the model with competitive interactions. Human: $p = 0.003$; Mouse: $p = 0.063$. (**c**) Difference between empirical and simulated metastability is significantly lower for the model with competitive interactions. Mouse: $p = 0.042$. (a-c) ** $p < 0.01$; ***, $p < 0.001$ from paired-samples t-tests (two-sided). Box plots: the central lines indicate median values, the bounds of the boxes indicate the 25th and 75th percentiles, the whiskers indicate 1.5× the interquartile range. Each data-point represents one individual scan. Human: n=100 individuals; macaque: n=19 data-points from 10 animals; mouse: n=10 animals.

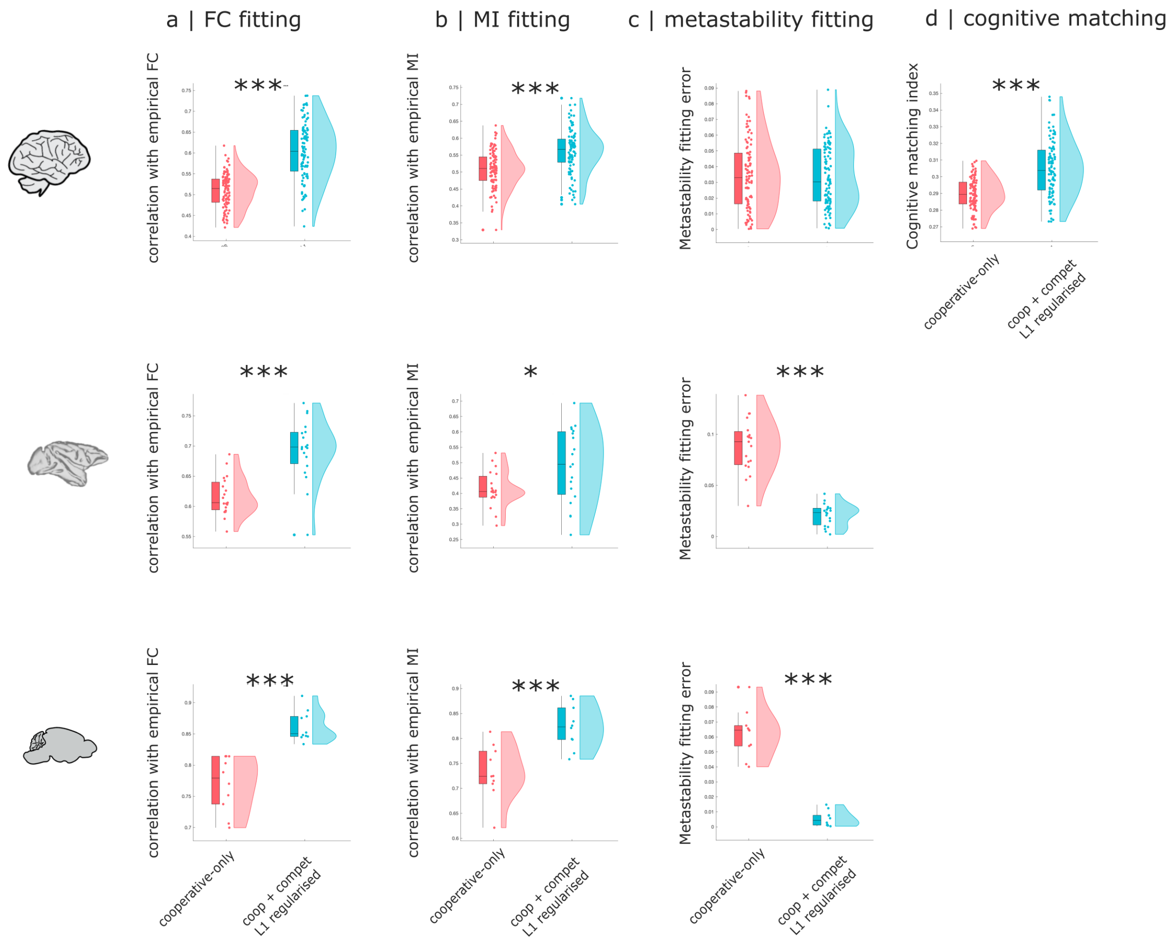

Figure S16. Generative competitive interactions lead to superior model fit even after L1 regularisation | (a) Correlation between empirical and simulated FC at the level of individual subjects is significantly higher for the L1-regularised model with competitive interactions. (**b**) Correlation between empirical and simulated mutual information (MI) at the level of individual subjects is significantly higher for the L1-regularised model with competitive interactions. Macaque: $p = 0.010$. (**c**) Difference between empirical and simulated metastability is significantly lower for the L1-regularised model with competitive interactions. Human: $p = 0.738$. (a-d) *, $p < 0.05$; ***, $p < 0.001$ from paired-samples t-tests (two-sided). Box plots: the central lines indicate median values, the bounds of the boxes indicate the 25th and 75th percentiles, the whiskers indicate 1.5× the interquartile range. Each data-point represents one individual scan. Human: n=100 individuals; macaque: n=19 data-points from 10 animals; mouse: n=10 animals.

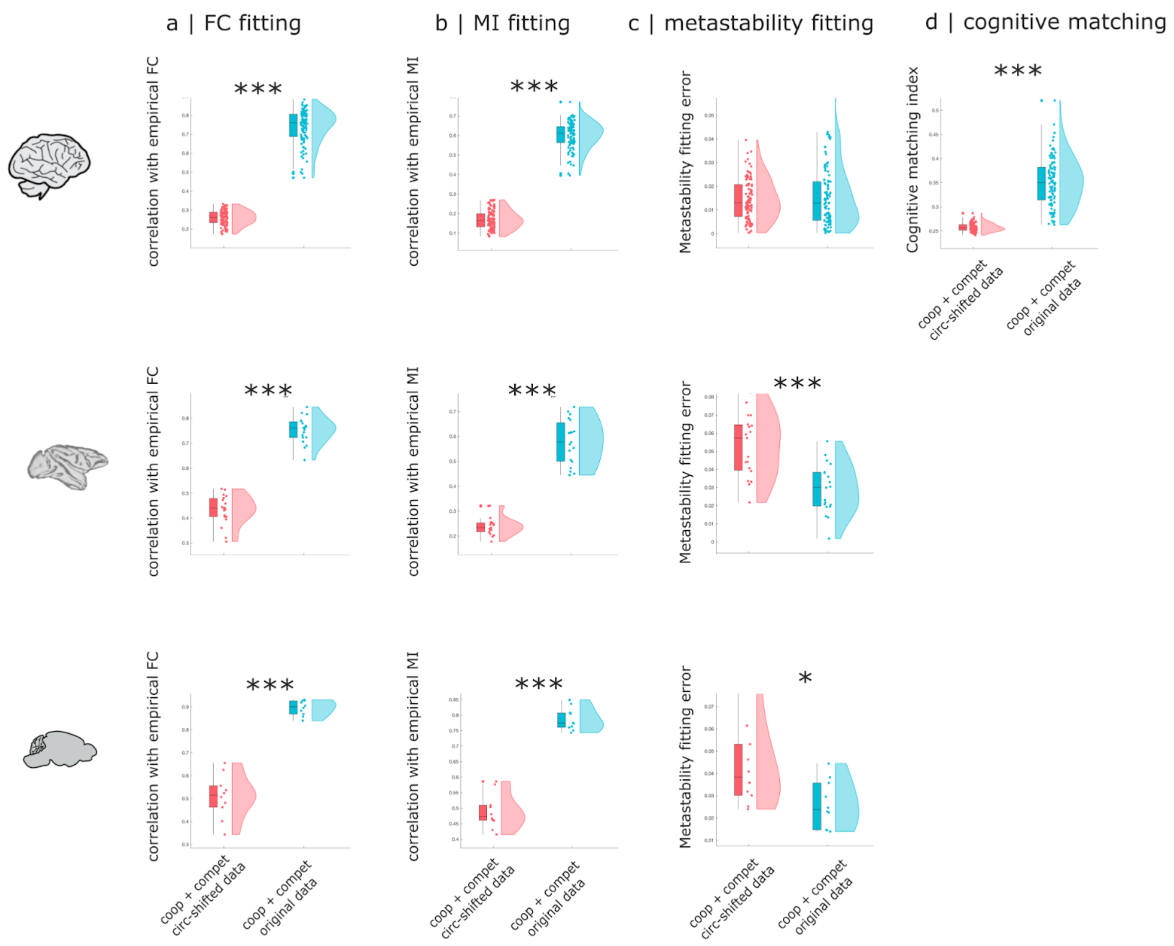

Figure S17. Superior performance of cooperative-competitive model trained on empirical than null (circular-shifted) data | (**a**) Correlation between empirical and simulated FC at the level of individual subjects is significantly higher when fitting empirical than null data. (**b**) Correlation between empirical and simulated mutual information (MI) at the level of individual subjects is significantly higher when fitting empirical than null data. (**c**) Difference between empirical and simulated metastability is significantly lower when fitting empirical than null data. Human: $p = 0.501$. Mouse: $p = 0.022$. (**d**) Cognitive matching from NeuroSynth at the level of individual subjects is significantly higher when fitting empirical than null data. (a-d) ***, $p < 0.001$ from paired-samples t-tests (two-sided). Box plots: the central lines indicate median values, the bounds of the boxes indicate the 25th and 75th percentiles, the whiskers indicate 1.5× the interquartile range. Each data-point represents one individual scan. Human: n=100 individuals; macaque: n=19 data-points from 10 animals; mouse: n=10 animals.

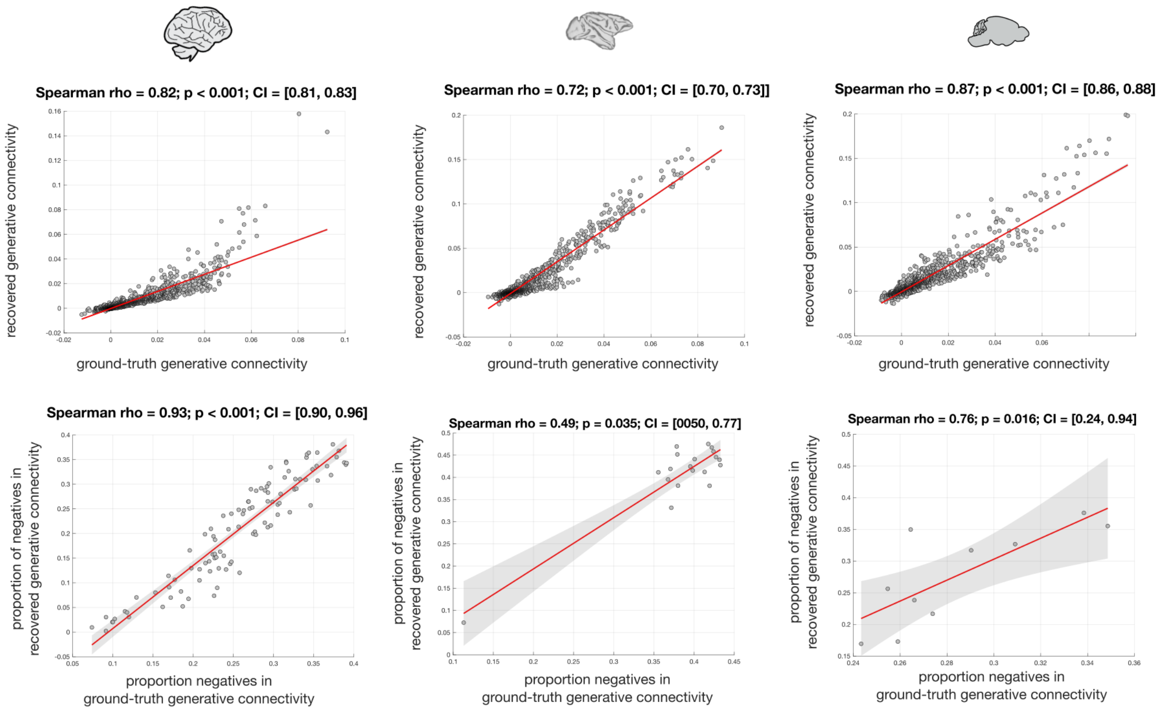

Figure S18. Cooperative-competitive model systematically recovers weight and sign of ground-truth generative connectivity | (top) Correlation between group-level ground-truth and inferred generative connectivity. (**bottom**) The proportion of negative weights in the inferred generative connectivity is highly correlated with the proportion of negative weights in the ground-truth generative connectivity. All correlations are from Spearman *rho,* two-sided. Shading indicates 95% confidence intervals (CI). Each data-point represents one individual scan. Human: n=100 individuals; macaque: n=19 data-points from 10 animals; mouse: n=10 animals.

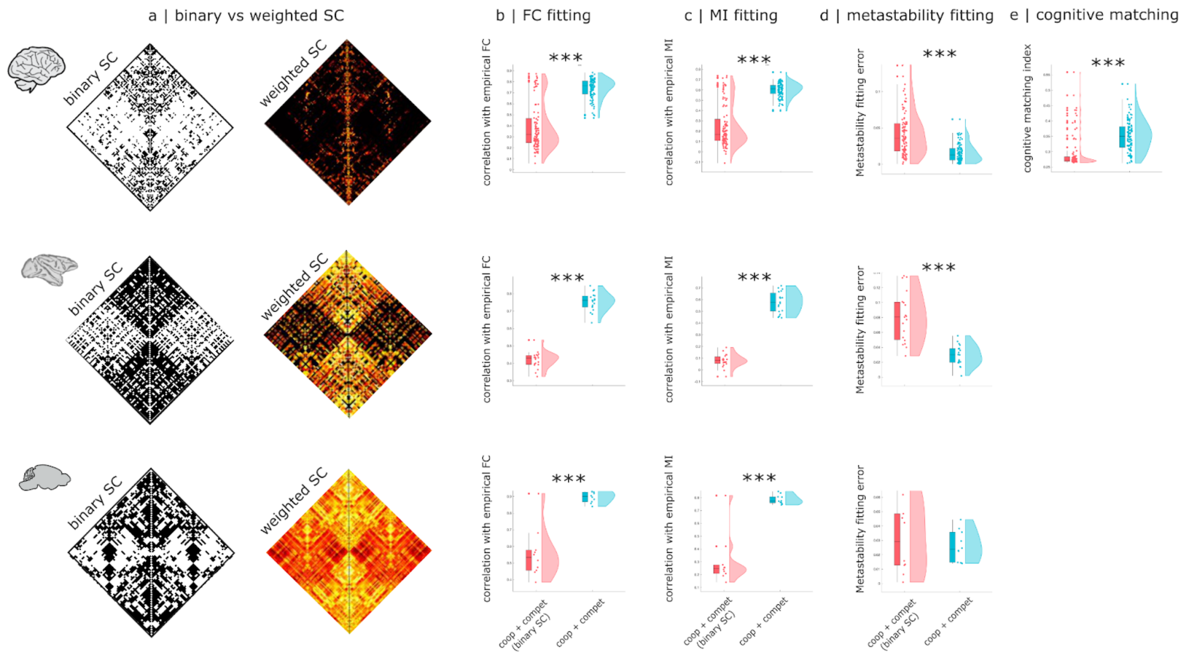

Figure S19. Model fit degrades if biological information about structural connection weights is removed by binarisation | (**a**) Binary vs weighted structural connectome used to initialise the cooperative-competitive model. (**b**) Correlation between empirical and simulated FC at the level of individual subjects is significantly higher for the model based on weighted than binary SC. (**c**) Correlation between empirical and simulated mutual information (MI) at the level of individual subjects is significantly higher for the model based on weighted than binary SC. (**d**) Difference between empirical and simulated metastability is significantly lower for the model based on weighted than binary SC. Mouse: $p = 0.479$. (**e**) Cognitive matching from NeuroSynth at the level of individual subjects is significantly higher for the model based on weighted than binary SC. (b-e) ***, $p < 0.001$ from paired-samples t-tests (two-sided). Box plots: the central lines indicate median values, the bounds of the boxes indicate the 25th and 75th percentiles, the whiskers indicate 1.5× the interquartile range. Each data-point represents one individual scan. Human: n=100 individuals; macaque: n=19 data-points from 10 animals; mouse: n=10 animals.

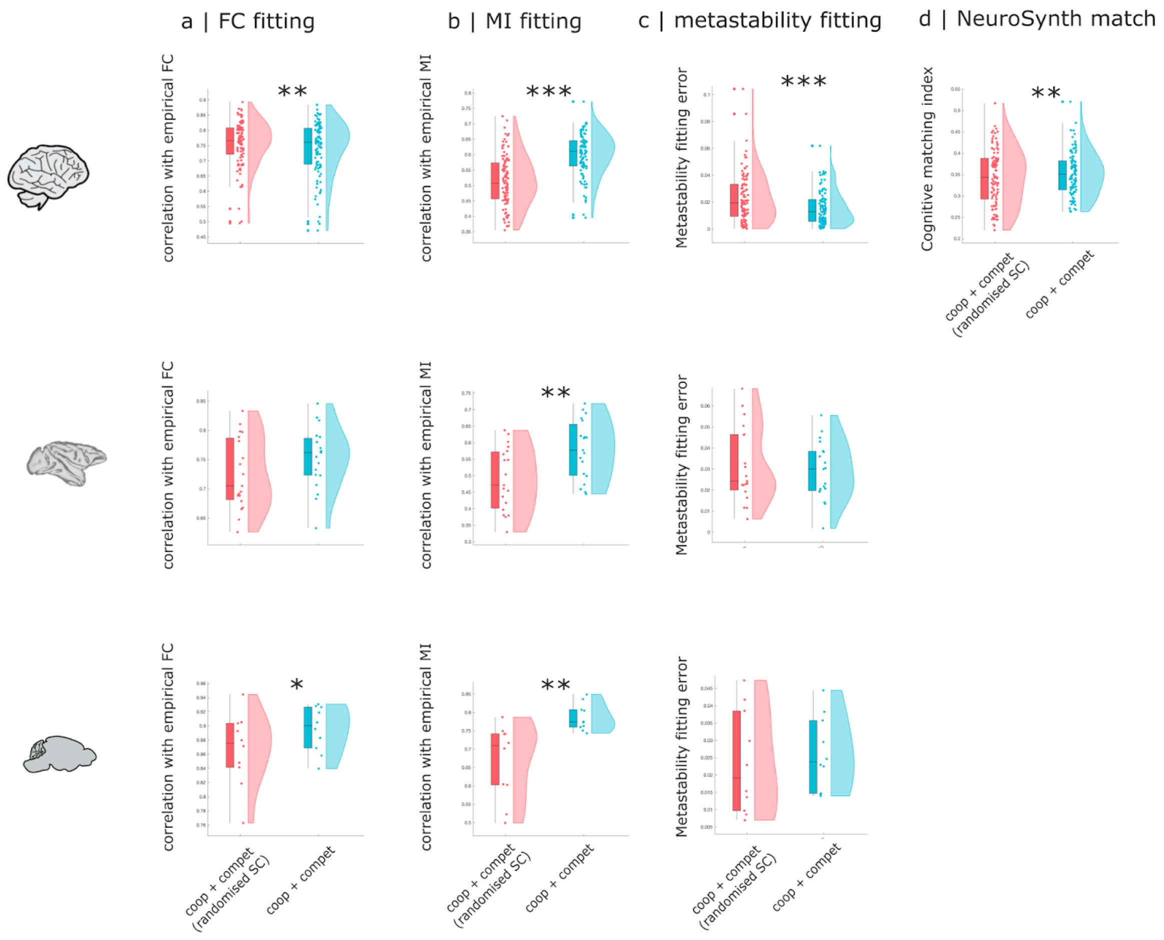

Figure S20. Fit of the cooperative-competitive model degrades if existing anatomical connections are replaced with non-existing connections of equal weight | (**a**) Correlation between empirical and simulated FC at the level of individual subjects for the empirical SC vs weight-preserving randomisation. Human: p = 0.001. Macaque: p = 0.082. Mouse: p = 0.026. (**b**) Correlation between empirical and simulated mutual information (MI) at the level of individual subjects for the empirical SC vs weight-preserving randomisation. Macaque: p = 0.002. Mouse: p = 0.002. (**c**) Difference between empirical and simulated metastability for the empirical SC vs weight-preserving randomisation. Macaque: p = 0.657. Mouse: p = 0.595. (**d**) Cognitive matching from NeuroSynth at the level of individual subjects for the empirical SC vs weight-preserving randomisation. Human: p = 0.008. (a-d) ***, p < 0.001 from paired-samples t-tests (two-sided). Box plots: the central lines indicate median values, the bounds of the boxes indicate the 25th and 75th percentiles, the whiskers indicate 1.5× the interquartile range. Each data-point represents one individual scan. Human: n=100 individuals; macaque: n=19 data-points from 10 animals; mouse: n=10 animals.

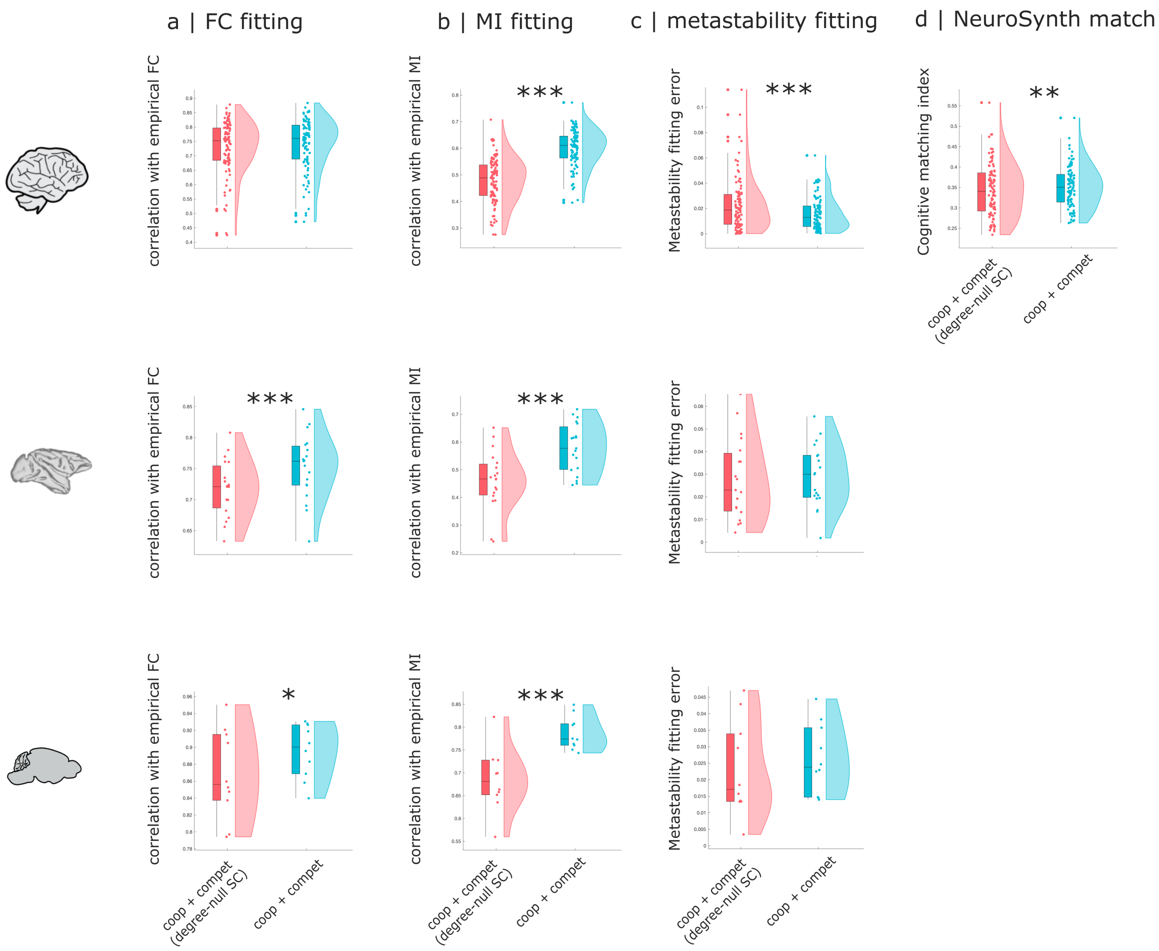

Figure S21. Fit of the cooperative-competitive model degrades if existing anatomical connections are replaced with non-existing connections while preserving degree sequence and weight distribution | (a) Correlation between empirical and simulated FC at the level of individual subjects for the empirical SC vs degree- and weight-preserving randomisation. Human: p = 0.059. Mouse: p = 0.031. (**b**) Correlation between empirical and simulated mutual information (MI) at the level of individual subjects for the empirical SC vs degree- and weight-preserving randomisation. (**c**) Difference between empirical and simulated metastability for the empirical SC vs degree- and weight-preserving randomisation. Macaque: p = 0.734. Mouse: p = 0.240. (**d**) Cognitive matching from NeuroSynth at the level of individual subjects for the empirical SC vs degree- and weight-preserving randomisation. Human: p = 0.003. (a-d) ***, p < 0.001 from paired-samples t-tests (two-sided). Box plots: the central lines indicate median values, the bounds of the boxes indicate the 25th and 75th percentiles, the whiskers indicate 1.5× the interquartile range. Each data-point represents one individual scan. Human: n=100 individuals; macaque: n=19 data-points from 10 animals; mouse: n=10 animals.

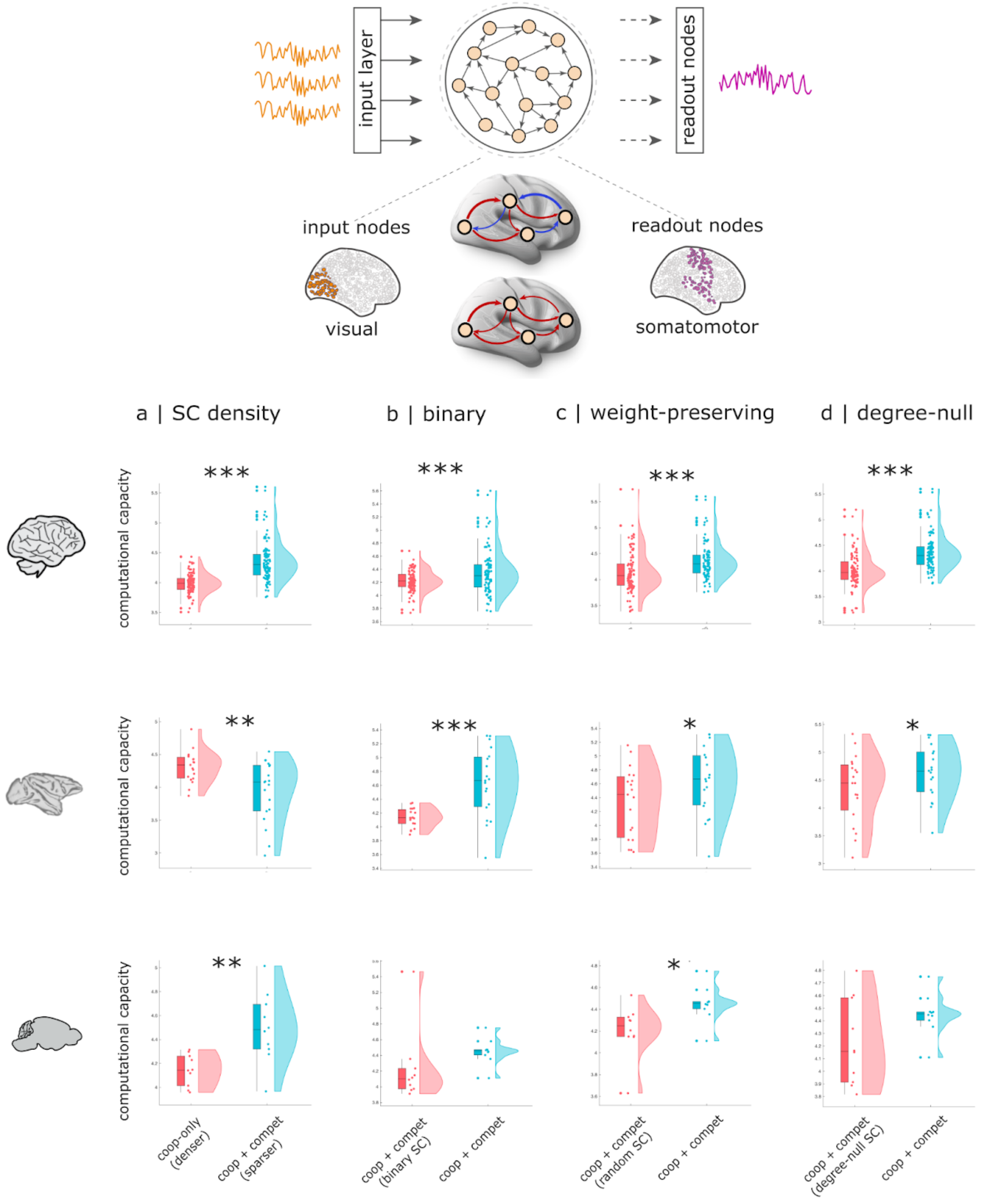

Figure S22. Computational task performance of the cooperative-competitive model vs alternative instantiations | (**a**) Comparison between the cooperative-competitive model, and the cooperative-only model having twice as many parameters (denser structural connectome). Macaque: p = 0.009. Mouse: p = 0.008. (**b**) Comparison between the cooperative-competitive model, and a cooperative-competitive model based on a binary SC that disregards biological information about connection weight. Mouse: p = 0.018. (**c**) Comparison between the cooperative-competitive model, and cooperative-competitive model based on randomised SC whereby existing anatomical connections are replaced with non-existing connections. Macaque: p = 0.011. Mouse: p = 0.036. (**d**) Comparison between the cooperative-competitive model, and cooperative-competitive model based on degree- and weight-preserving randomised SC. Macaque: p = 0.034. Mouse: p = 0.080. (a-d) ***, p < 0.001 from paired-samples t-tests (two-sided). Box plots: the central lines indicate median values, the bounds of the boxes indicate the 25th and 75th percentiles, the whiskers indicate 1.5× the interquartile range. Each data-point represents one individual scan. Human: n=100 individuals; macaque: n=19 data-points from 10 animals; mouse: n=10 animals.

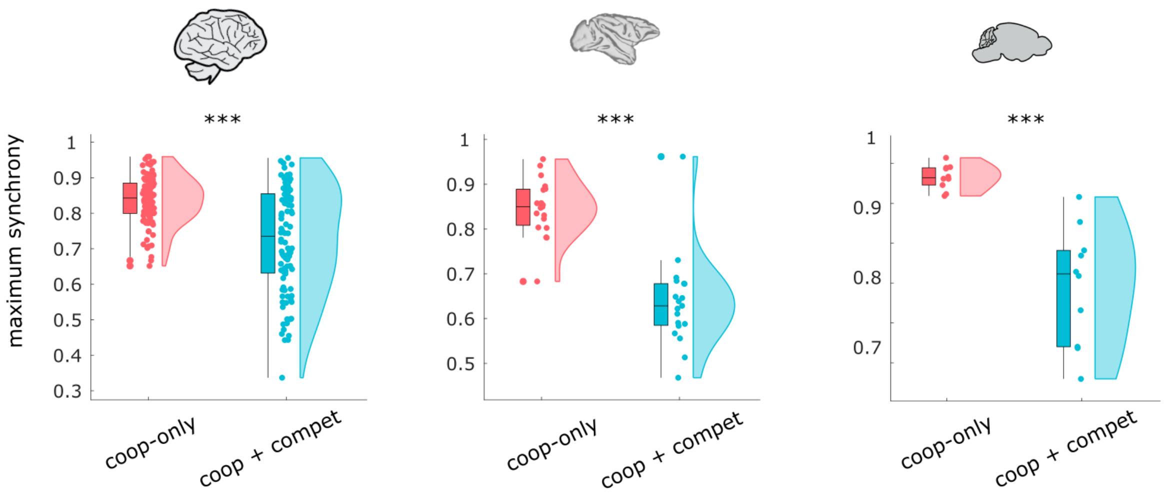

Figure S23. Maximum synchrony. Instantaneous synchrony is quantified by the Kuramoto order parameter of the narrowband signal; for each individual, we show its maximum observed value. The maximum synchrony is significantly higher in the model without competitive interactions. See Supplementary Tables 1-3 for full statistical reporting. ***, $p < 0.001$ from paired-samples t-tests (two-sided). Box plots: the central lines indicate median values, the bounds of the boxes indicate the 25th and 75th percentiles, the whiskers indicate 1.5× the interquartile range. Each data-point represents one individual scan. Human: n=100 individuals; macaque: n=19 data-points from 10 animals; mouse: n=10 animals.

action	adaptation	addiction	anticipation	anxiety
arousal	association	attention	autobiographical memory	balance
belief	categorization	cognitive control	communication	competition
concept	consciousness	consolidation	context	coordination
decision	decision making	detection	discrimination	distraction
eating	efficiency	effort	emotion	emotion regulation
empathy	encoding	episodic memory	expectancy	expertise
extinction	face recognition	facial expression	familiarity	fear
fixation	focus	gaze	goal	hyperactivity
imagery	impulsivity	induction	inference	inhibition
insight	integration	intelligence	intention	interference
judgment	knowledge	language	language comprehension	learning
listening	localization	loss	maintenance	manipulation
meaning	memory	memory retrieval	mental imagery	monitoring
mood	morphology	motor control	movement	multisensory
naming	navigation	object recognition	pain	perception
planning	priming	psychosis	reading	reasoning
recall	recognition	rehearsal	reinforcement learning	response inhibition
response selection	retention	retrieval	reward anticipation	rhythm
risk	rule	salience	search	selective attention
semantic memory	sentence comprehension	skill	sleep	social cognition
spatial attention	speech perception	speech production	strategy	strength
stress	sustained attention	task difficulty	thought	uncertainty
updating	utility	valence	verbal fluency	visual attention
visual perception	word recognition	working memory		

Figure S24. NeuroSynth terms. | Terms that overlapped between the NeuroSynth database [125] and the Cognitive Atlas [126] were included in the analysis.

Supplementary References

1. Perl, Y. S. *et al.* The impact of regional heterogeneity in whole-brain dynamics in the presence of oscillations. *Network Neuroscience* 1–29 (2023) doi:10.1162/netn_a_00299.
2. Piccinini, J. *et al.* Data-driven discovery of canonical large-scale brain dynamics. *Cerebral Cortex Communications* **3**, tgac045 (2022).
3. Sip, V. *et al.* Characterization of regional differences in resting-state fMRI with a data-driven network model of brain dynamics. *Science Advances* **9**, eabq7547 (2023).
4. Schirner, M., Deco, G. & Ritter, P. Learning how network structure shapes decision-making for bio-inspired computing. *Nat Commun* **14**, 2963 (2023).
5. Whittington, M. A., Traub, R. D., Kopell, N., Ermentrout, B. & Buhl, E. H. Inhibition-based rhythms: experimental and mathematical observations on network dynamics. *Int J Psychophysiol* **38**, 315–336 (2000).
6. Perkel, D. H. & Mulloney, B. Motor pattern production in reciprocally inhibitory neurons exhibiting postinhibitory rebound. *Science* **185**, 181–183 (1974).
7. Chen, X. *et al.* Regional GABA Concentrations Modulate Inter- network Resting-state Functional Connectivity. *Cerebral Cortex* 1–12 (2018) doi:10.1093/cercor/bhy059.
8. Gu, H., Hu, Y., Chen, X., He, Y. & Yang, Y. Regional excitation-inhibition balance predicts default-mode network deactivation via functional connectivity. *NeuroImage* **185**, 388–397 (2019).
9. Cabral, J., Kringelbach, M. L. & Deco, G. Functional connectivity dynamically evolves on multiple time-scales over a static structural connectome: Models and mechanisms. *NeuroImage* **160**, 84–96 (2017).
10. Kringelbach, M. L. & Deco, G. Brain States and Transitions: Insights from Computational Neuroscience. *Cell Reports* **32**, 108128 (2020).

11. Shine, J. M. *et al.* Computational models link cellular mechanisms of neuromodulation to large-scale neural dynamics. *Nature Neuroscience* **24**, 1–12 (2021).

12. Chen, A. *et al.* Single-cell spatial transcriptome reveals cell-type organization in the macaque cortex. *Cell* **186**, 3726-3743.e24 (2023).

13. Bansal, K. *et al.* Cognitive chimera states in human brain networks. *Science Advances* **5**, eaau8535 (2019).

14. Demirtaş, M. *et al.* Hierarchical Heterogeneity across Human Cortex Shapes Large-Scale Neural Dynamics. *Neuron* **101**, 1181-1194.e13 (2019).

15. Deco, G. *et al.* Whole-Brain Multimodal Neuroimaging Model Using Serotonin Receptor Maps Explains Non-linear Functional Effects of LSD. *Current Biology* **28**, 3065–3074 (2018).

16. Pope, M., Fukushima, M., Betzel, R. F. & Sporns, O. Modular origins of high-amplitude cofluctuations in fine-scale functional connectivity dynamics. *Proceedings of the National Academy of Sciences of the United States of America* **118**, e2109380118 (2021).

17. Váša, F. *et al.* Effects of lesions on synchrony and metastability in cortical networks. *NeuroImage* **118**, 456–467 (2015).

18. Coronel-Oliveros, C., Gießing, C., Medel, V., Cofré, R. & Orio, P. Whole-brain modeling explains the context-dependent effects of cholinergic neuromodulation. *NeuroImage* **265**, 119782 (2023).

19. Pang, J. C., Rilling, J. K., Roberts, J. A., van den Heuvel, M. P. & Cocchi, L. Evolutionary shaping of human brain dynamics. *eLife* **11**, e80627 (2022).

20. Stramaglia, S. *et al.* Ising model with conserved magnetization on the human connectome: Implications on the relation structure-function in wakefulness and anesthesia. *Chaos* **27**, (2017).

21. Chaudhuri, R., Knoblauch, K., Gariel, M. A., Kennedy, H. & Wang, X. J. A Large-Scale Circuit Mechanism for Hierarchical Dynamical Processing in the Primate Cortex. *Neuron* **88**, 419–431 (2015).

22. Momi, D. *et al.* Stimulation mapping and whole-brain modeling reveal gradients of excitability and recurrence in cortical networks. *Nat Commun* **16**, 3222 (2025).

23. Gilson, M., Moreno-Bote, R., Ponce-Alvarez, A., Ritter, P. & Deco, G. Estimation of Directed Effective Connectivity from fMRI Functional Connectivity Hints at Asymmetries of Cortical Connectome. *PLOS Computational Biology* **12**, e1004762 (2016).

24. Pulvermüller, F., Tomasello, R., Henningsen-Schomers, M. R. & Wennekers, T. Biological constraints on neural network models of cognitive function. *Nature Reviews Neuroscience* https://doi.org/10.1038/s41583-021-00473-5 (2021) doi:10.1038/s41583-021-00473-5.

25. Froudist-Walsh, S. *et al.* A dopamine gradient controls access to distributed working memory in the large-scale monkey cortex. *Neuron* **109**, 3500-3520.e13 (2021).

26. Joglekar, M. R., Mejias, J. F., Yang, G. R. & Wang, X. J. Inter-areal Balanced Amplification Enhances Signal Propagation in a Large-Scale Circuit Model of the Primate Cortex. *Neuron* **98**, 222-234.e8 (2018).

27. Greaves, M. D., Novelli, L., Mansour L., S., Zalesky, A. & Razi, A. Structurally informed models of directed brain connectivity. *Nat. Rev. Neurosci.* 1–19 (2024) doi:10.1038/s41583-024-00881-3.

28. Friston, K. J., Harrison, L. & Penny, W. Dynamic causal modelling. *NeuroImage* **19**, 1273–1302 (2003).

29. Friston, K. J. *et al.* Dynamic causal modelling revisited. *NeuroImage* **199**, 730–744 (2019).

30. Valdes-Sosa, P. A., Roebroeck, A., Daunizeau, J. & Friston, K. Effective connectivity: Influence, causality and biophysical modeling. *NeuroImage* **58**, 339–361 (2011).
31. Reid, A. T. *et al.* Advancing functional connectivity research from association to causation. *Nature Neuroscience* **22**, 1751–1760 (2019).
32. Friston, K. J., Mechelli, A., Turner, R. & Price, C. J. Nonlinear Responses in fMRI: The Balloon Model, Volterra Kernels, and Other Hemodynamics. *NeuroImage* **12**, 466–477 (2000).
33. Frässle, S. *et al.* A generative model of whole-brain effective connectivity. *NeuroImage* **179**, 505–529 (2018).
34. Sokolov, A. A. *et al.* Structural and effective brain connectivity underlying biological motion detection. *Proc Natl Acad Sci U S A* **115**, E12034–E12042 (2018).
35. Deco, G. *et al.* Different hierarchical reconfigurations in the brain by psilocybin and escitalopram for depression. *Nat. Mental Health* 1–15 (2024) doi:10.1038/s44220-024-00298-y.
36. Kringelbach, M. L., Perl, Y. S., Tagliazucchi, E. & Deco, G. Toward naturalistic neuroscience: Mechanisms underlying the flattening of brain hierarchy in movie-watching compared to rest and task. *Science Advances* **9**, eade6049 (2023).
37. Allegra, M., Gilson, M. & Brovelli, A. Directed neural interactions in fMRI: a comparison between Granger Causality and Effective Connectivity. 2024.02.22.581068 Preprint at https://doi.org/10.1101/2024.02.22.581068 (2024).
38. Tanner, J. *et al.* A multi-modal, asymmetric, weighted, and signed description of anatomical connectivity. *Nat Commun* **15**, 5865 (2024).
39. Ruffini, G. *et al.* LSD-induced increase of Ising temperature and algorithmic complexity of brain dynamics. *PLOS Computational Biology* **19**, e1010811 (2023).

40. Swanson, L. W., Hahn, J. D. & Sporns, O. Organizing principles for the cerebral cortex network of commissural and association connections. *Proceedings of the National Academy of Sciences* **114**, E9692–E9701 (2017).
41. Calamante, F. The Seven Deadly Sins of Measuring Brain Structural Connectivity Using Diffusion MRI Streamlines Fibre-Tracking. *Diagnostics* **9**, 115 (2019).
42. Jeurissen, B., Descoteaux, M., Mori, S. & Leemans, A. Diffusion MRI fiber tractography of the brain. *NMR in Biomedicine* e3785 (2017) doi:10.1002/nbm.3785.
43. Sotiropoulos, S. N. & Zalesky, A. Building connectomes using diffusion MRI: why, how and but. *NMR Biomed* **32**, e3752 (2019).
44. Yeh, C. H., Smith, R. E., Liang, X., Calamante, F. & Connelly, A. Correction for diffusion MRI fibre tracking biases: The consequences for structural connectomic metrics. *NeuroImage* **142**, 150–162 (2016).
45. Cover, T. M. & Thomas, J. A. *Elements of Information Theory*. *Elements of Information Theory* (Wiley-Interscience, 2005). doi:10.1002/047174882X.
46. Williams, P. L. & Beer, R. D. Nonnegative Decomposition of Multivariate Information. *arXiv* https://doi.org/10.48550/arXiv.1004.2515 (2010) doi:https://doi.org/10.48550/arXiv.1004.2515.
47. Mediano, P. A. M. *et al.* Toward a unified taxonomy of information dynamics via Integrated Information Decomposition. *Proceedings of the National Academy of Sciences* **122**, e2423297122 (2025).
48. Luppi, A. I. *et al.* A Synergistic Workspace for Human Consciousness Revealed by Integrated Information Decomposition. *eLife* **12:RP88173**, 2020.11.25.398081 (2023).
49. Van Essen, D. C. *et al.* The WU-Minn Human Connectome Project: An overview. *NeuroImage* **80**, 62–79 (2013).

50. Glasser, M. F. *et al.* The Minimal Preprocessing Pipelines for the Human Connectome Project. *Neuroimage* **80**, 105–124 (2013).
51. Whitfield-Gabrieli, S. & Nieto-Castanon, A. Conn: A Functional Connectivity Toolbox for Correlated and Anticorrelated Brain Networks. *Brain Connectivity* **2**, 125–141 (2012).
52. Behzadi Y, Restom K, Liau J, & Liu TT. A component based noise correction method (CompCor) for BOLD and perfusion based fMRI. *NeuroImage* **37**, 90–101 (2007).
53. Schaefer, A. *et al.* Local-Global Parcellation of the Human Cerebral Cortex from Intrinsic Functional Connectivity MRI. *Cerebral Cortex* **28**, 3095–3114 (2018).
54. Tian, Y., Margulies, D., Breakspear, M. & Zalesky, A. Topographic organization of the human subcortex unveiled with functional connectivity gradients. *Nature Neuroscience* **23**, 1421–1432 (2020).
55. Michael Milham, A. P. *et al.* An Open Resource for Non-human Primate Imaging. *Neuron* **100**, 61-74.e2 (2018).
56. Luppi, A. I. *et al.* A synergistic core for human brain evolution and cognition. *Nat Neurosci* **25**, 771–782 (2022).
57. Tasserie, J. *et al.* Pypreclin: An automatic pipeline for macaque functional MRI preprocessing. *NeuroImage* **207**, (2020).
58. Seidlitz, J. *et al.* A population MRI brain template and analysis tools for the macaque. *NeuroImage* **170**, 121–131 (2018).
59. Kötter, R. & Wanke, E. Mapping brains without coordinates. *Philosophical Transactions of the Royal Society B: Biological Sciences* **360**, 751–766 (2005).
60. Gutierrez-Barragan, D. *et al.* Unique spatiotemporal fMRI dynamics in the awake mouse brain. *Current biology* **32**, 1–14 (2021).

61. Yeh, F.-C. *et al.* Population-averaged atlas of the macroscale human structural connectome and its network topology. *NeuroImage* **178**, 57–68 (2018).

62. Yeh, F.-C., Wedeen, V. J. & Tseng, W.-Y. I. Estimation of fiber orientation and spin density distribution by diffusion deconvolution. *NeuroImage* **55**, 1054–1062 (2011).

63. Luppi, A. I. & Stamatakis, E. A. Combining network topology and information theory to construct representative brain networks. *Network Neuroscience* **5**, 96–124 (2021).

64. Shen, K. *et al.* A macaque connectome for large-scale network simulations in TheVirtualBrain. *Scientific Data* **6**, (2019).

65. Bakker, R., Wachtler, T. & Diesmann, M. CoCoMac 2.0 and the future of tract-tracing databases. *Front. Neuroinform.* **6**, (2012).

66. Luppi, A. I. *et al.* Local orchestration of distributed functional patterns supporting loss and restoration of consciousness in the primate brain. *Nat Commun* **15**, 2171 (2024).

67. Coletta, L. *et al.* Network structure of the mouse brain connectome with voxel resolution. *Science Advances* **6**, 7187–7205 (2020).

68. Knox, J. E. *et al.* High-resolution data-driven model of the mouse connectome. *Network Neuroscience* **3**, 217–236 (2018).

69. Hawrylycz, M. J. *et al.* An anatomically comprehensive atlas of the adult human brain transcriptome. *Nature* **489**, 391–399 (2012).

70. Zarkali, A. *et al.* Changes in dynamic transitions between integrated and segregated states underlie visual hallucinations in Parkinson's disease. *Commun Biol* **5**, 1–15 (2022).

71. Markello, R. D. *et al.* Standardizing workflows in imaging transcriptomics with the Abagen toolbox. *eLife* **10**, (2021).

72. Luppi, A. I. *et al.* Benchmarking macaque brain gene expression for horizontal and vertical translation. 2024.08.18.608440 Preprint at https://doi.org/10.1101/2024.08.18.608440 (2024).

73. Lein, E. S. *et al.* Genome-wide atlas of gene expression in the adult mouse brain. *Nature* **445**, 168–176 (2007).

74. Yee, Y. *et al.* Structural covariance of brain region volumes is associated with both structural connectivity and transcriptomic similarity. *NeuroImage* **179**, 357–372 (2018).

75. Wang, Q. *et al.* The Allen Mouse Brain Common Coordinate Framework: A 3D Reference Atlas. *Cell* **181**, 936-953.e20 (2020).

76. Fulcher, B. D., Murray, J. D., Zerbi, V. & Wang, X.-J. Multimodal gradients across mouse cortex. *Proceedings of the National Academy of Sciences* **116**, 4689–4695 (2019).

77. Luppi, A. I. *et al.* Benchmarking macaque brain gene expression for horizontal and vertical translation. *Science Advances* **11**, eads6967 (2025).

78. Kim, Y. *et al.* Brain-wide Maps Reveal Stereotyped Cell-Type-Based Cortical Architecture and Subcortical Sexual Dimorphism. *Cell* **171**, 456-469.e22 (2017).

79. Burt, J. B. *et al.* Hierarchy of transcriptomic specialization across human cortex captured by structural neuroimaging topography. *Nature Neuroscience* **21**, 1251–1259 (2018).

80. Huntenburg, J. M., Bazin, P.-L. & Margulies, D. S. Large-Scale Gradients in Human Cortical Organization. *Trends Cogn Sci* **22**, 21–31 (2018).

81. Markello, R. D. *et al.* neuromaps: structural and functional interpretation of brain maps. *Nat Methods* **19**, 1472–1479 (2022).

82. Froudist-Walsh, S. *et al.* Gradients of neurotransmitter receptor expression in the macaque cortex. *Nat Neurosci* https://doi.org/10.1038/s41593-023-01351-2 (2023) doi:10.1038/s41593-023-01351-2.

83. Zhang, X.-H. *et al.* The cell-type underpinnings of the human functional cortical connectome. *Nat Neurosci* **28**, 150–160 (2025).

84. Jorstad, N. L. *et al.* Comparative transcriptomics reveals human-specific cortical features. *Science* **382**, eade9516 (2023).

85. Erö, C., Gewaltig, M.-O., Keller, D. & Markram, H. A Cell Atlas for the Mouse Brain. *Frontiers in Neuroinformatics* **12**, (2018).

86. Hansen, J. Y. *et al.* Mapping neurotransmitter systems to the structural and functional organization of the human neocortex. *Nature Neuroscience* **25**, 1569–1581 (2022).

87. Kaller, S. *et al.* Test–retest measurements of dopamine D1-type receptors using simultaneous PET/MRI imaging. *European Journal of Nuclear Medicine and Molecular Imaging* **44**, (2017).

88. Sandiego, C. M. *et al.* Reference region modeling approaches for amphetamine challenge studies with [11C]FLB 457 and PET. *J Cereb Blood Flow Metab* **35**, 623–629 (2015).

89. Slifstein, M. *et al.* Deficits in prefrontal cortical and extrastriatal dopamine release in schizophrenia: a positron emission tomographic functional magnetic resonance imaging study. *JAMA Psychiatry* **72**, 316–324 (2015).

90. Smith, C. T. *et al.* Partial-volume correction increases estimated dopamine D2-like receptor binding potential and reduces adult age differences. *J Cereb Blood Flow Metab* **39**, 822–833 (2019).

91. Zakiniaeiz, Y. *et al.* Sex differences in amphetamine-induced dopamine release in the dorsolateral prefrontal cortex of tobacco smokers. *Neuropsychopharmacology* **44**, 2205–2211 (2019).

92. Dukart, J. *et al.* Cerebral blood flow predicts differential neurotransmitter activity. *Sci Rep* **8**, 4074 (2018).

93. Sanchez-Rangel, E. *et al.* Norepinephrine transporter availability in brown fat is reduced in obesity: a human PET study with [11C] MRB. *Int J Obes* **44**, 964–967 (2020).

94. Ding, Y.-S. *et al.* PET imaging of the effects of age and cocaine on the norepinephrine transporter in the human brain using (S,S)-[(11)C]O-methylreboxetine and HRRT. *Synapse* **64**, 30–38 (2010).

95. Belfort-DeAguiar, R. *et al.* Noradrenergic Activity in the Human Brain: A Mechanism Supporting the Defense Against Hypoglycemia. *The Journal of Clinical Endocrinology & Metabolism* **103**, 2244–2252 (2018).

96. Li, C. R. *et al.* Decreased norepinephrine transporter availability in obesity: Positron Emission Tomography imaging with (S,S)-[(11)C]O-methylreboxetine. *Neuroimage* **86**, 306–310 (2014).

97. Savli, M. *et al.* Normative database of the serotonergic system in healthy subjects using multi-tracer PET. *NeuroImage* **63**, 447–459 (2012).

98. Gallezot, J.-D. *et al.* Kinetic modeling of the serotonin 5-HT(1B) receptor radioligand [(11)C]P943 in humans. *J Cereb Blood Flow Metab* **30**, 196–210 (2010).

99. Matuskey, D. *et al.* Reductions in brain 5-HT1B receptor availability in primarily cocaine-dependent humans. *Biol Psychiatry* **76**, 816–822 (2014).

100. Murrough, J. W. *et al.* The effect of early trauma exposure on serotonin type 1B receptor expression revealed by reduced selective radioligand binding. *Arch Gen Psychiatry* **68**, 892–900 (2011).

101. Pittenger, C. *et al.* OCD is associated with an altered association between sensorimotor gating and cortical and subcortical 5-HT1b receptor binding. *J Affect Disord* **196**, 87–96 (2016).

102. Saricicek, A. *et al.* Test-retest reliability of the novel 5-HT1B receptor PET radioligand [11C]P943. *Eur J Nucl Med Mol Imaging* **42**, 468–477 (2015).

103. Beliveau, V. *et al.* A High-Resolution In Vivo Atlas of the Human Brain's Serotonin System. *The Journal of Neuroscience* **37**, 120 (2017).

104. Radhakrishnan, R. *et al.* In vivo 5-HT6 and 5-HT2A receptor availability in antipsychotic treated schizophrenia patients vs. unmedicated healthy humans measured with [11C]GSK215083 PET. *Psychiatry Res Neuroimaging* **295**, 111007 (2020).

105. Radhakrishnan, R. *et al.* Age-Related Change in 5-HT6 Receptor Availability in Healthy Male Volunteers Measured with 11C-GSK215083 PET. *J Nucl Med* **59**, 1445–1450 (2018).

106. Hillmer, A. T. *et al.* Imaging of cerebral α4β2* nicotinic acetylcholine receptors with (-)-[(18)F]Flubatine PET: Implementation of bolus plus constant infusion and sensitivity to acetylcholine in human brain. *Neuroimage* **141**, 71–80 (2016).

107. Baldassarri, S. R. *et al.* Use of Electronic Cigarettes Leads to Significant Beta2-Nicotinic Acetylcholine Receptor Occupancy: Evidence From a PET Imaging Study. *Nicotine Tob Res* **20**, 425–433 (2018).

108. Naganawa, M. *et al.* First-in-Human Assessment of 11C-LSN3172176, an M1 Muscarinic Acetylcholine Receptor PET Radiotracer. *J Nucl Med* **62**, 553–560 (2021).

109. Bedard, M.-A. *et al.* Brain cholinergic alterations in idiopathic REM sleep behaviour disorder: a PET imaging study with 18F-FEOBV. *Sleep Med* **58**, 35–41 (2019).

110. Aghourian, M. *et al.* Quantification of brain cholinergic denervation in Alzheimer's disease using PET imaging with [18F]-FEOBV. *Mol Psychiatry* **22**, 1531–1538 (2017).

111. Smart, K. *et al.* Sex differences in [11C]ABP688 binding: a positron emission tomography study of mGlu5 receptors. *Eur J Nucl Med Mol Imaging* **46**, 1179–1183 (2019).

112. DuBois, J. M. *et al.* Characterization of age/sex and the regional distribution of mGluR5 availability in the healthy human brain measured by high-resolution [(11)C]ABP688 PET. *Eur J Nucl Med Mol Imaging* **43**, 152–162 (2016).

113. Galovic, M. *et al.* In vivo NMDA receptor function in people with NMDA receptor antibody encephalitis. 2021.12.04.21267226 Preprint at https://doi.org/10.1101/2021.12.04.21267226 (2021).

114. Galovic, M. *et al.* Validation of a combined image derived input function and venous sampling approach for the quantification of [18F]GE-179 PET binding in the brain. *Neuroimage* **237**, 118194 (2021).

115. Nørgaard, M. *et al.* A High-Resolution In Vivo Atlas of the Human Brain's Benzodiazepine Binding Site of GABA A Receptors. *NeuroImage* **232**, 117878 (2021).

116. Gallezot, J.-D. *et al.* Determination of receptor occupancy in the presence of mass dose: [11C]GSK189254 PET imaging of histamine H3 receptor occupancy by PF-03654746. *J Cereb Blood Flow Metab* **37**, 1095–1107 (2017).

117. D'Souza, D. C. *et al.* Rapid Changes in CB1 Receptor Availability in Cannabis Dependent Males after Abstinence from Cannabis. *Biol Psychiatry Cogn Neurosci Neuroimaging* **1**, 60–67 (2016).

118. Neumeister, A. *et al.* Positron emission tomography shows elevated cannabinoid CB1 receptor binding in men with alcohol dependence. *Alcohol Clin Exp Res* **36**, 2104–2109 (2012).

119. Normandin, M. D. *et al.* Imaging the cannabinoid CB1 receptor in humans with [11C]OMAR: assessment of kinetic analysis methods, test-retest reproducibility, and gender differences. *J Cereb Blood Flow Metab* **35**, 1313–1322 (2015).

120. Ranganathan, M. *et al.* Reduced Brain Cannabinoid Receptor Availability in Schizophrenia. *Biol Psychiatry* **79**, 997–1005 (2016).

121. Kantonen, T. *et al.* Interindividual variability and lateralization of μ-opioid receptors in the human brain. *NeuroImage* **217**, 116922 (2020).

122. Hansen, J. Y. *et al.* Integrating multimodal and multiscale connectivity blueprints of the human cerebral cortex in health and disease. *PLOS Biology* **21**, e3002314 (2023).

123. Paquola, C. *et al.* Microstructural and functional gradients are increasingly dissociated in transmodal cortices. *PLoS Biology* **17**, e3000284 (2019).

124. Amunts, K. *et al.* BigBrain: An Ultrahigh-Resolution 3D Human Brain Model. *Science* **340**, 1472–1475 (2013).

125. Yarkoni, T., Poldrack, R. A., Nichols, T. E., Van Essen, D. C. & Wager, T. D. Large-scale automated synthesis of human functional neuroimaging data. *Nature Methods* **8**, 665–670 (2011).

126. Poldrack, R. *et al.* The Cognitive Atlas: Toward a Knowledge Foundation for Cognitive Neuroscience. *Frontiers in Neuroinformatics* **5**, (2011).
