# Supplementary material for: Competitive interactions shape mammalian brain network dynamics and computation
Source: Nat Neurosci. 2026 Mar 11;29(4):915–33. doi: 10.1038/s41593-026-02205-3 (PMC13061630; doi:10.1038/s41593-026-02205-3)
Supplement: Supplementary file 2 — Reporting Summary [file 41593_2026_2205_MOESM2_ESM.pdf]

nature portfolio

Corresponding author(s): Andrea Luppi

Last updated by author(s): Dec 30, 2025

Reporting Summary

Nature Portfolio wishes to improve the reproducibility of the work that we publish. This form provides structure for consistency and transparency in reporting. For further information on Nature Portfolio policies, see our Editorial Policies and the Editorial Policy Checklist.

Statistics

For all statistical analyses, confirm that the following items are present in the figure legend, table legend, main text, or Methods section.

n/a	Confirmed	
☐	☒	The exact sample size (*n*) for each experimental group/condition, given as a discrete number and unit of measurement
☐	☒	A statement on whether measurements were taken from distinct samples or whether the same sample was measured repeatedly
☐	☒	The statistical test(s) used AND whether they are one- or two-sided<br>*Only common tests should be described solely by name; describe more complex techniques in the Methods section.*
☒	☐	A description of all covariates tested
☒	☐	A description of any assumptions or corrections, such as tests of normality and adjustment for multiple comparisons
☐	☒	A full description of the statistical parameters including central tendency (e.g. means) or other basic estimates (e.g. regression coefficient) AND variation (e.g. standard deviation) or associated estimates of uncertainty (e.g. confidence intervals)
☐	☒	For null hypothesis testing, the test statistic (e.g. *F*, *t*, *r*) with confidence intervals, effect sizes, degrees of freedom and *P* value noted<br>*Give P values as exact values whenever suitable.*
☒	☐	For Bayesian analysis, information on the choice of priors and Markov chain Monte Carlo settings
☒	☐	For hierarchical and complex designs, identification of the appropriate level for tests and full reporting of outcomes
☐	☒	Estimates of effect sizes (e.g. Cohen's *d*, Pearson's *r*), indicating how they were calculated

Our web collection on statistics for biologists contains articles on many of the points above.

Software and code

Policy information about availability of computer code

Data collection	NeuroSynth is available at https://neurosynth.org/.
Data analysis	MATLAB and C++ code for the Hopf model with cooperative and competitive interactions is provided at https://github.com/Hana-Ali/competitive-cooperative-hopf.git. MATLAB/Octave and Python code (v1.0) to compute measures of Integrated Information Decomposition of timeseries with the Gaussian MMI solver, is available at https://github.com/Imperial-MIND-lab/integrated-info-decomp. The conn2res Python toolbox for reservoir computing (v1.0.0) is available at: https://github.com/netneurolab/conn2res. The Brain Connectivity Toolbox code (version 2019-03-03) used for graph-theoretical analyses and network randomisation is freely available online (https://sites.google.com/site/bctnet/). The Python processing for PreClinical data pipeline, Pypreclin version 1.0.1, is freely available at https://github.com/neurospin/pypreclin. FMRIB Software Library (FSL) is freely available online (http://www.fmrib.ox.ac.uk/fsl/; version accessed February 4, 2018). The CONN toolbox version 17f is freely available at http://www.nitrc.org/projects/conn/. DSI Studio is freely available at https://dsi-studio.labsolver.org/. The Java Information Dynamics Toolbox v1.5 is freely available online: (https://github.com/jlizier/jidt). The latest version of BigBrainWarp toolbox is freely available online (https://bigbrainwarp.readthedocs.io). The abagen toolbox (v0.1.4) for processing of the AHBA human transcriptomic dataset is available at https://abagen.readthedocs.io/. The neuromaps toolbox (v0.0.5) is available at https://netneurolab.github.io/neuromaps/.

For manuscripts utilizing custom algorithms or software that are central to the research but not yet described in published literature, software must be made available to editors and reviewers. We strongly encourage code deposition in a community repository (e.g. GitHub). See the Nature Portfolio guidelines for submitting code & software for further information.

Data

Policy information about availability of data

All manuscripts must include a data availability statement. This statement should provide the following information, where applicable:

- Accession codes, unique identifiers, or web links for publicly available datasets
- A description of any restrictions on data availability
- For clinical datasets or third party data, please ensure that the statement adheres to our policy

The Human Connectome Project functional and structural datasets are freely available from http://www.humanconnectome.org/. Macaque functional MRI data are available from the PRIMatE Data Exchange (PRIME-DE) through the Neuroimaging Informatics Tools and Resources Clearinghouse (NITRC; http://fcon_1000.projects.nitrc.org/indi/indiPRIME.html). The macaque connectome is available on Zenodo at https://doi.org/10.5281/zenodo.1471588. The CoCoMac database on which it is based, is also available online at http://cocomac.g-node.org/main/index.php?. Mouse functional and structural connectome data are available from author A.G. NeuroSynth is available at https://neurosynth.org/. The original macaque cortical gene expression and cell type density data from 111 are available at https://macaque.digital-brain.cn/spatial-omics. The dataset is provided by Brain Science Data Center, Chinese Academy of Sciences (https://braindatacenter.cn/). The original macaque receptor density data from autoradiography are available from https://balsa.wustl.edu/study/P2Nql and https://search.kg.ebrains.eu/instances/de62abc1-7252-4774-9965-5040f5e8fb6b 116. The original map of macaque intracortical myelination from T1w:T2w ratio from 116 is available at https://balsa.wustl.edu/study/P2Nql. Mouse cell type data are available as described in 114. Human gene expression data 112 are available from the Allen Human Brain Atlas at http://human.brain-map.org/static/download. Mouse gene expression data 110 are available at https://mouse.brain-map.org/. .Human cell type data are available as described in 113. Human receptor density data are available online from the neuroimaps toolbox. Source data are provided as Source Data files.

Research involving human participants, their data, or biological material

Policy information about studies with human participants or human data. See also policy information about sex, gender (identity/presentation), and sexual orientation and race, ethnicity and racism.

Reporting on sex and gender	Gender data were not collected separately from biological sex data. Analyses included invididuals of both sexes, as sex-related differences were not among the research hypotheses.
Reporting on race, ethnicity, or other socially relevant groupings	No groupings by race, ethnicity, or socieconomic status were performed. For the HCP dataset, recruiting efforts were used by the HCP consortium to ensure that participants broadly reflect the ethnic and racial composition of the U.S. population as represented in the 2000 decennial census.
Population characteristics	HCP data: 100 healthy participants (54 females and 46 males), mean age = 29.1 + 3.7 years.
Recruitment	No new data were collected for this study. See Van Essen et al., 2012 for recruitment of HCP subjects.
Ethics oversight	The Institutional Review Board at Washington University in St. Louis approved the study protocol.

Note that full information on the approval of the study protocol must also be provided in the manuscript.

Field-specific reporting

Please select the one below that is the best fit for your research. If you are not sure, read the appropriate sections before making your selection.

☒ Life sciences ☐ Behavioural & social sciences ☐ Ecological, evolutionary & environmental sciences

For a reference copy of the document with all sections, see nature.com/documents/nr-reporting-summary-flat.pdf

Life sciences study design

All studies must disclose on these points even when the disclosure is negative.

Sample size	We used previously collected data. The set of 100 unelated human participants from HCP is widely used, and he sample sizes for each non-human species (n=14 macaques; n=10 mice) are in line with field standards and our own and others' previous publications.
Data exclusions	No exclusions among the HCP 100 unrelated subjects. Also no animals from the mouse dataset were excluded. For the macaque dataset, we only used the n=10 animals who had awake scans. Only Neurosynth terms inthe intersection with the Cognitive Atlas were retained, since Neurosynth terms include a very wide variety of terms including regions("dorsolateral") and clinical terms {"ADHD"), many of which are not relevant to the research question of defining cognitive topographies.
Replication	We replicated all our results in three distinct mammalian species (human, macaque, mouse). For the human data, we replicated results with vs without Global Singal Regression. We also replicated results using a different, more fine-grained parcellation of the cortex (Schaefer-200 with addition of subcortical structures from the Tian atlas). We also replicate our results using asymmetric versions of the macaque and mouse connectomes. This is a computational study so replications did not involve acquisition of new data, but rather re-running of the analysis with different parameters.
Randomization	Not applicable: no experimental groups.

Blinding	Not applicable. Human, macaques, and mice were all scanned at rest, without any experimental manipulation to be blinded to. There was no group allocation.

Reporting for specific materials, systems and methods

We require information from authors about some types of materials, experimental systems and methods used in many studies. Here, indicate whether each material, system or method listed is relevant to your study. If you are not sure if a list item applies to your research, read the appropriate section before selecting a response.

Materials & experimental systems

n/a	Involved in the study
☒	☐ Antibodies
☒	☐ Eukaryotic cell lines
☒	☐ Palaeontology and archaeology
☐	☒ Animals and other organisms
☒	☐ Clinical data
☒	☐ Dual use research of concern
☒	☐ Plants

Methods

n/a	Involved in the study
☒	☐ ChIP-seq
☒	☐ Flow cytometry
☐	☒ MRI-based neuroimaging

Animals and other research organisms

Policy information about studies involving animals; ARRIVE guidelines recommended for reporting animal research, and Sex and Gender in Research

Laboratory animals	Macaque data: Functional MRI data were obtained from 10 exemplars of Macaca Mulatta, out of 14 (12 male, 2 female); Age distribution: 3.9-13.14 years; Weight distribution: 7.2-18 kg (full sample description available online: http://fcon_1000.projects.nitrc.org/indi/PRIME/files/newcastle.csv and http://fcon_1000.projects.nitrc.org/indi/PRIME/newcastle.html).<br><br>Mouse data: Adult (< 6 months old) male C57BL/6J mice were used throughout the study.
Wild animals	No wild animals were used.
Reporting on sex	The macaque dataset included both male and female animals. The mouse data included only male animals.
Field-collected samples	Not used.
Ethics oversight	Macaque dataset: All of the animal procedures performed were approved by the UK Home Office and comply with the Animal Scientific Procedures Act (1986) on the care and use of animals in research and with the European Directive on the protection of animals used in research (2010/63/EU).<br><br>Mouse dataset: experiments were conducted in accordance with the Italian law (DL 26/214, EU 63/2010, Ministero della Sanita, Roma) and with the National Institute of Health recommendations for the care and use of laboratory animals

Note that full information on the approval of the study protocol must also be provided in the manuscript.

Plants

Seed stocks	Plants were not used.
Novel plant genotypes	Plants were not used.
Authentication	Plants were not used.

Magnetic resonance imaging

Experimental design

Design type	Resting-state for each species.

Design specifications

HCP dataset: Two sessions of 15 min resting-state fMRI were acquired (here, we only used data acquired in the LR encoding direction)

Macaque dataset: Resting-state scanning was performed for 21.6 minutes, with awake animals.

Mouse dataset: awake resting-state scanning for a total time of 32 minutes.

Behavioral performance measures

No behavioural measures were collected during scanning in any of the three species. HCP also involved separate task-based scans that are not analysed in the present study.

Acquisition

Imaging type(s)

HCP dataset: fMRI and diffusion MRI.
Macaque and mouse datasets: fMRI

Field strength

HCP datasets: 3T. Macaque dataset: 4.7T. Mouse dataset: 7T.

Sequence & imaging parameters

HCP dataset: resting-state fMRI: gradient-echo EPI, TR= 720 ms, TE= 33.1 ms, flip angle = 52°, FOV= 208 × 180, voxel size = 2 mm isotropic.

Macaque dataset: TR of 2600ms, 17ms TE, voxels size 1.22 x 1.22 x 1.24. Effective Echo Spacing of 0.63ms. Phase Encoding Direction: Encoded in columns.

Mouse: Awake rsfMRI scans were acquired using a single-shot echo planar imaging (EPI) sequence with the following parameters: TR/TE=1000/15 ms, flip angle=60 degrees, matrix=100 x 100, FOV=2.3 x 2.3 cm, 18 coronal slices (voxel-size 230 x 230 x 600 mm), slice thickness=600 mm and 1920 time points, for a total time of 32 minutes.

Area of acquisition

Whole brain

Diffusion MRI ☒ Used ☐ Not used

Parameters

The spatial resolution was 1.25 mm isotropic. TR=5500ms, TE=89.50ms. The b-values were 1000, 2000, and 3000 s/mm2. The total number of diffusion sampling directions was 90, 90, and 90 for each of the shells in addition to 6 b0 images.

Preprocessing

Preprocessing software

HCP dataset fMRI: the minimally preprocessed HCP functional data were used, with further denoising using the CONN toolbox V17f (please see Glasser et al., 2013 (NeuroImage) for details on HCP minimal preprocessing pipelines).

HCP dataset diffusion MRI: DSI Studio was used on the minimally preprocessed HCP diffusion data.

Macaque fMRI: Pypreclin pipelines for preprocessing (lease see Grigis et al., 2020 (NeuroImage) for details). Briefly, it includes the following steps: (i) Slice-timing correction. (ii) Correction for the motion-induced, time-dependent B0 inhomogeneities. (iii) Reorientation from acquisition position to template; here, we used the recently developed National Institute of Mental Health Macaque Template (NMT). (iv) Realignment to the middle volume using FSL MCFLIRT function. Subsequent denoising was performed using the CONN toolbox V17f.

Mouse dataset: RsfMRI timeseries were then time despiked (3dDespike, AFNI), motion corrected (MCFLIRT, FSL), skull stripped (FAST, FSL) and spatially registered (ANTs registration suite).

Normalization

HCP dataset FMRI: please see Glasser et al., 2013 (NeuroImage) for details on HCP minimal preprocessing pipelines.

HCP dataset diffusion MRI: DWI data were then reconstructed using q-space diffeomorphic reconstruction (QSDR), as implemented in DSI Studio (www.dsi-studio.labsolver.org). QSDR first reconstructs diffusion-weighted images in native space and computes the quantitative anisotropy (QA) in each voxel. These QA values are used to warp the brain to a template QA volume in Montreal Neurological Institute (MNI) space using the statistical parametric mapping (SPM) nonlinear registration algorithm. A diffusion sampling length ratio of 2.5 was used, and the output resolution was 1 mm.

Macaque dataset: as part of the Pypreclin pipeline, the following steps were performed: (v) Normalisation and masking using Joe's Image Program (JIP) -align routine (http://www.nmr.mgh .harvard.edu/~jbm/jip/, Joe Mandeville, Massachusetts General Hospital, Harvard University, MA, USA), which is specifically designed for preclinical studies: the normalization step aligns (affine) and warps (non-linear alignment using distortion field) the anatomical data into a generic template space. (vi) B1 field correction for low-frequency intensity non-uniformities present in the data. (vii) Coregistration of functional and anatomical images, using JIP -align to register the mean functional image (moving image) to the anatomical image (fixed image) by applying a rigid transformation. The anatomical brain mask was obtained by warping the template brain mask using the deformation field previously computed during the normalization step. Then, the functional images were aligned with the template space by composing the normalization and coregistration spatial transformations.

Mouse dataset: data were spatially registered (ANTs registration suite) to an in-house mouse brain template with a spatial resolution of 0.23 x 0.23 x 0.6mm3.

Normalization template

HCP dataset: MNI152 template. Please see original study for details. Human brains were parcellated into 100 cortical regions of interest from the Schaefer atlas.

	Macaque data: we used the National Institute of Mental Health Macaque Template (NMT): a high-resolution template of the average macaque brain generated from in vivo MRI of 31 rhesus macaques (Macaca mulatta). Macaque functional data were parcellated according to the 82-ROI “Regional Mapping” cortical atlas of Kotter and Wanke 158, nonlinearly registered to the NMT template used for preprocessing.<br>Mouse data: in-house mouse brain template; data were parcellated into 72 cortical symmetric regions from the Allen Mouse Brain Atlas (CCFv3).
Noise and artifact removal	The anatomica CompCor (aCompCor) method was used for denoising of both human and macaque fMRI data.<br>The aCompCor method involves regressing out of the functional data the following confounding effects: the first five principal components attributable to each individual’s white matter signal, and the first five components attributable to individual cerebrospinal fluid (CSF) signal; six subject-specific realignment parameters (three translations and three rotations) as well as their first- order temporal derivatives . Linear detrending was also applied, and the subject-specific denoised BOLD signal timeseries were band-pass filtered to eliminate both low-frequency drift effects and high-frequency noise, thus retaining frequencies between 0.008 and 0.09 Hz.<br><br>For macaques, white matter and CSF masks were obtained from the corresponding probabilistic tissue maps of the high-resolution NMT template (eroded by 1 voxel); their first five principal components were regressed out of the functional data, as well as linear trends and 6 motion parameters (3 translations and 3 rotations) and their first derivatives. Following previous work on macaque functional MRI (see e.g. Barttfeld et al., 2015 (PNAS)), data were bandpass-filtered in the range of 0.0025-0.05 Hz. When comparing directly between human and macaque data, results were also replicated using the same bandpass filter of 0.008-0.09Hz used for human data.<br><br>Mouse: Denoising involved the regression of 25 nuisance parameters. These were: average cerebral spinal fluid signal plus 24 motion parameters determined from the 3 translation and rotation parameters estimated during motion correction, their temporal derivatives and corresponding squared regressors. No global signal regression was employed. In-scanner head motion was quantified via calculations of frame-wise displacement (FD). Average FD levels in awake conditions were comparable to those obtained in anesthetized animals (halothane) under artificial ventilation ($p = 0.13$, t-test) 85. To rule out a contribution of residual head-motion, we further introduced frame-wise fMRI scrubbing (FD > 0.075 mm). The resulting time series were band-pass filtered (0.01-0.1 Hz band) and then spatially smoothed with a Gaussian kernel of 0.5 mm full width at half maximum. The timeseries were trimmed to ensure that the same number of timepoints were included for all animals, resulting in 1414 volumes per animal.
Volume censoring	Scrubbing of mouse data (FD > 0.075).

Statistical modeling & inference

Model type and settings	We obtained two model-derived matrices of effective connectivity for each subject in each dataset: one with negative weights, and one without. Statistical significance was assessed using a resampling-based, paired-samples t-test. This non-parametric implementation of the test ensures robustness to violations of the normality assumption, which was not formally tested. Effect sizes are provided as Hedge’s measure of standardised difference g, which is analogous to Cohen’s d, but recommended for smaller sample sizes such as the ones available in the present study. For the comparison of goodness-of-fit of the model, we used the correlation between the empirical and simulated vectorised FC matrices.
Effect(s) tested	We compared effective connectivity and activity generated from computational models with or without negative weights.

Specify type of analysis: ☒ Whole brain ☐ ROI-based ☐ Both

Statistic type for inference<br>(See Eklund et al. 2016)	Whole-brain analyses were used
Correction	Not applicable: no mass-univariate analysis was performed.

Models & analysis

n/a	Involved in the study
☐	☒ Functional and/or effective connectivity
☐	☒ Graph analysis
☒	☐ Multivariate modeling or predictive analysis

Functional and/or effective connectivity	Functional connectivity was computed as the pearson correlation between regional time-series of activity, for both the real and simulated data. Effective connectivity was inferred from a connectome-based Hopf model (with or without negative weights). The model was iteratively run with the updated GEC until the fit converged to a stable value.
Graph analysis	The model is a network-based model, so all data are network-based. However, we also explicitly ce computed a single value of mean clustering coefficient for each effective connectivity amtrix (one per subject in each dataset), both with and without negative weights. We also computed the modularity.
